# Informing health system planning for biomarker-based treatment: statistical prevalence projections for solid cancers with key pan-tumour biomarkers (dMMR, MSI, high TMB) in Australia to 2042

**DOI:** 10.1016/j.lanwpc.2025.101537

**Published:** 2025-04-04

**Authors:** Yoon-Jung Kang, Qingwei Luo, Joachim Worthington, Anna Kelly, Jeff Cuff, John Zalcberg, Karen Canfell, Julia Steinberg

**Affiliations:** a The Daffodil Centre, The University of Sydney, A Joint Venture with Cancer Council NSW, NSW, 2011, Australia; b Research Advocate, The Daffodil Centre, NSW, 2011, Australia; c Faculty of Science Biotech and Biomolecular Science, University of New South Wales, NSW, 2033, Australia; d Department of Medical Oncology, Alfred Health and School of Public Health and Preventive Medicine, Faculty of Medicine, Monash University, VIC, 3004, Australia; e School of Public Health, The University of Sydney, NSW, 2050, Australia

**Keywords:** Pan-tumour biomarker, Mismatch repair deficiency, Microsatellite instability, Tumour mutational burden, Cancer prevalence

## Abstract

**Background:**

Targeted cancer treatment based on mismatch repair deficiency (dMMR), microsatellite instability (MSI), or high tumour mutational burden (TMB) holds promise for improving patient outcomes, but presents substantial healthcare costs.

**Methods:**

Using validated statistical methods, we projected 1-year to 5-year prevalence of individuals diagnosed with solid tumours exhibiting these biomarkers in Australia to 2042, for all solid cancers combined and 23 individual cancer types/groups, and separately for all stages combined, advanced disease at diagnosis (here, distant metastasis/lymph node involvement), and advanced disease after progression post-diagnosis.

**Findings:**

The 5-year prevalence of individuals diagnosed with any solid cancer regardless of biomarker status is estimated to increase by 54·2%, from 438,346 in 2018 to 675,722 in 2042 (advanced disease at diagnosis: by 37·6% from 109,855 to 151,199), primarily due to population growth and ageing. The 5-year prevalence of individuals whose tumours exhibit a biomarker is estimated to increase accordingly, e.g. for advanced disease at diagnosis, from 3983 to 5448 for dMMR, from 2484 to 3553 for MSI, and from 13,310 to 17,893 for high TMB (representing 3·6%, 2·3% and 11·8% of 5-year prevalence of individuals with advanced disease at diagnosis, respectively; noting considerable overlap in the presence of these biomarkers).

**Interpretation:**

We present the first long-term projections for cancer prevalence associated with key pan-tumour biomarkers in Australia, to inform health policy and healthcare planning for targeted therapies.

**Funding:**

Medical Research Future Fund—Preventive and Public Health Research Initiative—2019 Targeted Health System and Community Organization Research Grant Opportunity (MRF1200535), 10.13039/501100001171Cancer Institute NSW Career Development Fellowship (2022/CDF1154), 10.13039/501100000925National Health and Medical Research Council of Australia Investigator Grant (APP1194679).

## Graphical abstract

**Figure:**
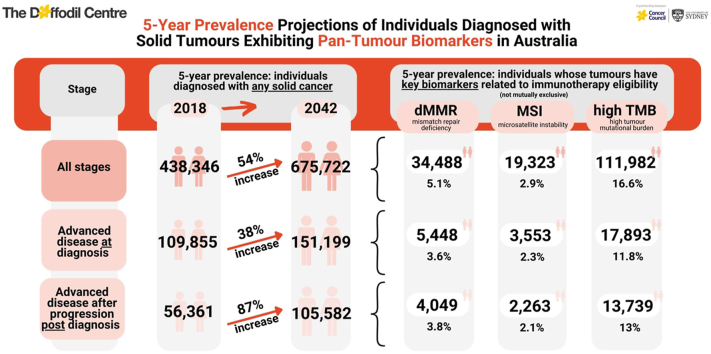

**Research in context**

**Evidence before this study**

There is increasing interest in targeting cancer treatment to molecular features of different tumours, with “pan-tumour biomarkers” denoting informative molecular features that are agnostic of the cancer site. In particular, immune checkpoint inhibitors for the treatment of advanced solid tumours with mismatch repair deficiency (dMMR), microsatellite instability (MSI), or high tumour mutational burden (TMB) have the potential to improve patient outcomes, but also incur high costs to the healthcare system. Therefore, long-term projections of cancer prevalence related to these key pan-tumour biomarkers are important to support health policy decisions and healthcare planning.

We searched Medline and Embase databases on 16 August 2024 for projections of cancer prevalence related to pan-tumour biomarkers. We used the search terms “(forecast$ or projecte$ or projection$ or predict$ or extrapolat$ or estimat$).tw” and “prevalence.tw” and “biomarker$.tw” and “(cancer∗ or carcinoma∗ or tumo?r∗ or neoplas∗ or malignan∗ or pan$cancer or pan$tumour or pan$tumor).tw”. We did not impose any date or language restrictions, excluding conference abstracts only. The search did not identify any projections for cancer prevalence associated with pan-tumour biomarkers in Australia or internationally.

**Added value of this study**

We used validated statistical methods to project 1-year to 5-year cancer prevalence in Australia to 2042, for all solid cancers combined and 23 individual cancer types/groups. We also specifically obtained prevalence estimates for individuals with tumours exhibiting dMMR, MSI, or high TMB (≥10 mutations/Mb). All analyses were conducted separately for all stages combined and advanced disease at diagnosis, with exploratory estimates for advanced disease after progression post-diagnosis. We completed extensive validation of projections against observed data to demonstrate their reliability.

We estimate that the 5-year prevalence of individuals diagnosed with any solid cancer regardless of tumour biomarker status is estimated to increase from 2018 to 2042, for all stages combined (54%), advanced disease at diagnosis (38%), and advanced disease after progression post-diagnosis (87%), largely due to population growth and ageing. The prevalence of individuals whose tumours exhibit dMMR, MSI and high TMB is estimated to increase accordingly. Detailed estimates are provided for different calendar periods and each biomarker (noting considerable overlap in their presence).

**Implications of all the available evidence**

These detailed prevalence projections can support future health policy decisions, budget impact forecasts, and healthcare planning for a broad range of different scenarios related to biomarker-based treatment. This includes consideration of potential future changes in clinical practice such as use of targeted treatments for earlier disease stages, different treatment duration, or separate assessments for different cancer types. Notably, we established a systematic, comprehensive approach to estimate cancer prevalence related to key biomarkers, which can be readily extended to other biomarkers and countries, supporting efforts to improve health outcomes.

## Introduction

The burden of cancer is rising steadily worldwide, and cancer remains the leading cause of premature mortality in many countries including Australia.^1^ As part of advances in precision medicine, there is increasing interest in improving cancer outcomes through targeted cancer treatment. Such targeted treatment is based on molecular features of the tumour rather than the tissue of origin, with tissue-agnostic markers called “pan-tumour biomarkers”.^2^ In particular, immune checkpoint inhibitors for the treatment of adult and paediatric patients with tumours exhibiting mismatch repair deficiency (dMMR), microsatellite instability (MSI), or high tumour mutational burden (TMB, e.g. ≥10 mutations/Mb) have been approved or are under consideration in several countries (including Australia, Japan, the USA, and the European Union).^3^, ^4^, ^5^, ^6^ These biomarker-based treatments have the potential to improve patient outcomes, but also incur high costs to the healthcare system^7^ (albeit noting that alternative less effective treatments would also incur substantial costs, likely requiring more treatment changes and/or longer hospitalisation; and that checkpoint inhibitors can yield cure or long-term remission for some patients that might not have been achieved by other treatments, which is reflected in relevant favourable health technology assessments).

As an illustration of these substantial costs and rising utilisation of immune checkpoint inhibitors in the last decade, the global immune checkpoint inhibitors market is expected to grow from US$19·9 billion in 2021 to US$46·3 billion in 2026.^8^ In the 2022–23 financial year in Australia, two immune checkpoint inhibitors (pembrolizumab and nivolumab) with treatment subsidised through the Pharmaceutical Benefits Scheme (PBS) were ranked the fourth- and fifth-highest medicines by total government cost,^7^ with a total cost of AUD$858·5 million (Table S1) and an increase of 29% from 2019–2020 based on published prices.^7^^,^^9^ Therefore, long-term projections of cancer prevalence related to these pan-tumour biomarkers are important to support planning and budget impact forecasts for utilisation of both existing and potential new therapies, which are needed to inform health technology assessments for biomarker-based therapies. Here, prevalence is defined as the number of individuals alive in a population who have been diagnosed with cancer, aggregating across the duration of survival. For example, 5-year prevalence of colorectal cancer in 2020 is defined as the number of individuals who are alive in 2020 and were diagnosed with colorectal cancer during the preceding 5 years (in this example, 2015–2020).^10^^,^^11^ This measure provides crucial information for health system planning; it is separate from incidence, which focuses on the number of new cancer diagnoses in a specified period.^12^

Currently, approvals of immune checkpoint inhibitors as a pan-tumour biomarker-based treatment focus on individuals with advanced disease, including solid tumours that are metastatic, unresectable, or have progressed following prior treatment with limited satisfactory alternatives.^3^^,^^4^^,^^6^ However, these treatment options might also be applicable to earlier disease stages in the future. Thus, prevalence projections for all stages as well as for advanced disease are needed to provide comprehensive insights for future heath technology assessments. Moreover, to provide information that can support assessments for a broad range of potential future scenarios regarding treatment duration, it is important to examine cancer prevalence for different post-diagnosis periods, e.g. 1 year to 5 years post-diagnosis.

Notably, the long-term projections required for health technology assessments are subject to inherent uncertainty, including future changes in cancer incidence, stage distribution, and survival. Especially in view of these uncertainties, it is vital to establish a rigorous approach to projections with incorporation of latest available data, clear and explicit assumptions, extensive validation, and impartial analysis independent of sponsors for drug approvals.

Here, we use validated statistical methods to provide the first long-term projections of cancer prevalence associated with key pan-tumour biomarkers in Australia to 2042, separately for dMMR, MSI, and high TMB (noting there is considerable overlap in the presence of these biomarkers, with e.g. tumours with dMMR or MSI also predominantly exhibit high TMB^13^). We estimate 1-year to 5-year prevalence for all solid cancers combined and 23 cancer types/groups. Estimates are presented for all stages combined as well as for advanced disease at diagnosis. Separate exploratory analyses were also performed to estimate prevalence of individuals with advanced disease after progression post-diagnosis. In this study, advanced disease includes both distant metastasis or lymph node involvement, in line with increasing consideration of biomarker-based treatment for the latter (regulatory approval for immune checkpoint inhibitors has already extended beyond metastatic disease for e.g. melanoma and non-small cell lung cancer,^14^ with clinical practice guidelines also increasingly including immune checkpoint inhibitors for e.g. locally advanced colorectal, gastric, and pancreatic cancers,^15^, ^16^, ^17^ see Table S2 for overview of current Australian approvals and key international guidelines). Finally, using the prevalence estimates, we also present an illustration of potential costs for a treatment linked to these biomarkers.

## Methods

**Fig. 1:**
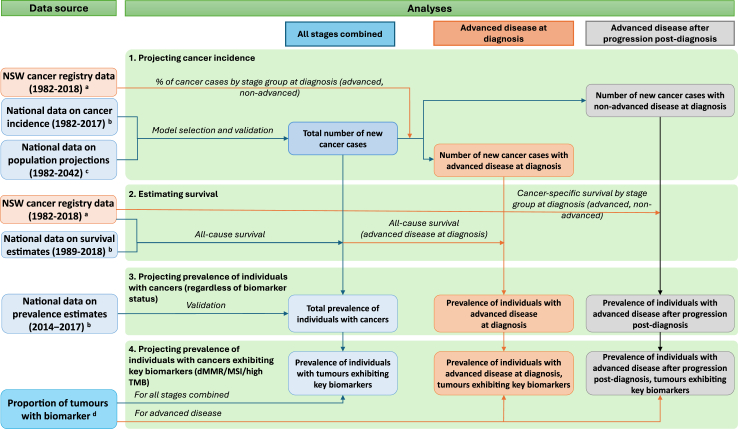
**Study approach and data sources**. NSW, New South Wales; dMMR, mismatch repair deficiency; MSI, microsatellite instability; high TMB, high tumour mutational burden (≥10 mutations/Mb). ^a^Individual-level data from the NSW Cancer Institute's Enduring Cancer Data Linkage (CanDLe). ^b^Tabulated data from the Australian Institute of Health and Welfare (AIHW). ^c^Tabulated data from the Australian Bureau of Statistics (ABS). ^d^Obtained from our recent scoping review and meta-analysis.^18^

As illustrated in Fig. 1, this study consists of four analysis steps: 1) projecting cancer incidence using a statistical modelling approach; 2) estimating survival at 1–5 years after diagnosis; 3) projecting prevalence of individuals with cancers regardless of biomarker status, as a function of incidence and survival estimates; and 4) projecting prevalence of individuals whose tumours exhibit key pan-tumour biomarkers (separately for dMMR, MSI, high TMB), by applying previously published estimates for the proportion of tumours exhibiting each biomarker by cancer type/group and stage.^18^ For all of the following, high TMB is defined as ≥10 mutations/Mb as per the current Australian and US regulatory approvals.^4^ The data and methods for all analysis steps are described in detail below. All analyses were performed using Stata (version 17 and 18, Stata Corporation, College Station, TX) and R (Version 4.1.1).

### Data sources

This study obtained publicly available tabulated data on cancer incidence and survival from the Australian Institute of Health and Welfare (AIHW),^19^ and population data and projections from Australian Historical Population Statistics and Population Projections (Series B, based on medium population growth) for the period 1982 to 2042 from the Australian Bureau of Statistics (ABS).^20^^,^^21^ As historical data on stage at diagnosis are not available at the national level, we used data from the NSW Cancer Registry (NSWCR), which is the only Australian population-based cancer registry that routinely collected stage of disease information since 1972. NSW is the most populous state in Australia, representing approximately one-third of the total Australian population. The age-standardised cancer incidence rates in NSW are similar to Australia as a whole (e.g. age-standardised incidence rate of all cancers combined in 2017 were 487·1 and 492·1 per 100,000 persons, respectively).^19^ We obtained individual-level data from the Cancer Institute NSW's Enduring Cancer Data Linkage (CanDLe), which includes NSWCR data for 01/1982–12/2018 and linked NSW Registry of Births Deaths and Marriages Death Registrations (RBDM) data for 01/1985–12/2021.

### Selected cancer types/groups related to key pan-tumour biomarkers

Cancer types/groups for dedicated projections were selected based on data availability for the proportion of tumours with pan-tumour biomarkers,^18^ which resulted in 23 individual cancer types/groups (see Table S3). In particular, dedicated incidence and prevalence projections were completed for specific cancer types/groups based on two criteria: (1) from our previous review,^18^ proportion of tumours exhibiting a biomarker for that cancer type/group was >0% for at least one biomarker (dMMR, MSI, or high TMB); (2) cancer incidence data for past 5-year periods could be divided into at least three age groups with minimum 5 cases per period for each age group. Thus, we did not separately consider cancers with very low incidence (nationally <2/100,000 cases in 2017, e.g. anal, penile, and vulvar cancers). Instead, they were incorporated into the projections for “other solid cancers” as an aggregated group including 16 cancer types (see Table S3). The group of other solid cancers also included: 1) cancers with substantial changes in disease classification (e.g. cancer of unknown primary); and 2) cervical cancer, for which a new primary screening approach was introduced in 2017, leading to recent changes in incidence that are not well-suited for separate statistical projections (noting detailed projections of future cervical cancer incidence based on microsimulation modelling have been published elsewhere^22^).

### Projecting cancer incidence

#### All stages combined

We previously published statistical projections of incidence for 21 individual cancer types and all cancers combined,^23^ using tabulated data on the number of new cancer cases in Australia by sex, 5-year age group, and 5-year period (1982–2017) from the AIHW,^24^ and population data and projections by sex, 5-year age group and calendar year (1982–2042) from the ABS.^20^ A more detailed description of the methods is provided elsewhere.^23^

Building on this previous work,^23^ we further developed and validated standard age-period-cohort (APC) models or age-stratified APC models for incidence of 8 additional cancer types/groups for which there were no existing projections and which were related to dMMR/MSI/high TMB.^23^ The APC models were fitted using the *‘apcspline’* command in Stata 17, with the non-identifiability issue for APC models addressed by introducing constraints to the time effects (centering the period at the mean year and the cohort at the weighted mean birth cohort) as previously described.^25^ The model outcomes were numbers of new cancer cases, with predictors of age, period, birth cohort, and number of at-risk individuals (population estimates). The models used Poisson distribution and a log-link function, using natural cubic splines to capture non-linear period and cohort effects.^25^ We used component-plus-residual plots to check that the linearity assumption for the outcome (logarithm of the number of cases) and smoothed predictors were generally satisfied. Full details are provided in the Supplementary Information (p6), including details of age stratified APC models where required (see Table S3).

To project incidence rates beyond the observed period, future periods and cohorts were assumed to have the same effect as those for the most recently observed period and cohort, with a default damping factor used to reduce the drift by 8% each year following the last observation.^25^ The most appropriate statistical projection model for each cancer type/group was selected based on the Bayesian Information Criterion to avoid overfitting, and the selected model was evaluated through a thorough model validation process (see Table S3).^23^ As in prior work, models were validated by excluding the last 10 years of observed data from the model fitting and comparing observed to projected data for 2008–2017. We observed good fit for all new incidence projections (see Figure S1), supporting the validity of our approach. We also obtained incidence for all solid cancers combined as the sum of all 23 individual cancer types/groups. Single-year cancer incidence rates were log-linearly interpolated from 5-year period projections.

#### Advanced disease at diagnosis

Stage at diagnosis was determined by the ‘degree of spread’ variable in the NSWCR data. In this study, stage at diagnosis was grouped into advanced disease (defined as distant metastasis or lymph node involvement, in line with increasing consideration of biomarker-based treatment for the latter, see Table S2) and non-advanced disease (defined as the completement to advanced disease, i.e. disease without lymph node involvement nor distant metastases; this group was only used for interim calculations of later advanced disease after progression post-diagnosis). For each cancer type/group, the number of new cancer cases diagnosed with advanced disease at the national level was estimated by multiplying the projected number of new cancer cases by the proportion of advanced disease at diagnosis (latter based on the NSWCR data).

The NSWCR data include some individuals with “unknown” stage at diagnosis (18·5% of all solid cancers 1982–2018), based on insufficient information for the cancer registry to assign a stage. We applied a previously validated multiple imputation approach to impute a stage at diagnosis where unknown, using the Stata *‘ice’* package with multinomial logistic regression. Component-plus-residual plots were used to check the linearity assumption for the log-probability of being diagnosed with advanced stage and survival time (the only continuous predictors) were generally satisfied (see Supplementary Material p13–15 for details).^26^ We note that the estimated proportion of incident cases with advanced disease at diagnosis after imputation was similar to the estimated proportion when excluding individuals with unknown stage at diagnosis (Figure S4). To account for the uncertainty associated with imputed stage at diagnosis, all stage-specific analyses in this study were based on pooled estimates using imputed stage data and applying Rubin's rule to obtain 95% uncertainty intervals (UIs).^26^

### Estimating survival

We generated survival estimates from CanDLe data to later estimate prevalence for all stages combined and advanced disease at diagnosis (see below). We estimated all-cause survival using linked RBDM records to identify deaths from any cause. We also generated cause-specific cancer survival estimates to later project the number of individuals diagnosed with non-advanced disease that later progressed to advanced disease post-diagnosis, using NSWCR records to identify cancer deaths. CanDLe data were used as a data source for survival estimates, as stage-specific and cause-specific cancer survival estimates for each cancer type/group are not available at the national level. All survival estimates were generated for 1–5 years follow-up after initial diagnosis, by sex, 3 age groups at diagnosis (<60 years, 60–79 years and 80+ years), and 5-year diagnosis period (1989–1993 to 2014–2018).

For some cancer types/groups, the overall observed all-cause survival estimates for NSW slightly differed from the national estimates reported in the AIHW statistics^19^ (see Figure S5). Therefore, we calculated a scaling factor as the ratio between the observed national and NSW survival estimates; we set the scaling factor to 1 for cancer types/groups with no national estimates available (e.g. endocrine tumours, gallbladder and bile duct cancers, renal pelvis/ureter/urethra cancers, and other solid cancers). We then multiplied NSW survival estimates for all stages combined and for advanced disease at diagnosis by the scaling factor to derive national survival estimates (Figures S6 and S7). These survival estimates were log-linearly extrapolated forward to 2023 to account for the impact of recent improvements in treatment on survival; rates were then assumed to remain constant for 2024–2042. We acknowledge the limitations of these future survival estimates in the Discussion, and performed several sensitivity analyses for survival estimates: (1) using the unscaled NSW survival estimates; (2) assuming survival estimates remain constant for 2019–2042; (3) extrapolating survival to 2028 and then assuming survival remains constant for 2029–2042.

### Projecting prevalence of individuals with cancers

We applied a modified counting method for estimating prevalence based on incidence projections and survival estimates generated in the above analyses, separately for all stages combined and for advanced disease at diagnosis (see Supplementary Material p20 and 21).^27^ UIs for the prevalence estimates were approximated assuming independence of the incidence and survival estimates. We validated our approach by comparing the average 1- and 5-year prevalence estimates with the observed prevalence in Australia in 2014–2017 (see Figure S8).^19^^,^^24^^,^^28^^,^^29^ In general, for most cancer types/groups, our prevalence estimates were in good agreement with the observed values and the UIs of the predicted prevalence estimates captured the observed values (see Figure S8).

### Projecting prevalence of individuals with advanced disease after progression post-diagnosis

There are limited data for stage progression by cancer type for a wide range of cancers. Thus, we developed a simplified, exploratory method to estimate the prevalence of individuals with advanced disease after progression post-diagnosis, using a step-wise approach described in detail in Supplementary Material p22–25. For these calculations, we only considered individuals with disease progression within 5 years of initial diagnosis (noting the absolute risk of deaths from cancer diminished sharply over time and was generally low after 5 years^30^). Briefly, we first estimated the proportion of people diagnosed with non-advanced disease who die from cancer, as their cancer is considered to have clinically progressed to an advanced stage on the pathway from diagnosis to cancer deaths. However, not all individuals with advanced disease after progression post-diagnosis die. Thus, we back-estimated the total number of individuals with newly advanced disease after progression in a given year post-diagnosis based on the deaths and 1-year survival of individuals with advanced disease (see Figure S9). We then applied an analogous modified counting method for estimating prevalence as described above, using estimates of individuals with newly progressed disease instead of incidence estimates. As no data on survival after disease progression post-diagnosis were available, we used survival estimates for individuals with advanced disease at diagnosis as a proxy (acknowledging this limitation in the Discussion).

### Projecting prevalence of individuals with tumours exhibiting each of dMMR, MSI and high TMB

For each relevant cancer type/group, the prevalence of individuals whose tumours exhibit each pan-tumour biomarker was estimated by multiplying the projected cancer prevalence by the proportion of tumours exhibiting each biomarker in each cancer type/group and stage. These proportions were obtained from our recent scoping review and meta-analysis.^18^ Due to limited data availability, estimates for the proportion of tumours exhibiting biomarkers for advanced disease did not distinguish disease progression before versus after diagnosis (nor by treatment receipt).^18^ If the proportion of tumours with a pan-tumour biomarker was not available for advanced disease, we used the proportion across all stages if available (18, 11, and 6 of 23 cancer types/groups for dMMR, MSI and high TMB, respectively, see Table S3), or for non-advanced disease otherwise (testicular cancer for dMMR, see Table S3). The 95% UIs for prevalence estimates were estimated from the 95% UIs for cancer prevalence regardless of biomarker status and proportion of tumours with biomarker, assuming these estimates are independent. We note that the presence of these biomarkers in tumours is highly overlapping,^13^^,^^31^ thus many individuals can contribute to prevalence estimates for dMMR and MSI, as well as either of these markers and high TMB.

### Potential aggregate costs for a biomarker-based treatment

For an exploratory indicative illustration of potential aggregate costs for treatment linked to these biomarkers, we approximated the treatment target populations from 1-year prevalence, separately for those with advanced disease at diagnosis and those with advanced disease after progression post-diagnosis. The number of immunotherapy treatment doses for this illustration was based on the median treatment duration for the KEYNOTE-177 trial of pembrolizumab (16 pembrolizumab doses total, 200 mg every 3 weeks for 11.1 months)^32^; noting survival estimates within the prevalence projections were unchanged for this illustrative calculation. The cost of pembrolizumab was based on the ‘published cost’ in the PBS of AUD$78,000 per 200 mg dose^33^; the actual cost to the healthcare system is likely lower than the published cost due to a confidential Special Pricing Arrangement (see Discussion).^34^ We obtained the published total government cost for the PBS-subsidised immune checkpoint inhibitors in 2022–2023 from the PBS expenditure report^7^ (see Table S1).

### Ethics approval and consent to participate

Ethics approval to access administrative health data from the CanDLe initiative was granted by the NSW Population & Health Research Ethics Committee (HREC: 2019/ETH12584). Ethics approval was not required to use the aggregated data on cancer incidence and survival released by the AIHW.

### Role of funding source

This study was funded by Medical Research Future Fund (MRFF)—Preventive and Public Health Research Initiative—2019 Targeted Health System and Community Organization Research Grant Opportunity, as part of the Cancer-Patient Population Projections project (Cancer-PPP, grant number: MRF1200535). JS is supported by a Cancer Institute NSW Career Development Fellowship (2022/CDF1154). KC is supported by an NHMRC Investigator Grant (reference: APP1194679). The funder had no role in study design, data collection and analysis, decision to publish, preparation or submission of the manuscript.

## Results

### Projected cancer incidence

**Fig. 2:**
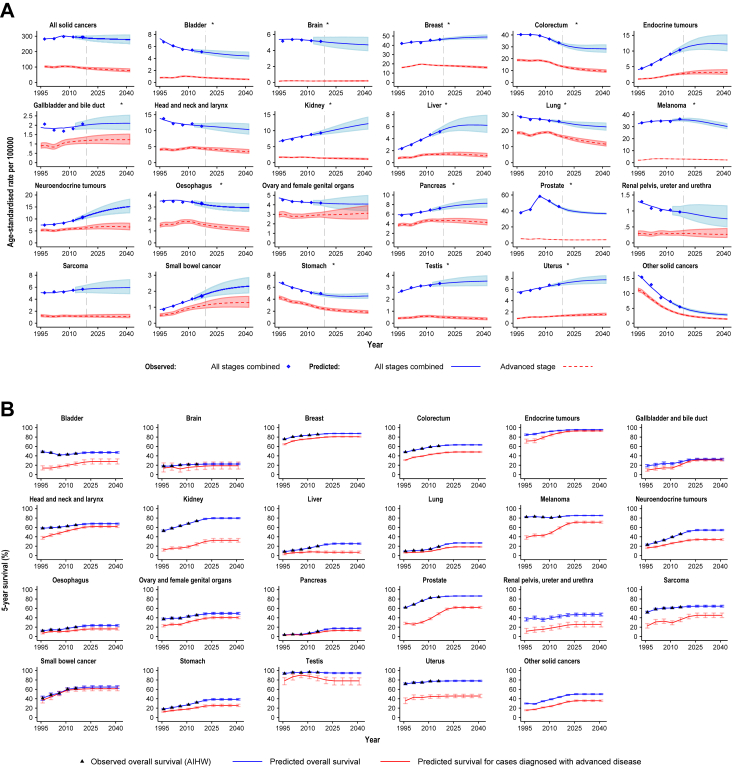
**Observed and predicted estimates of cancer incidence (A) and all-cause survival (B) for all solid cancers combined and 23 cancer types/groups associated with mismatch repair deficiency, microsatellite instability, or high tumour mutational burden (≥10 mutations/Mb) (Australia, 1995–2042)** (A) Age-standardised incidence rates. All rates were age-standardised to the Segi Word standard population. Shaded areas represent uncertainty intervals. ∗Estimates of incidence for all stages combined for these cancer types/groups as previously reported in Luo et al., 2022.^23^ Detailed projections for colorectal cancer incidence based on microsimulation modelling, which considered the implementation of the National Bowel Cancer Screening program in Australia, have been published elsewhere.^35^ (B) All-cause survival estimates. Predicted survival estimates were derived from NSW Cancer Registry data and scaled, with scaling factors to align overall survival for NSW with national estimates reported by the Australian Institute of Health and Welfare (AIHW). Survival estimates aggregated across all solid cancers are not shown as no corresponding national estimates are available and only survival estimates by cancer type/group were used in this study (see Methods and Supplementary Material p16–19). Error bars represent 95% uncertainty intervals. Observed overall survival is not shown for cancer types/groups with no estimates available from the AIHW.

Fig. 2A illustrates predicted overall age-standardised incidence rates for all solid cancers combined and by cancer types/groups, for all stages combined and advanced disease at diagnosis. The age-standardised incidence rate is expected to slightly decrease from 2018 to 2042, both for all stages combined (from 291·1 to 276·5 per 100,000 people) and advanced disease at diagnosis (from 93·0 to 77·0 per 100,000 people). The patterns vary between cancer types, with the age-standardised rates for several cancers expected to increase or be relatively stable. The number of cases with all solid cancers combined is estimated to increase from 2018 to 2041, mainly due to population growth and ageing,^23^ both for all stages combined (from 126,688 to 184,623) and for advanced disease at diagnosis (from 41,567 to 52,277), see Figure S2 and Tables S4 and S5 for details.

### Cancer survival

Survival estimates differed substantially by cancer type/group, with the observed survival for most cancer types/groups having improved over recent years (from 1999–2003 to 2014–2018), both for all stages combined and advanced disease at diagnosis (see Fig. 2B).

### Projected prevalence of individuals with cancers, regardless of biomarker status

**Fig. 3:**
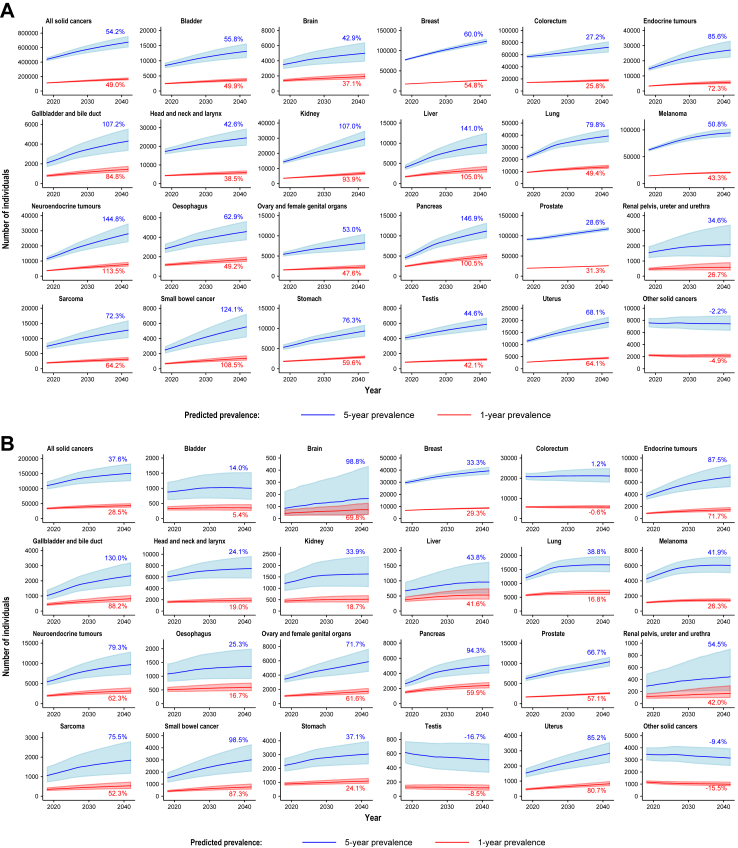

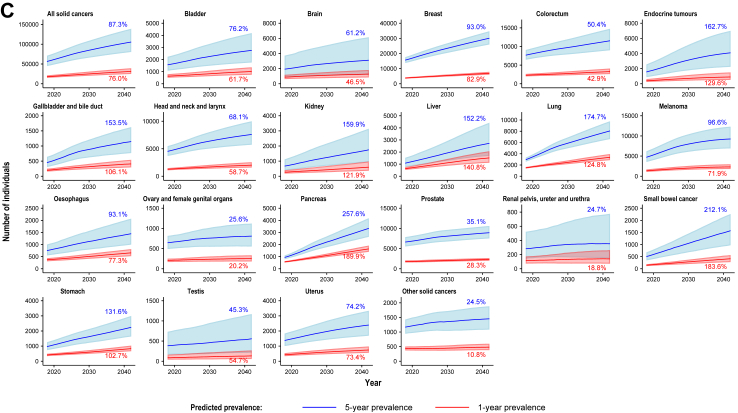
**Predicted overall prevalence of individuals, all solid cancers combined and selected cancer types/groups (regardless of biomarker status), for all stages combined (A), advanced disease at diagnosis (B) and advanced disease after progression post-diagnosis (C) (Australia, 2018–2042)** (A) All stages combined (B) Advanced disease at diagnosis (C) Advanced disease after progression post-diagnosis Shaded areas represent 95% uncertainty intervals. Percentages in the plot represent the estimated percent change, comparing estimates for 2042 to estimates for 2018. For C), prevalence of individuals diagnosed with non-advanced neuroendocrine tumours or sarcomas that later progressed to advanced disease post-diagnosis was not estimated, since data on cause-specific deaths for these cancer types were not available.

During the period 2018–2042, both 1-year and 5-year overall prevalence of individuals previously diagnosed with any solid cancer are projected to increase, by 49·0% (from 111,554 to 166,161) and 54·2% (from 438,346 to 675,722), respectively (see Fig. 3A, Table 1 and Table S7). Similarly, both 1-year and 5-year prevalence estimates for individuals with advanced disease at diagnosis are projected to increase, by 28·5% (from 33,586 to 43,144) and by 37·6% (from 109,855 to 151,199), respectively (see Fig. 3B, Table 2 and Table S8), with highest estimates for breast, colorectal, and lung cancers.

**Table 1:** Estimated 5-year prevalence of individuals with all cancers combined and selected cancer types/groups, for all stages combined (Australia, 2018–2042).

All stages combined	Total 5-year prevalence (95% uncertainty interval)		5-year prevalence (95% uncertainty interval) of individuals whose tumours exhibit a particular biomarker^a^					
	Regardless of biomarker status		Mismatch repair deficiency		Microsatellite instability		High tumour mutational burden (≥10 mut/Mb)	
	2018	2042	2018	2042	2018	2042	2018	2042
**All solid cancers combined (sum of 23 individual cancer types/groups)**								
All solid cancers	438,346 (412,662–465,877)	675,722 (602,939–760,446)	23,880 (10,453–37,310)	34,488 (14,759–54,241)	13,301 (8133–18,467)	19,323 (11,303–27,355)	71,394 (53,408–89,380)	111,982 (80,980–142,982)
**Biliary tract and gastrointestinal cancers**								
Colorectum	56,803 (53,922–59,797)	72,229 (63,650–81,994)	6652 (5227–8078)	8459 (6429–10,489)	5802 (3714–7890)	7377 (4603–10,152)	4837 (3970–5703)	6150 (4864–7436)
Gallbladder and bile duct	2082 (1668–2573)	4314 (3320–5573)	79 (28–129)	163 (56–269)	34 (20–48)	70 (40–100)	199 (63–335)	411 (125–698)
Liver	4007 (3418–4708)	9656 (7457–12,598)	NA^c^	NA^c^	58 (16–100)	138 (34–243)	57 (7–108)	137 (13–262)
Oesophagus	2813 (2384–3318)	4583 (3697–5652)	119 (48–190)	194 (76–312)	69 (31–107)	113 (49–176)	926 (705–1146)	1508 (1106–1909)
Pancreas	4534 (3982–5156)	11,193 (9431–13,248)	69 (28–110)	169 (66–272)	41 (20–63)	101 (47–156)	NA^c^	NA^c^
Small bowel	2479 (2026–2971)	5556 (4169–7292)	522 (361–682)	1169 (757–1580)	355 (125–585)	795 (260–1329)	475 (272–679)	1064 (573–1556)
Stomach	5315 (4666–6019)	9372 (7990–10,933)	464 (383–545)	818 (660–975)	454 (330–579)	801 (571–1030)	739 (558–921)	1303 (966–1641)
**Genitourinary tract cancers**								
Bladder	8428 (7559–9367)	13,127 (10,939–15,718)	369 (131–606)	574 (196–952)	247 (55–438)	384 (81–687)	3210 (1763–4656)	4999 (2648–7350)
Kidney	14,302 (13,040–15,680)	29,599 (24,949–35,056)	111 (23–199)	229 (45–413)	52 (23–80)	106 (46–167)	NA^d^	NA^d^
Prostate	91,322 (88,448–94,276)	117,408 (112,545–122,428)	5647 (591–10,702)	7259 (756–13,762)	2137 (1529–2745)	2747 (1962–3533)	3683 (2063–5304)	4735 (2647–6823)
Renal pelvis, ureter and urethra	1548 (1189–1976)	2083 (1266–3400)	68 (22–114)	92 (23–160)	46 (9–82)	61 (8–115)	590 (296–884)	794 (322–1265)
Testis	4073 (3757–4414)	5888 (5124–6747)	NA^c^	NA^c^	39 (0–77)	56 (0–111)	153 (33–273)	221 (45–396)
**Gynaecological and breast cancers**								
Breast	77,156 (74,958–79,397)	123,448 (117,028–130,178)	984 (538–1430)	1574 (857–2291)	444 (189–700)	710 (300–1120)	5592 (1233–9951)	8947 (1960–15,933)
Ovary and female genital organs	5422 (4861–6027)	8297 (6541–10,494)	310 (130–491)	475 (183–766)	243 (77–408)	371 (108–635)	630 (332–928)	964 (472–1455)
Uterus	11,406 (10,715–12,116)	19,177 (17,070–21,499)	3059 (2618–3500)	5143 (4263–6022)	2497 (1703–3291)	4198 (2808–5588)	4905 (4372–5437)	8246 (7073–9418)
**Other cancer types/groups**								
Brain	3502 (2934–4161)	5006 (3884–6433)	133 (0–268)	190 (0–386)	22 (8–36)	31 (11–52)	99 (0–198)	142 (0–285)
Endocrine tumours	14,597 (13,307–16,006)	27,091 (22,144–33,223)	99 (12–186)	183 (19–348)	94 (0–188)	174 (0–350)	848 (547–1148)	1573 (959–2186)
Head and neck and Larynx	17,207 (15,712–18,813)	24,543 (20,394–29,517)	386 (22–750)	551 (24–1077)	81 (44–118)	116 (60–171)	1475 (310–2640)	2104 (410–3797)
Lung	21,863 (20,403–23,413)	39,314 (34,293–45,031)	361 (99–623)	649 (172–1125)	94 (47–141)	168 (81–255)	6013 (3461–8565)	10,812 (6068–15,557)
Melanoma	62,649 (60,081–65,304)	94,470 (87,144–102,434)	4192 (0–8387)	6320 (0–12,664)	NA^c^	NA^c^	32,933 (30,661–35,205)	49,660 (44,924–54,396)
Neuroendocrine tumours	11,468 (10,107–12,975)	28,071 (22,539–34,899)	NA^c^	NA^c^	156 (40–271)	380 (90–670)	2842 (2441–3244)	6957 (5486–8428)
Other solid cancers^b^	7585 (6859–8375)	7416 (6275–8791)	219 (192–246)	214 (177–251)	204 (153–255)	199 (144–255)	1061 (291–1831)	1037 (272–1801)
Sarcomas	7411 (6405–8534)	12,768 (10,155–15,991)	37 (0–74)	63 (0–127)	132 (0–265)	227 (0–460)	127 (30–224)	218 (47–390)

**Note**: Estimates for projected 1-year and 2-year prevalence are available in Tables S7 and S10, respectively. All estimates are made publicly available through the Cancer-PPP dashboard [Dashboard URL see https://doi.org/10.6084/m9.figshare.28260398].

a Presence of specific biomarkers in tumours is not mutually exclusive; thus, some individuals may be included in prevalence estimates for multiple biomarkers. The underlying estimates for proportion of tumours that exhibit each pan-tumour biomarker by cancer type/group were obtained from our recent scoping review and meta-analysis, which provided estimates for all stages combined and for advanced disease where available.^18^ Due to limited data availability, we assumed that the proportions of tumours exhibiting each biomarker are the same for ‘other solid cancers' as for all solid cancers combined. The same proportions of solid tumours exhibiting dMMR/MSI/high TMB were applied to all diagnosis periods for this study.

b Projections for cancers with low incidence (<2/100,000 people) were included in an aggregated group of “other solid cancers”. This group includes cancers in the anus, cervix, eye and other central nervous system, other and ill-defined digestive organs, other and ill-defined sites, other male genital organs, other thoracic and respiratory organs, penis, peritoneum, placenta, vagina, vulva, unknown primary site, mesothelioma and non-melanoma skin cancer.

c In the published review, the reported proportion of tumours with the biomarker was 0%.^18^

d The published review did not estimate the proportion with high TMB for kidney cancer.^18^

**Table 2:** Estimated 5-year prevalence of individuals with all solid cancers combined and selected cancer types/groups, for advanced disease at diagnosis (Australia, 2018–2042).

Advanced disease at diagnosis	Total 5-year prevalence (95% uncertainty interval)		5-year prevalence (95% uncertainty interval) of individuals whose tumours exhibit a particular biomarker^a^					
	Regardless of biomarker status		Mismatch repair deficiency		Microsatellite instability		High tumour mutational burden (≥10 mut/Mb)	
	2018	2042	2018	2042	2018	2042	2018	2042
**All solid cancers combined (sum of 23 individual cancer types/groups)**								
All solid cancers	109,855 (97,298–124,115)	151,199 (124,877–183,900)	3983 (2273–5693)	5448 (2863–8035)	2484 (1170–3798)	3553 (1520–5595)	13,310 (8387–18,233)	17,893 (10,718–25,081)
**Biliary tract and gastrointestinal cancers**								
Colorectum	20,831 (19,143–22,641)	21,089 (17,806–24,939)	1432 (1107–1757)	1449 (1067–1831)	848 (516–1179)	858 (503–1213)	1774 (1437–2111)	1796 (1379–2213)
Gallbladder and bile duct	1014 (731–1401)	2332 (1673–3221)	39 (13–64)	88 (29–148)	15 (0–30)	33 (0–67)	NA^c^	NA^c^
Liver	667 (464–963)	959 (577–1632)	NA^c^	NA^c^	4 (0–7)	5 (0–11)	24 (0–48)	34 (0–70)
Oesophagus	1082 (796–1456)	1356 (904–2003)	46 (17–75)	58 (19–96)	27 (11–43)	34 (13–55)	356 (242–471)	446 (276–617)
Pancreas	2621 (2189–3136)	5092 (4008–6452)	NA^c^	NA^c^	24 (11–37)	46 (21–72)	NA^c^	NA^c^
Small bowel	1515 (1123–1969)	3007 (2033–4294)	319 (205–433)	633 (374–891)	217 (71–363)	430 (127–734)	291 (155–426)	576 (284–869)
Stomach	2223 (1780–2752)	3047 (2306–3972)	126 (80–171)	172 (105–239)	183 (34–332)	250 (42–458)	983 (351–1614)	1347 (459–2235)
**Genitourinary tract cancers**								
Bladder	877 (611–1209)	1000 (618–1530)	39 (12–66)	44 (11–77)	6 (3–10)	7 (3–11)	383 (158–608)	437 (160–714)
Kidney	1215 (880–1611)	1627 (1055–2413)	10 (2–18)	13 (2–24)	22 (0–43)	29 (0–59)	NA^d^	NA^d^
Prostate	6238 (5585–6925)	10,400 (9247–11,634)	219 (81–357)	365 (134–595)	412 (214–610)	687 (355–1018)	444 (237–650)	740 (394–1085)
Renal pelvis, ureter and urethra	288 (158–496)	445 (211–910)	13 (3–23)	20 (4–36)	2 (1–4)	3 (1–6)	126 (40–212)	195 (52–338)
Testis	617 (465–776)	514 (330–741)	NA^c^	NA^c^	6 (0–12)	5 (0–10)	24 (4–43)	20 (3–36)
**Gynaecological and breast cancers**								
Breast	29,578 (28,104–31,097)	39,424 (36,348–42,696)	377 (206–549)	503 (272–734)	55 (21–89)	73 (27–118)	2767 (2241–3294)	3688 (2952–4425)
Ovary and female genital organs	3427 (2945–3968)	5884 (4467–7706)	196 (80–312)	337 (126–547)	154 (48–259)	263 (74–453)	54 (0–108)	92 (0–188)
Uterus	1531 (1248–1843)	2836 (2219–3579)	411 (318–504)	761 (568–954)	269 (138–401)	499 (248–750)	281 (151–410)	519 (272–766)
**Other cancer types/groups**								
Brain	84 (25–224)	167 (32–435)	5 (1–8)	9 (1–17)	1 (1–2)	2 (0–3)	3 (0–6)	5 (0–11)
Endocrine tumours	3666 (3102–4279)	6872 (5211–8994)	25 (3–47)	47 (4–89)	24 (0–48)	44 (0–90)	92 (1–184)	172 (0–348)
Head and neck and Larynx	6037 (5167–7002)	7493 (5749–9681)	136 (7–265)	168 (5–332)	29 (15–42)	36 (18–53)	221 (25–417)	275 (25–524)
Lung	12,015 (10,914–13,220)	16,678 (13,984–19,846)	199 (54–343)	276 (71–480)	NA^c^	NA^c^	3480 (2364–4596)	4830 (3150–6511)
Melanoma	4253 (3666–4870)	6033 (5015–7186)	285 (0–573)	404 (0–815)	NA^c^	NA^c^	1193 (766–1621)	1693 (1064–2322)
Neuroendocrine tumours	5391 (4466–6455)	9664 (7206–12,883)	NA^c^	NA^c^	73 (18–129)	131 (29–234)	310 (82–538)	556 (133–979)
Other solid cancers^b^	3474 (2966–4039)	3148 (2497–3957)	100 (84–117)	91 (71–111)	94 (68–119)	85 (59–111)	486 (129–843)	440 (110–771)
Sarcomas	1051 (710–1498)	1845 (1151–2817)	6 (0–11)	10 (0–19)	19 (0–39)	33 (0–69)	18 (4–33)	32 (5–59)

**Note**: Estimates for projected 1-year and 2-year prevalence are available in Tables S8 and S11, respectively. All estimates are made publicly available through the Cancer-PPP dashboard [Dashboard URL see https://doi.org/10.6084/m9.figshare.28260398].

a Presence of specific biomarkers in tumours is not mutually exclusive; thus, some individuals may be included in prevalence estimates for multiple biomarkers. The underlying estimates for proportion of tumours that exhibit each pan-tumour biomarker by cancer type/group were obtained from our recent scoping review and meta-analysis, which provided estimates for all stages combined and for advanced disease where available.^18^ Due to limited data availability, we assumed that the proportions of tumours exhibiting each biomarker are the same for ‘other solid cancers' as for all solid cancers combined. The same proportions of solid tumours exhibiting dMMR/MSI/high TMB were applied to all diagnosis periods for this study.

b Projections for cancers with low incidence (<2/100,000 people) were included in an aggregated group of “other solid cancers”. This group includes cancers in the anus, cervix, eye and other central nervous system, other and ill-defined digestive organs, other and ill-defined sites, other male genital organs, other thoracic and respiratory organs, penis, peritoneum, placenta, vagina, vulva, unknown primary site, mesothelioma and non-melanoma skin cancer.

c In the published review, the reported proportion of tumours with the biomarker was 0%.^18^

d The published review did not estimate the proportion with high TMB for kidney cancer.^18^

In our exploratory analysis, the estimated proportion of individuals diagnosed with non-advanced disease that later progressed to advanced disease is projected to be relatively stable since around 2010 for most cancer types/groups (see Figure S10). During the period 2018–2042, 1-year and 5-year prevalence estimates of individuals with advanced disease after progression post-diagnosis are projected to increase by 76·0% (from 17,719 to 31,194) and 87·3% (from 56,361 to 105,582), respectively (see Fig. 3C, Table 3, Table S9).

**Table 3:** Estimated 5-year prevalence of individuals with all solid cancers combined and selected cancer types/groups, for advanced disease after progression post-diagnosis (Australia, 2018–2042).

Advanced disease after progression	Total 5-year prevalence (95% uncertainty interval)		5-year prevalence (95% uncertainty interval) of individuals whose tumours exhibit a particular biomarker^a^					
	Regardless of biomarker status		Mismatch repair deficiency		Microsatellite instability		High tumour mutational burden (≥10 mut/Mb)	
	2018	2042	2018	2042	2018	2042	2018	2042
**All solid cancers combined (sum of 23 individual cancer types/groups)**								
All solid cancers	56,361 (44,703–70,834)	105,582 (80,346–139,042)	2275 (1062–3498)	4049 (1748–6372)	1350 (625–2075)	2263 (950–3579)	7077 (4082–10,080)	13,739 (7754–19,757)
**Biliary tract and gastrointestinal cancers**								
Colorectum	7672 (6446–9061)	11,538 (8987–14,761)	528 (387–668)	793 (550–1037)	313 (183–442)	470 (261–678)	654 (500–807)	983 (707–1259)
Gallbladder and bile duct	452 (306–642)	1146 (767–1626)	17 (6–29)	44 (13–74)	7 (0–14)	17 (0–34)	NA^d^	NA^d^
Liver	1080 (767–1526)	2724 (1688–4422)	NA^d^	NA^d^	6 (0–12)	14 (0–29)	38 (0–78)	96 (0–199)
Oesophagus	750 (550–1001)	1448 (996–2069)	32 (12–52)	62 (21–102)	19 (8–30)	36 (14–58)	247 (167–327)	477 (304–649)
Pancreas	934 (782–1096)	3340 (2635–4178)	NA^d^	NA^d^	9 (4–13)	31 (14–47)	NA^d^	NA^d^
Small bowel	504 (345–677)	1573 (971–2264)	106 (64–149)	331 (180–483)	73 (22–123)	225 (59–391)	97 (49–145)	302 (136–467)
Stomach	969 (730–1255)	2244 (1634–2995)	55 (34–76)	127 (75–178)	80 (14–146)	184 (30–339)	429 (146–712)	992 (326–1658)
**Genitourinary tract cancers**								
Bladder	1565 (1074–2231)	2758 (1757–4191)	69 (20–118)	121 (32–210)	11 (5–17)	19 (7–30)	684 (278–1089)	1204 (454–1954)
Kidney	673 (387–1115)	1749 (939–3146)	6 (1–10)	14 (1–27)	12 (0–25)	31 (0–65)	NA^e^	NA^e^
Prostate	6617 (5510–7844)	8939 (7514–10,604)	232 (82–382)	313 (112–515)	437 (219–655)	590 (297–884)	471 (243–699)	636 (330–942)
Renal pelvis, ureter and urethra	283 (140–522)	353 (149–777)	13 (3–23)	16 (2–29)	2 (1–4)	3 (1–5)	124 (35–213)	155 (35–274)
Testis	384 (161–730)	558 (214–1170)	NA^d^	NA^d^	4 (0–8)	6 (0–12)	15 (0–29)	21 (0–43)
**Gynaecological and breast cancers**								
Breast	15,618 (13,695–17,591)	30,139 (25,963–34,753)	200 (106–293)	385 (202–567)	29 (11–47)	56 (20–91)	1461 (1138–1785)	2820 (2170–3470)
Ovary and female genital organs	644 (488–815)	809 (551–1121)	37 (14–60)	47 (16–77)	29 (9–50)	37 (9–64)	11 (0–21)	13 (0–27)
Uterus	1374 (1003–1831)	2393 (1687–3335)	369 (258–480)	642 (435–850)	242 (114–370)	421 (192–649)	252 (125–378)	438 (211–665)
**Other cancer types/groups**								
Brain	1931 (850–3720)	3113 (1189–6153)	100 (30–170)	161 (40–281)	13 (2–24)	20 (2–39)	55 (0–118)	88 (0–194)
Endocrine tumours	1558 (878–2546)	4093 (2239–6995)	11 (1–22)	28 (0–56)	10 (0–22)	27 (0–56)	39 (0–82)	103 (0–216)
Head and neck and Larynx	4523 (3685–5470)	7602 (5731–9997)	102 (4–199)	171 (4–337)	22 (11–32)	36 (18–55)	166 (18–314)	279 (24–533)
Lung	2933 (2581–3311)	8058 (6606–9771)	49 (13–84)	133 (34–233)	NA^d^	NA^d^	850 (569–1130)	2334 (1500–3168)
Melanoma	4703 (3533–6183)	9246 (6898–12,292)	315 (0–642)	619 (0–1262)	NA^d^	NA^d^	1320 (772–1867)	2594 (1509–3679)
Neuroendocrine tumours^b^	–	–	–	–	–	–	–	–
Other solid cancers^c^	1168 (940–1429)	1454 (1084–1883)	34 (27–41)	42 (31–54)	32 (22–41)	40 (26–53)	164 (42–286)	204 (48–360)
Sarcomas^b^	–	–	–	–	–	–	–	–

**Note**: Estimates for projected 1-year and 2-year prevalence are available in Tables S9 and S12, respectively. All estimates are made publicly available through the Cancer-PPP dashboard [Dashboard URL see https://doi.org/10.6084/m9.figshare.28260398].

a Presence of specific biomarkers in tumours is not mutually exclusive; thus, some individuals may be included in prevalence estimates for multiple biomarkers. The underlying estimates for proportion of tumours that exhibit each pan-tumour biomarker by cancer type/group were obtained from our recent scoping review and meta-analysis, which provided estimates for all stages combined and for advanced disease where available.^18^ Due to limited data availability, we assumed that the proportions of tumours exhibiting each biomarker are the same for ‘other solid cancers' as for all solid cancers combined and the same proportion of tumours exhibiting biomarkers was used for all advanced disease regardless of treatment and disease progression before or after diagnosis. The same proportions of solid tumours exhibiting dMMR/MSI/high TMB were applied to all diagnosis periods for this study.

b Prevalence of individuals diagnosed with non-advanced neuroendocrine tumours or sarcomas that later progressed to advanced disease post-diagnosis was not estimated, since data on causes of death for these cancer types were not available.

c Projections for cancers with low incidence (<2/100,000 people) were included in an aggregated group of “other solid cancers”. This group includes cancers in the anus, cervix, eye and other central nervous system, other and ill-defined digestive organs, other and ill-defined sites, other male genital organs, other thoracic and respiratory organs, penis, peritoneum, placenta, vagina, vulva, unknown primary site, mesothelioma and non-melanoma skin cancer.

d In the published review, the reported proportion of tumours with the biomarker was 0%.^18^

e The published review did not estimate the proportion with high TMB for kidney cancer.^18^

Projections of 2-year prevalence show similar trends to those for 1-year and 5-year prevalence, with all estimates provided in Tables S10–S12.

Sensitivity analyses showed that the examined alternative assumptions for future survival estimates would have a relatively small impact on overall prevalence projections (Table S13). Compared to the main estimates extrapolating survival to 2023, the projected 5-year prevalence of individuals previously diagnosed with any solid cancer was lower by 3–5% when survival was assumed to be constant from 2018, but higher by 3–5% when survival was extrapolated to 2028, or when unscaled survival from the NSW data was used as a proxy for national survival estimates (Table S13). Thus, the estimates from the main analyses only were used in the below.

### Projected prevalence of individuals with tumours exhibiting each of dMMR, MSI and high TMB (≥10 mutations/Mb)

The 5-year prevalence estimates of individuals previously diagnosed with any solid tumour exhibiting each biomarker are projected to increase over the period 2018 to 2042, for all stages combined (Table 1 and Figure S12A), advanced disease at diagnosis (Table 2 and Figure S12B) and advanced disease after progression post-diagnosis (Table 3 and Figure S12C). For all stages combined, the 5-year prevalence of individuals previously diagnosed with any solid tumour exhibiting each of dMMR, MSI and high TMB is estimated at 34,488, 19,323, and 111,982 in 2042, respectively, representing 5·1% (95% UI: 2·4–7·1%), 2·9% (95% UI: 1·9–3·6%) and 16·6% (95% UI: 13·4–18·8%) of the 5-year prevalence of all individuals diagnosed with solid cancers. We note that prevalence of specific biomarkers in tumours is not mutually exclusive, with substantial overlap expected between individuals contributing to prevalence estimates for dMMR and MSI, as well as either of these markers and high TMB.^13^^,^^31^ Considering prevalence estimates by cancer type/group, the prevalence estimates for individuals with tumours exhibiting dMMR were similar to those with tumours exhibiting MSI, with particularly high prevalence for colorectal cancer. The prevalence estimates for individuals with tumours exhibiting high TMB were substantially higher than those for dMMR or MSI, with particularly high prevalence for melanoma and lung cancer.

Similarly, for advanced disease at diagnosis, the 5-year prevalence of individuals previously diagnosed with tumours exhibiting dMMR, MSI and high TMB is estimated at 5448, 3553 and 17,893 in 2042, respectively, representing 3·6% (95% UI: 2·3–4·4%), 2·3% (95% UI: 1·2–3·0%) and 11·8% (95% UI: 8·6–13·6%) of the 5-year prevalence of all individuals with advanced disease at diagnosis.

For advanced disease after progression post-diagnosis, the corresponding 5-year prevalence estimates are 4049, 2263 and 13,739 for dMMR, MSI, and high TMB, respectively, representing 3·8% (95% UI: 2·2–4·6%), 2·1% (95% UI: 1·2–2·6%) and 13·0% (95% UI: 9·7–14·2%) of 5-year prevalence of all individuals with advanced disease after progression post-diagnosis.

Estimates for projected 1-year and 2-year prevalence are presented in Tables S7–S12, respectively. All estimates by single calendar year have been made available as part of the interactive Cancer Patient Population Projections dashboard, including customisable figures and tables [Dashboard URL see https://doi.org/10.6084/m9.figshare.28260398], and are also included as Supplementary Data.

### Potential aggregate costs for a biomarker-based treatment

The estimated 1-year prevalence of individuals diagnosed with advanced disease and tumours exhibiting MSI and high TMB in 2023 was 810 and 4864, respectively. The corresponding 1-year prevalence of individuals with advanced disease that progressed post-diagnosis was 476 and 2840, respectively. The co-occurrence of the two biomarkers depends on cancer type; a large study estimated that 97% of all tumours exhibiting MSI also exhibit high TMB (≥10 mutations/Mb).^13^ The approximate target population for a biomarker-based treatment for <1 year based on both biomarkers is thus estimated as 7743 (4864 + 2840 + 0·03∗(810 + 476)), with a 95% UI of 4723–10,769. Based on an average 16 immune checkpoint inhibitor doses per person as an example, this would amount to 123,888 (95% UI 75,568–172,304) doses total, with a cost of AUD$966·3 M (95% UI AUD$589·4M-AUD$1·34 B) based on the PBS cost for pembrolizumab (200 mg dose). We note these estimates are illustrative only, with uncertainty intervals only referring to the target population and not capturing variability in the number of doses or the uncertainty in treatment cost. Nonetheless, these estimates are compatible with the published total government cost of AUD$1·32 B for the 7 currently approved immune checkpoint inhibitors subsidised through the PBS in Australia in the 2022–23 financial year (see Table S1; key limitations of the comparison are noted in the Discussion section).^7^

## Discussion

This study presents the first long-term projections of cancer prevalence associated with key pan-tumour biomarkers (dMMR, MSI, high TMB) in Australia. We projected 1- to 5-year prevalence of individuals previously diagnosed with any solid cancer combined and 23 individual cancer types/groups in Australia to 2042, estimating overall prevalence as well as specifically the prevalence of individuals with tumours exhibiting each biomarker. The estimated prevalence of individuals with solid tumours exhibiting each key pan-tumour biomarkers is projected to increase from 2018 to 2042, for all stages combined (up to 54%), advanced disease at diagnosis (up to 38%), and advanced disease after progression post-diagnosis (up to 87%, exploratory estimates only), mainly due to population growth and ageing. The prevalence estimates for individuals whose tumours exhibit dMMR and MSI are projected to be similar, while the prevalence of individuals whose tumours exhibit high TMB (≥10 mutations/Mb) is expected to be almost 5-fold higher.

Based on our estimates aggregating prevalence across cancer types/groups and all stages, 1-year prevalence of individuals previously diagnosed with any solid tumour exhibiting each of dMMR, MSI and high TMB is estimated to account for 5·0% (95% UI 2·5–6·9), 2·9% (95% UI 1·9–3·6), and 16·6% (95% UI 13·2–19·0) of the 1-year prevalence of individuals previously diagnosed with any solid cancer. These estimated proportions are compatible with pooled estimates from other studies as reported in our previously published meta-analysis (2·9%, 2·7% and 14·0%, respectively, for all stages combined, with these published estimates contained in the above 95% UIs).^18^ We note there is expected to be very high overlap in the presence of the biomarkers: tumours exhibiting dMMR also very often exhibiting MSI and vice versa (aligned with overlapping 95% UIs for above estimated proportions), and tumours exhibiting dMMR or MSI also predominantly exhibiting high TMB. Some past studies used the terms dMMR and MSI synonymously due to their high correlation, e.g. measuring dMMR as a surrogate for MSI. More research is needed to quantify the overlap more precisely.^18^

The prevalence of individuals with tumours exhibiting each pan-tumour biomarker reflects various aspects associated with cancer care, encompassing incidence, survival, and stage distribution at diagnosis, with marked differences in proportion with each biomarker by cancer type/stage. Accordingly, we estimate that the prevalence of individuals diagnosed with solid tumours exhibiting each pan-tumour biomarker is particularly high for a few cancer types. For example, colorectal cancer currently accounts for 14% of the prevalence for individuals with advanced disease at diagnosis, and up to 27% for individuals with advanced disease at diagnosis whose tumours exhibit dMMR/MSI. As another example, endometrial and small bowel cancers together are rarer, accounting for 3·6% of the prevalence for individuals with advanced disease at diagnosis; however, they account for up to 24% of the prevalence for individuals with advanced disease at diagnosis whose tumours exhibit dMMR/MSI, which are common biomarkers for these cancers. Lung cancer is another cancer type with a substantial contribution to prevalence estimates, accounting for up to 27% of prevalence for individuals with advanced disease at diagnosis whose tumours exhibit high TMB. Melanoma is a common cancer with high TMB as a common biomarker; however, melanoma is predominantly diagnosed very early and accounts for only 9% of the prevalence for individuals with advanced disease at diagnosis whose tumours exhibit high TMB (versus 44% of the corresponding prevalence for all stages combined).

Tumour-agnostic drugs are expected to incur substantial costs to the healthcare system, and better understanding the potential patient populations is vital for evidence-based health technology assessments and subsequent allocation of resources to deliver effective and efficient care. We provided high-level prevalence estimates that can be leveraged for more in-depth analyses of patient populations. Per our exploratory indicative illustration of the potential budget impacts for immune checkpoint inhibitors, even 7743 individuals receiving 16 doses of immune checkpoint inhibitors each could amount to over AUD$966 M based on the PBS-listed treatment costs 2022–23. Such a cost would be equivalent to >5% of the published total PBS expenditure in 2022–23 (estimated AUD$16·9 billion),^7^ compatible with total published government costs for PBS-covered immune checkpoint inhibitors in that year (noting published estimates are based on aggregate data for several immune checkpoint inhibitors, which in that period were subsidised for different cancer types/groups with differing complex eligibility criteria). These high costs re-enforce the need for prevalence projections related to these pan-tumour biomarkers to support health system planning. However, it should be noted that the actual costs associated with immune checkpoint inhibitors to the Australian government might be substantially different from these estimates. In particular, the Australian government may enter into confidential Special Pricing Arrangements with a sponsor for supplying the medicine, with actual per-dose costs lower than the published costs.^34^ Considering future costs, patents for several immune checkpoint inhibitors are expected to expire in the next 3–5 years (e.g. for pembrolizumab (Keytruda) in 2028).^36^ An introduction of relevant generic agents can be expected to reduce per-dose health system costs for some immune checkpoint inhibitor treatments in the future. We also note that there are several limitations of the comparison between estimated and published total costs. First, the estimates are illustrative only and do not include price differences between different immune checkpoint inhibitors, as drug-specific estimates are beyond the scope of this study. Second, the patient population estimates are subject to substantial uncertainty as per the prevalence estimates and limited information on concordance between biomarkers. Third, our projections include individuals with locally advanced cancers and lymph node involvement, not all of whom would currently be eligible for targeted treatment; we also note current treatment eligibility criteria are complex and can include prior treatment or consideration of other biomarkers, which are beyond the scope of this study. Nonetheless, the exploratory, indicative estimates were compatible with published data for 2022–23. In fact, the actual published costs of immune checkpoint inhibitors subsidised through the PBS for a range of tumour types were close to the upper limits of the 95% UIs in predictions, suggesting patients currently receiving treatment may receive more than 16 doses on average. Future in-depth analyses would be required to reflect complex treatment eligibility criteria, different treatment duration, and costs for different treatments.

This study has some limitations that should be considered when interpreting the broader prevalence results. First, all long-term projections are subject to inherent uncertainty, and reflective of the incorporated data and methods used to project future changes. This includes potential model misspecification bias in analyses of cancer incidence and survival. To reflect likely future changes in incidence and survival, we explicitly extrapolated current trends, assuming that the cancer control gains made in the past will continue into the future. However, the projections cannot reflect future changes that disrupt existing trends in population-level cancer risk (e.g. due to new risk factors or larger changes in immigration), the effects of future new early detection technologies, or new treatments. In particular, our projections do not reflect effects of the very recent full rollout of the National Bowel Cancer Screening Program, nor the new National Lung Cancer Screening Program (planned to commence by June 2025). Detailed projections for colorectal cancer incidence based on microsimulation modelling, taking into account detailed data on screening utilisation, have been published previously.^35^ Nonetheless, the explicit specification of assumptions regarding future trends is a notable strength of this study, enabling projections that can be easily updated with emergence of new data in the future. Another limitation is that we do not have national-level data on stage-specific incidence and survival, so the main analysis uses NSW cancer stage distribution at diagnosis and survival estimates as a proxy for Australian estimates (noting that NSW accounts for one-third of the Australian population, and cancer incidence and mortality rates for most cancer types are almost identical to the national rates).^19^ In addition, the proportions of solid tumours exhibiting dMMR/MSI/high TMB are taken from our published estimates, which are largely based on clinical studies that are likely not representative of the general population. We also applied the same proportions across all periods for this study (without changes across time), with the simplifying assumption that these proportions apply similarly for advanced disease at diagnosis and after progression post-diagnosis (noting this is unlikely to be completely accurate due to treatment effects). Again, our methodology allows for updates of projections in the future, once more detailed and representative data on the proportions of tumours with these biomarkers become available. Finally, we did not consider differences in survival by biomarker status (which could occur even in the absence of targeted treatment, e.g. due to enhanced antitumour immune environment for tumours exhibiting dMMR or MSI) and associated treatment delivery (e.g. increased survival associated with receipt of immune checkpoint inhibitors). Future work could extend our approach to include in-depth modelling of treatment patterns and survival by treatment approach, which is beyond the scope of this study.

There are several other challenges in estimating the budget impacts of tumour-agnostic drugs that would need to be addressed by future studies. The aggregate number of cancer patients whose tumours exhibit any of the pan-tumour biomarkers would require information on the concordance of all three biomarkers, based on measurements of these biomarkers in the same tumours. Such information was not available for this study. As discussed above, further review and updates of projections will also be needed to reflect future influence of new prevention or screening programs, and other changes in incidence, stage distribution, and survival. In particular, treatment criteria will likely change over the projection period in this study, with likely advances in more precise targeting of treatment (e.g. based on studies of large patient cohorts and new analytic technologies such as artificial intelligence). Thus, ongoing efforts will be required to adjust and refine prevalence projections in the future accordingly. Importantly, with rigorous methodology and analytical workflows, our study provides the foundation for such future efforts.

This study also has many strengths. We used long-term data on cancer at national level and for NSW, which are known to be of high quality.^19^ We also integrated extensive data for stage distribution at diagnosis, stage-specific survival, and proportions of tumours exhibiting the biomarkers. We applied a systematic approach to project cancer incidence and prevalence of individuals with cancers in the long term, taking into account detailed data on tobacco consumption, cancer screening, and prostate-specific antigen testing (Table S3). For cancer types without explicitly modelled factors, age, period and cohort effects were used to capture the effect of factors associated with cancer incidence at a population level.^23^ Moreover, we have conducted comprehensive validations on the modelling approaches for incidence and prevalence using observed data, which demonstrated the reliability of our projections. The comprehensive modelling approach on cancer prevalence with key biomarkers integrating multiple large-scale analyses can also be used elsewhere. Furthermore, all estimates from this study are also made publicly available through the interactive Cancer Patient Population Projections (Cancer-PPP) dashboard [Dashboard URL see https://doi.org/10.6084/m9.figshare.28260398] and included as Supplementary Data. These detailed results will support demand and budget estimates for existing and future targeted cancer therapies.

In conclusion, this study presents the first long-term projections for prevalence of individuals with cancers associated with key pan-tumour biomarkers in Australia. These detailed projections can inform health technology assessments and health policy related to biomarker-based medicines, supporting future planning in a key area of clinical practice. We established a systematic, comprehensive approach to estimate prevalence of individuals with cancers associated with key biomarkers, which can be readily extended to other countries, biomarkers, and areas of precision medicine.

## Contributors

JS conceived the study. JS, YJK, QL and KC designed the study. QL performed statistical projections for cancer incidence and imputed missing data for cancer stage. YJK calculated stage-specific cancer survival estimates. QL estimated the prevalence of all individuals with advanced disease after progression post-diagnosis, YJK performed all other analyses of cancer prevalence. AK led the design of the graphical abstract. JW led the design and implementation of the dashboard. YJK, QL and JS drafted the manuscript. All authors contributed to interpretation of the results and critical review of the manuscript, approving the final version for publication.

## Data sharing statement

The tabulated national data on cancer incidence, survival and prevalence are available from the Australian Institute of Health and Welfare at https://www.aihw.gov.au/.

This study also uses data from the CanDLe Initiative, with on-provision by authors not permitted by the relevant data custodians. However, the data are available for approved research projects through CanDLe–data access enquiries can be made to the CanDLe (see https://www.cancer.nsw.gov.au/research-and-data/cancer-data-and-statistics/data-available-on-request for details). Other researchers would be able to access these data using the same process followed by the authors.

Data supporting the findings of this study are available within the article and the Supplementary Material and Supplementary Data. All projected 1–5-year prevalence estimates by single calendar year (2018–2042) are made publicly available through the Cancer-PPP dashboard [Dashboard URL see https://doi.org/10.6084/m9.figshare.28260398].

## Declaration of interests

Professor Karen Canfell is co-principal investigator of an investigator-initiated trial of cervical screening, Compass, run by the Australian Centre for Prevention of Cervical Cancer (ACPCC), which is a government-funded not-for-profit charity; the ACPCC has received equipment and a funding contribution from Roche Molecular Diagnostics, and operational support from the Australian Government. KC is also co-principal investigator on a major investigator-initiated implementation programme Elimination Partnership in Cervical Cancer (EPICC) which receives support from the Australian government and Minderoo Foundation and equipment donations from Cepheid.

Professor John Zalcberg has received speaker fees, or travel/accommodation/expenses payments from BMS, MSD Oncology, ICON Group, and Praxis. JZ is a member of the board of directors for ICON and Praxis, and an advisor or consultant for Alloplex Biotherapeutics, Avance Clinical, BioNTech SE, BioIntelect, Lipotek, Deciphera, Duo Oncology, FivePHusion, Genorbio, MSD, Oncology Republic, RevMed, Taiho Oncology, Takeda, and 1Global. JZ has financial interests in Amarin, Biomarin, CSL, Frequency Therapeutics, Gilead, Korro, Moderna Therapeutics, Nonavax, Opthtea, Orphazyme, UniQure, Servier, STA, Taiho, BMS, RevMed, ICON, and Praxis. JZ's institution has received funding from Astellas Pharma, AstraZeneca, BMS, Eisai, Ipsen, IQvia, Medtronic, MSD Oncology, Mylan, Servier, Taiho Oncology, and Pfizer.

YJK, QL, JW, AK, JC and JS declare that they have no conflict of interest.
