# Supplementary material for: Informing health system planning for biomarker-based treatment: statistical prevalence projections for solid cancers with key pan-tumour biomarkers (dMMR, MSI, high TMB) in Australia to 2042
Source: Lancet Reg Health West Pac. 2025 Apr 4;57:101537. doi: 10.1016/j.lanwpc.2025.101537 (PMC12002892; doi:10.1016/j.lanwpc.2025.101537)
Supplement: Appendix [file mmc2.pdf]

Informing health system planning for biomarker-based treatment: statistical prevalence projections for solid cancers with key pan-tumour biomarkers (dMMR, MSI, high TMB) in Australia to 2042

Table of contents

List of tables 1

List of figures 2

1. Current status of immune checkpoint inhibitors: key relevant approvals, indications, and government cost for the subsidised treatment (as of December 2023) 3

2. Cancer incidence projection and validation 6

2.1 Detailed incidence projection models and assumptions 6

3. Multiple imputation for unknown stage at diagnosis 13

4. Survival analyses 16

5. Prevalence projections 20

6. Estimating prevalence of advanced disease after progression post-diagnosis 22

7. Additional results for projected cancer prevalence 26

8. Sensitivity analyses 35

9. References 36

List of tables

Table S1. Published government cost for the PBS-subsidised immune checkpoint inhibitors approved by Therapeutic Goods Administration in Australia, 2022-23 financial year 3

Table S2. High-level overview of TGA approvals and NCCN treatment guidelines of immune checkpoint inhibitors for solid cancers 4

Table S3. ICD-10 codes and incidence projection methods for selected cancer types/groups, mapped against availability of data on proportions of tumours exhibiting each biomarker 7

Table S4. Observed and projected age-standardised incidence rates and numbers of new cases for all solid cancers combined and selected cancer types/groups, for all stages combined (Australia, 2018-2042) 11

Table S5. Predicted age-standardised incidence rates and numbers of new cases for all solid cancers combined and selected cancer types/groups, for advanced disease at diagnosis (Australia, 2018-2042) 12

Table S6. Median survival (in years) for individuals diagnosed with advanced disease and who later die from cancer 23

Table S7. Estimated 1-year prevalence of individuals with all solid cancers combined and selected cancer types/groups, for all stages combined (Australia, 2018-2042) 26

Table S8. Estimated 1-year prevalence of individuals with all solid cancers combined and selected cancer types/groups, for advanced disease at diagnosis (Australia, 2018-2042) 27

Table S9. Estimated 1-year prevalence of individuals with all solid cancers combined and selected cancer types/groups, for advanced disease after progression post-diagnosis (Australia, 2018-2042) 28

Table S10. Estimated 2-year prevalence of individuals with all solid cancers combined and selected cancer types/groups, for all stages combined (Australia, 2018-2042) 29

Table S11. Estimated 2-year prevalence of individuals with all solid cancers combined and selected cancer types/groups, for advanced disease at diagnosis (Australia, 2018-2042) 30

Table S12. Estimated 2-year prevalence of individuals with all solid cancers combined and selected cancer types/groups, for advanced disease after progression post-diagnosis (Australia, 2018-2042) 31

Table S13. Sensitivity analyses using alternative assumptions for future survival, showing resulting projected 5-year prevalence of individuals with all solid cancers combined, regardless of biomarker status (Australia, 2042) 35

List of figures

Figure S1. 10-year validation of cancer incidence projection methods 9

Figure S2. Observed and predicted age-standardised cancer incidence rates by sex (Australia, 1982-2042) 10

Figure S3. Comparison of summary stage distribution for NSW after imputation with US SEER statistics, both for diagnoses 2010-2016 14

Figure S4. Proportion of cases with advanced disease at diagnosis: estimates based directly on registry data for all cases (with "unknown" stage as a separate category), complete-case analysis (excluding records with "unknown" stage), and after multiple imputation. 15

Figure S5. Comparison of 5-year overall survival estimates by age at diagnosis in males and females combined (Australia, 1990-2018): predicted survival (using the NSWCR data) and the observed national estimates (reported by the AIHW) 17

Figure S6. Predicted all-cause survival estimates by age group in males and females combined (Australia, 1995-2042) 18

Figure S7. Predicted all-cause survival estimates for individuals diagnosed with advanced disease by age group in males and females combined (Australia, 1995-2042) 19

Figure S8. Comparison of the average 1- and 5-year prevalence in 2014-2017: predicted prevalence in this study and observed prevalence (reported by the AIHW) 21

Figure S9. Disease progression: from non-advanced stage at diagnosis to advanced disease post-diagnosis 22

Figure S10. Estimated proportion of cancer cases diagnosed with non-advanced disease that progressed to advanced disease post-diagnosis, for all ages combined and by years since the initial diagnosis (Australia, 1992-2018) 24

Figure S11. Estimated proportion of individuals diagnosed with non-advanced disease that progressed to advanced disease post-diagnosis, by age at initial diagnosis and years since the initial diagnosis, based on individuals diagnosed 2009-2013 25

Figure S12. Projected 5-year prevalence of individuals with tumours exhibiting key biomarkers, for relevant cancer types/groups and all stages combined (A), advanced disease at diagnosis (B) and advanced disease after progression post-diagnosis (C) (Australia, 2018-2042) 32

1. Current status of immune checkpoint inhibitors: key relevant approvals, indications, and government cost for the subsidised treatment (as of December 2023)

Table S1. Published government cost for the PBS-subsidised immune checkpoint inhibitors approved by Therapeutic Goods Administration in Australia, 2022-23 financial year

Class	Active ingredient (product name)	Government cost listed in PBS expenditure report (A$)[a]	Average published government cost per prescription (A$)[b]
PD-1 inhibitor	Pembrolizumab (Keytruda)	$447,023,724	$8,488
	Nivolumab (Opdivo)	$411,450,540	$7,339
	Cemiplimab (Libtayo)	$59,595,765	$7,465
PD-L1 inhibitor	Atezolizumab (Tecentriq)	$136,087,685	$7,416
	Durvalumab (Imfinzi)	$71,410,842	$8,027
	Avelumab (Bavencio)	$43,919,091	$6,083
CTLA-4 inhibitor	Ipilimumab (Yervoy/Winglore)	$151,019,563	$20,236
	Tremelimumab (Imjudo)[c]	-	-
**Total**	**Total**	**$1,320,507,210**	**$8,324**

PBS – Pharmaceutical Benefits Scheme.

[a] We note the government cost does not include out-of-pocket patient contributions. As of 1 August 2024, different immune checkpoint inhibitors are subsidised through the PBS for different tumour types, and the specific criteria for the PBS listing vary (e.g. treatment setting, treatment duration, prior treatment, patient performance status, biomarkers and concomitant treatment).[1]

[b] The average government cost is calculated by dividing the total government expenditure by the number of PBS-subsidised prescriptions, which may include variations of the same product with different dosing regimens. For example, for pembrolizumab, a 200 mg dosage has a published government cost of approximately $7,800 and would typically be administered every three weeks. A 400 mg dosage has a published government cost of around $15,000 and would be administered every six weeks, leading to similar but not identical total costs.[2 3]

[c] Tremelimumab was approved in Dec. 2023 and was thus not included in PBS-subsidised medicines in the 2022-23 financial year.

Source: Pharmaceutical Benefits Scheme (PBS) Expenditure & Prescriptions Report 1 July 2022 to 30 June 2023.[4]

Table S2. High-level overview of TGA approvals and NCCN treatment guidelines of immune checkpoint inhibitors for solid cancers

	TGA approval [a] for unresectable or metastatic tumours			TGA approval [a] for recurrent or refractory tumours			TGA approval [a] or NCCN treatment guidelines [b] (if not approved by TGA) for stage 3 or locally advanced tumours		
Cancer	Exhibiting dMMR, MSI, high TMB[c]	With/without other biomarkers	Regardless of biomarker status	Exhibiting dMMR, MSI, high TMB	With/without other biomarkers	Regardless of biomarker status	Exhibiting dMMR, MSI, high TMB	With/without other biomarkers	Regardless of biomarker status
Biliary tract cancer	Approved by TGA		Approved by TGA						Approved by TGA
Breast cancer	Approved by TGA	Approved by TGA (PD-L1: CPS ≥ 10)			Approved by TGA (PD-L1: CPS ≥ 10)				Approved by TGA (stage 2/3 TNBC)
Cervical cancer	Approved by TGA	Approved by TGA			Approved by TGA				
Cutaneous SCC	Approved by TGA		Approved by TGA			Approved by TGA			Approved by TGA
Colorectal cancer	Approved by TGA						*Preferred treatment based on NCCN treatment guidelines*		
Endometrial carcinoma	Approved by TGA	Approved by TGA (not exhibiting dMMR/MSI)							*Preferred treatment based on NCCN treatment guidelines*
Gastric cancer	Approved by TGA	Approved by TGA (HER2 -ve)					*Preferred treatment based on NCCN treatment guidelines*	*Preferred treatment based on NCCN treatment guidelines (PD-L1: CPS ≥ 1)*	
Gastro-oesophageal junction Cancer	Approved by TGA	Approved by TGA (HER2 -ve)					*Preferred treatment based on NCCN treatment guidelines*	*Approved by TGA (HER2 -ve); preferred treatment based on NCCN treatment guidelines (PD-L1: CPS ≥ 1)*	
Head and neck SCC	Approved by TGA	Approved by TGA (PD-L1: CPS ≥ 1)			Approved by TGA (PD-L1: CPS ≥ 1)				
Hepatocellular carcinoma	Approved by TGA		Approved by TGA			Approved by TGA			
Malignant pleural mesothelioma	Approved by TGA		Approved by TGA						*Preferred treatment based on NCCN treatment guidelines*
Melanoma	Approved by TGA		Approved by TGA						Approved by TGA
Merkel Cell Carcinoma	Approved by TGA		Approved by TGA						*Preferred treatment based on NCCN treatment guidelines*
Neuroendocrine tumours	Approved by TGA						*Useful treatment based on NCCN treatment guidelines*		
Non-small cell lung cancer	Approved by TGA	Approved by TGA (EGFR or ALK -ve; PD-L1: TPS ≥ 1%)	Approved by TGA		Approved by TGA (EGFR or ALK -ve)	Approved by TGA			Approved by TGA
Oesophageal cancer	Approved by TGA	Approved by TGA (HER2 -ve)	Approved by TGA		Approved by TGA (HER2 -ve)	Approved by TGA			Approved by TGA
Pancreatic adenocarcinoma	Approved by TGA						*Preferred/useful treatment based on NCCN treatment guidelines*		

Cancer	TGA approval [a] for unresectable or metastatic tumours			TGA approval [a] for recurrent or refractory tumours			TGA approval [a] or NCCN treatment guidelines [b] (if not approved by TGA) for stage 3 or locally advanced tumours		
	**Exhibiting dMMR, MSI, high TMB[c]**	**With/without other biomarkers**	**Regardless of biomarker status**	**Exhibiting dMMR, MSI, high TMB**	**With/without other biomarkers**	**Regardless of biomarker status**	**Exhibiting dMMR, MSI, high TMB**	**With/without other biomarkers**	**Regardless of biomarker status**
Renal cell carcinoma	Approved by TGA		Approved by TGA			Approved by TGA (intermediate-high risk of recurrence)			*Preferred treatment based on NCCN treatment guidelines (stage 2/3 clear cell histology)*
Small bower adenocarcinoma	Approved by TGA						*Useful treatment based on NCCN treatment guidelines*		
Small cell lung cancer	Approved by TGA		Approved by TGA (extensive stage)						
Urothelial carcinoma	Approved by TGA		Approved by TGA			Approved by TGA (high-risk of recurrence)			Approved by TGA
Other solid cancers	Approved by TGA								

TGA – Therapeutic Goods Administration (Australia); NCCN – National Comprehensive Cancer Network; dMMR – mismatch repair deficiency; MSI – microsatellite instability; high TMB – high tumour mutational burden (≥10 mutations/Mb); TNBC – triple negative breast cancer; SCC – squamous cell carcinoma; CPS – combined positive score; TPS – tumour proportion score; HER2 -ve – HER2 overexpression negative; EGFR, or ALK -ve – with no EGFR or ALK genomic tumour aberrations;.

[a] Source: Australian Public Assessment Report for Keytruda,[5] opdivo,[6] nivolumab and ipilimumab,[7] atezolizumab,[8] cemiplimab,[9] avelumab,[10] Imfinzi,[11] and imjudo.[12]

[b] Source: NCCN guidelines for treatment by cancer type: colon cancer,[13] rectal cancer,[14] uterine neoplasms,[15] gastric cancer,[16] mesothelioma: pleural,[17] merkel cell carcinoma,[18] pancreatic adenocarcinoma,[19] kidney cancer,[20] small bowel adenocarcinoma,[21] and neuroendocrine and adrenal tumors.[22]

[c] Approved for adult and paediatric patients with unresectable or metastatic solid tumours exhibiting dMMR, MSI, high TMB (≥10 mutations/Mb) that have progressed following prior treatment and who have no satisfactory alternative treatment options.

2. Cancer incidence projection and validation

2.1 Detailed incidence projection models and assumptions

Please note parts of this section detailing cancer incidence projections were reproduced from the supplementary content of the following publication:[23]

> Luo Q, O'Connell DL, Yu XQ, Kahn C, Caruana M, Pesola F, et al. Cancer incidence and mortality in Australia from 2020 to 2044 and an exploratory analysis of the potential effect of treatment delays during the COVID-19 pandemic: a statistical modelling study. The Lancet Public health. 2022;7(6):e537-e48.

Age-period-cohort (APC) models

To project cancer incidence for cancers of (1) renal pelvis and ureter, (2) ovary and female genital organs, (3) small bowel, (4) head and neck and larynx, we used APC models including age, period and cohort components within the framework of a GLM with Poisson distribution. The APC models were fitted by the '*apcspline'* command in Stata 17 with natural cubic splines for smoothing.[24] Briefly, we compared a number of APC models with different numbers of knots for the age, period and cohort effects to identify the one with the lowest Bayesian information criterion (BIC).[23] The APC model with the log-link function can be expressed as:

$$lnD_{ij} = lnN_{ij} + \alpha Age_i + \beta Period_j + \gamma Cohort_k$$

where D_{ij} denotes the number of new cancer cases for the ith age group during the jth calendar period; N_{ij} denotes the number at risk in the population for the ith age group during the jth calendar period; α is the coefficient of the age component for age group i; β is the non-linear coefficient of the period component for period j, and γ is the non-linear coefficient of the cohort component for birth cohort k. To project mortality rates beyond the observed period, future periods and cohorts were assumed to have the same effect as those for the most recent observed period and cohort. As these historical trends will not continue indefinitely, the default setting for the damping factor (equal to 0.92) was used, so that the drift was reduced by 8% for each year following the last observation.[34] In this study, the log link model offered better model fit compared to the power function, therefore estimates based on the log link model were presented.

Age-stratified APC models

For those cancer types/groups where visual inspection of model validation results for APC models as described above suggested poor fit, we also fitted age-stratified APC models, splitting the data into two large age groups. The performance of these age-stratified APC models was then compared with the performance of the standard APC models. This approach was used to project cancer incidence for (1) endometrial cancer, (2) neuroendocrine tumours, (3) sarcoma and (4) other solid cancers. APC models were fitted separately for two age groups: (i) age <50 years, and (ii) age 50 years and over. To project cancer incidence for endocrine tumours, APC models were fitted separately for two age groups: (i) age<25 years, and (ii) age 25 years and over. This method aims to capture more complex patterns (e.g. diverging trends) in age-specific incidence rates between younger and older age groups.[23] For all five cancer types/groups where age-stratified models were fitted, the model validation suggested good fit of the age-stratified models and an improvement compared to the standard APC models; thus, we used the age-stratified models for the analyses presented in the main manuscript.

Table S3. ICD-10 codes and incidence projection methods for selected cancer types/groups, mapped against availability of data on proportions of tumours exhibiting each biomarker

Cancer type/group	ICD-10 code	Incidence projection method	Data availability for proportions of tumours exhibiting each biomarker [a] [25]								
			Mismatch repair deficiency			Microsatellite instability			High TMB (≥10 mutations/Mb)		
			All stages combined	Non-advanced disease	Advanced disease	All stages combined	Non-advanced disease	Advanced disease	All stages combined	Non-advanced disease	Advanced disease
All solid cancers combined											
All solid cancers combined	C00-97, D45-46, D47.1, D47.3-5 excluding all blood cancers (C81–C96, D45, D46, D47.1, D47.3–D47.5)	Number of new cases estimated by summing the number of new cases for 22 selected cancer types/groups and the remaining cancer types as an 'other solid cancers' group. Projections available from 1992, as data required for prostate and breast cancer projections were only available from the 1990s (see below).	√			√			√		
Biliary tract and gastrointestinal cancers											
Colorectum*	C18-C20	Age-stratified APC model (separate models for age <50 years and age 50+)	√	√	√	√	√	√	√		
Gallbladder and bile duct*	C23-C24	APC model	√			√		√	√		√
Liver*	C22	AC model	√			√		√	√		√
Oesophagus*	C15	AP model for males, AC model for females	√			√			√		
Pancreas*	C25	AP model for males, APC model for females	√		√	√			√		
Small bowel	C17	APC model	√	√		√	√		√		
Stomach*	C16	APC model	√	√	√	√	√	√	√		√
Genitourinary tract cancers											
Bladder*	C67	APC model	√			√		√	√	√	√
Kidney*	C64	APC model	√			√		√			
Prostate*	C61	GLM: includes age, period and PSA testing rate as a covariate. Projections available from 1992, as data on PSA testing were only available from the 1990s.	√		√	√		√	√		√
Renal pelvis, ureter and urethra	C65-C66, C68	APC model	√			√		√	√	√	√
Testis*	C62	APC model		√				√			√
Gynaecological and breast cancers											
Breast*	C50	APC model with screening participation rate as a covariate. Projections available from 1992, as screening participation is only available from the 1990s.	√			√		√	√		√
Ovary and female genital organs	C56-C57	APC model	√	√		√			√		√
Uterus*	C54-C55	APC model									
Endometrium [b]	*C54.1*	*APC model*	√	√		√	√	√	√		√
Other cancer types/groups											
Brain*	C71	AC model	√		√	√	√	√	√		
Endocrine tumours	C73-75 (excluding C75.1–C75.3)	Age-stratified APC model (separate models for age <25 and age 25+ years)	√			√			√		√
Thyroid *, [b]	*C73*	*APC model*	√			√					√
Head and neck and larynx	C00-C14, C30-C31, C32	APC model	√			√			√		√
Head and neck *	*C00-C14, C30-C31*	*APC model*	√			√			√		√
*Larynx**	*C32*	*APC model*									

Cancer type/group	ICD-10 code	Incidence projection method	Data availability for proportions of tumours exhibiting each biomarker [a] [25]								
			Mismatch repair deficiency			Microsatellite instability			High TMB (≥10 mutations/Mb)		
			All stages combined	Non-advanced disease	Advanced disease	All stages combined	Non-advanced disease	Advanced disease	All stages combined	Non-advanced disease	Advanced disease
Lung*	C33-C34	APC model with cigarette smoking exposure as a covariate	√			√		√	√	√	√
Melanoma*	C43	APC model	√			√			√		√
Neuroendocrine tumours[c]	Histology codes as specified by the AIHW[26]	Age-stratified APC model (separate models for age <50 years and age 50+ years)	√			√			√		√
Sarcoma	C40-C41, C49 plus other topography codes as specified by the AIHW[26]	Age-stratified APC model (separate models for age <50 years and age 50+ years)	√			√	√		√		
Other solid cancers [d]	All solid cancers excluding the 22 cancer types/groups with dedicated projections	Age-stratified APC model (separate models for age <50 years and age 50+ years)									

TMB – tumour mutational burden; APC: age-period-cohort. AP: age-period. AC: age-cohort. GLM: generalised linear model. PSA: prostate specific antigen.

* Incidence projections for these cancer types/groups were reported in Luo et al 2022.[23]

[a] Advanced disease is defined as tumours with lymph node involvement or distant metastases, regardless of treatment and disease progression before or after diagnosis.

[b] Separate projections were performed for these main cancer sub-types as they are of particular relevance to the biomarkers of interest. For the main analyses, these estimates were not considered separately from the relevant aggregated cancer groups.

[c] Histology codes 8013, 8040–8045, 8150–8156, 8158, 8240–8249, 8345–8347, 8680–8683, 8690–8693, 8700, 9091 for all topography codes or 8510 for topography C73.

[d] Projections for cancers with low incidence (<2/100,000 people) were included into an aggregated group of "other solid cancers". This group includes cancers in the anus, cervix, eye and other central nervous system, other and ill-defined digestive organs, other and ill-defined sites, other male genital organs, other thoracic and respiratory organs, penis, peritoneum, placenta, vagina, vulva, unknown primary site, mesothelioma and non-melanoma skin cancer.

Figure S1. 10-year validation of cancer incidence projection methods

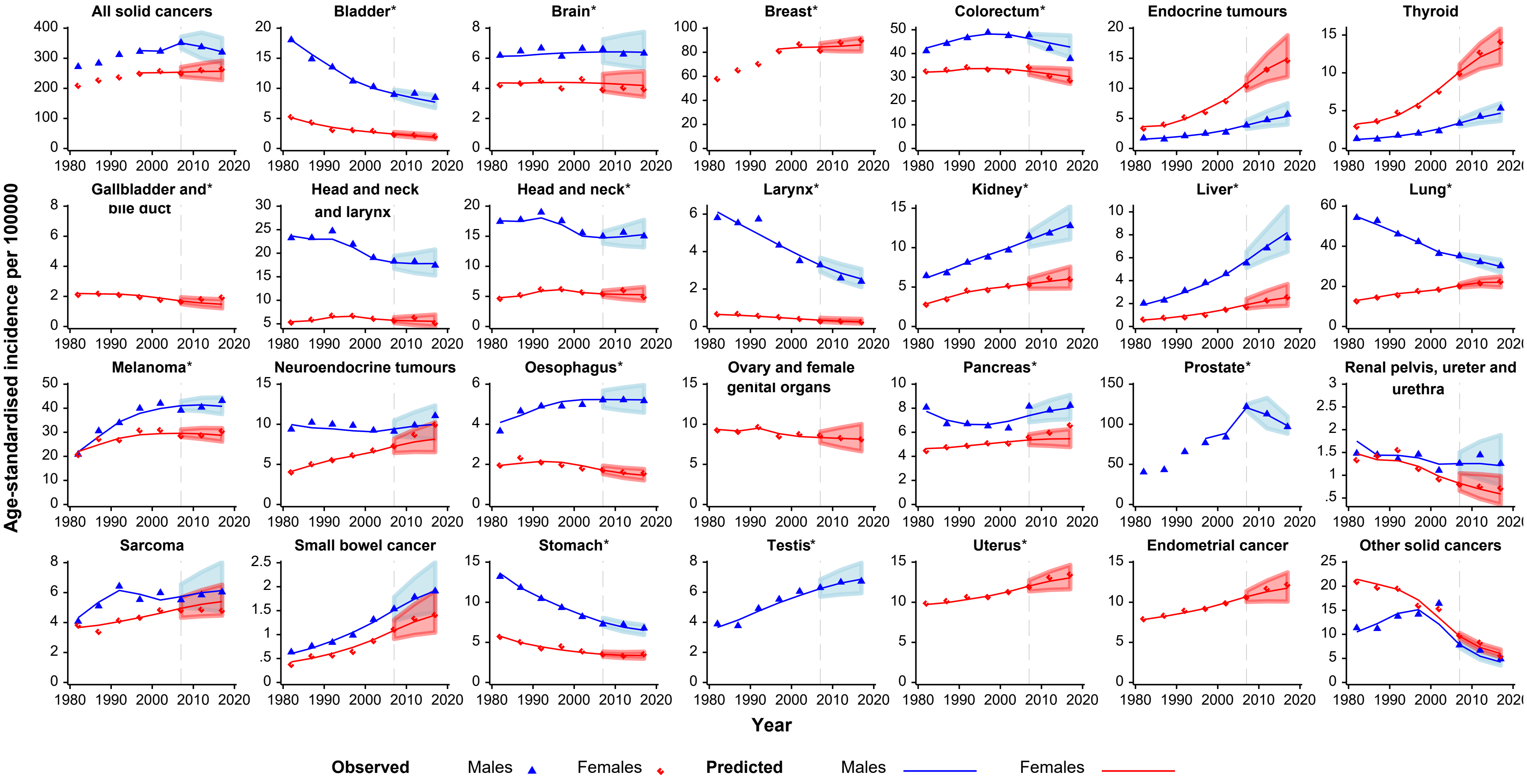

* Incidence of these cancer types/groups were reported in Luo et al 2022.[23]

For this validation, incidence was projected to 2019 based on data for 1982-2007, and then compared to observed data for 2008-2017. Rates are age-standardised to the World (Segi 1960) standard population. Shaded areas represent 95% uncertainty intervals.

Figure S2. Observed and predicted age-standardised cancer incidence rates by sex (Australia, 1982-2042)

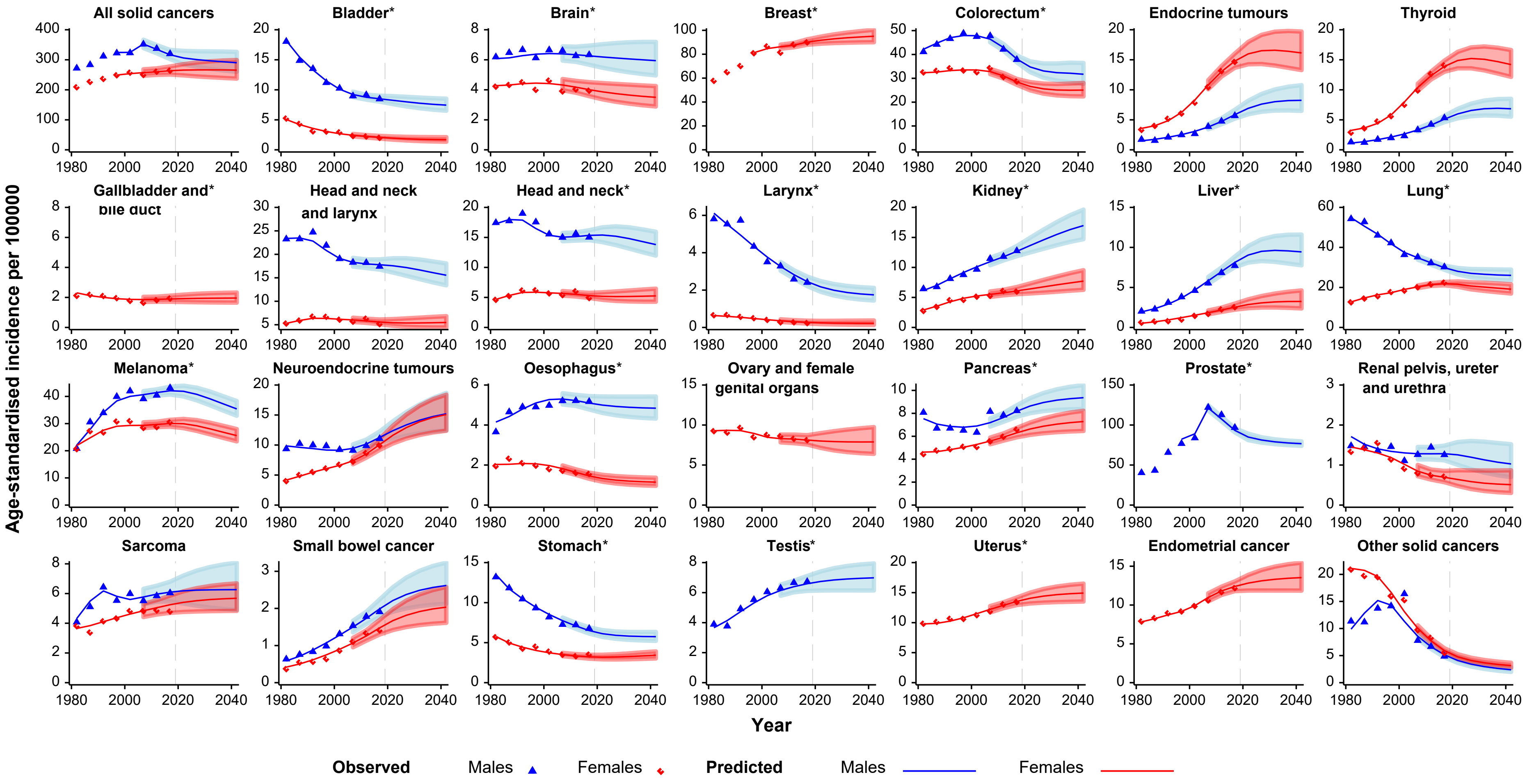

* Incidence of these cancer types/groups were reported in Luo et al 2022.[23]

Projections for all solid cancers combined were available from 1992, as data required for prostate and breast cancer projections were only available from the 1990s. Rates are age-standardised to the World (Segi 1960) standard population. Shaded areas represent 95% uncertainty intervals.

Table S4. Observed and projected age-standardised incidence rates and numbers of new cases for all solid cancers combined and selected cancer types/groups, for all stages combined (Australia, 2018-2042)

Cancer type/group	Age-standardised incidence rate per 100,000[a]			Number of cancer cases (all stages combined)			
	Observed in 2018	Projected in 2042 (95% UI)	% change in rate [b] (95% UI)	Observed in 2018	Projected in 2042 (95% UI)	% change in cases [c] (95% UI)	Total number of cases in 2019-2042 (95% UI)
All solid cancers combined (sum of 25 individual cancer types/groups)							
All solid cancers*	291·1	276·5 (247·2-311·4)	-5% (-15·1, 7·0)	126,688	184,623 (166,521-205,634)	45·7% (31·4, 62·3)	3,794,389 (3,484,982-4,145,345)
Biliary tract and gastrointestinal cancers							
Colorectum*	33·1	28·2 (24·9-32·1)	-14·8% (-24·8, -3·0)	15,448	19,349 (17,298-21,675)	25·3% (12·0, 40·3)	416,818 (382,194-455,191)
Gallbladder and bile duct*	1·5	2·1 (1·7-2·6)	40% (13·3, 73·3)	747	1,845 (1,532-2,225)	147% (105·1, 197·9)	35,754 (30,395-42,102)
Liver*	5·3	6·2 (5·0-8·0)	17% (-5·7, 50·9)	2,316	4,553 (3,724-5,615)	96·6% (60·8, 142·4)	87,540 (74,563-103,591)
Oesophagus*	3·3	2·9 (2·6-3·3)	-12·1% (-21·2, 0·0)	1,592	2,301 (2,025-2,617)	44·5% (27·2, 64·4)	46,875 (41,910-52,475)
Pancreas*	7·5	8·3 (7·4-9·3)	10·7% (-1·3, 24·0)	3,785	7,048 (6,331-7,848)	86·2% (67·3, 107·3)	133,113 (121,401-146,016)
Small bowel	1·7	2·3 (1·9-2·9)	35·3% (11·8, 70·6)	738	1,551 (1,248-1,929)	110·2% (69·1, 161·4)	28,899 (23,910-35,009)
Stomach*	5·1	4·5 (4·1-5·1)	-11·8% (-19·6, 0·0)	2,422	3,579 (3,249-3,944)	47·8% (34·1, 62·8)	70,267 (64,701-76,365)
Genitourinary tract cancers							
Bladder*	5·1	4·4 (3·8-5·1)	-13·7% (-25·5, 0·0)	2,890	4,271 (3,712-4,921)	47·8% (28·4, 70·3)	87,796 (78,400-98,464)
Kidney*	9·4	12·2 (10·4-14·4)	29·8% (10·6, 53·2)	3,802	7,270 (6,246-8,477)	91·2% (64·3, 123·0)	134,193 (118,331-152,529)
Prostate*	47	36·6 (35·3-38·0)	-22·1% (-24·9, -19·1)	21,312	26,762 (25,833-27,726)	25·6% (21·2, 30·1)	566,404 (545,704-587,976)
Renal pelvis, ureter and urethra	1·0	0·8 (0·5-1·2)	-20% (-50·0, 20·0)	562	723 (475-1,105)	28·6% (-15·5, 96·6)	16,032 (11,537-22,488)
Testis*	3·3	3·5 (3·1-4·0)	6·1% (-6·1, 21·2)	895	1,244 (1,092-1,419)	39% (22·0, 58·5)	26,067 (23,253-29,256)
Gynaecological and breast cancers							
Breast*	46·8	48·9 (46·3-51·7)	4·5% (-1·1, 10·5)	18,145	27,559 (26,224-28,967)	51·9% (44·5, 59·6)	555,472 (533,118-578,855)
Ovary/female genital organs	4·2	4·1 (3·3-5·0)	-2·4% (-21·4, 19·0)	1,769	2,592 (2,117-3,174)	46·5% (19·7, 79·4)	52,737 (44,944-61,980)
Uterus	7·0	7·7 (7·0-8·5)	10% (0·0, 21·4)	2,884	4,698 (4,252-5,190)	62·9% (47·4, 80·0)	93,328 (85,808-101,536)
Other cancer types/groups							
Brain*	5·1	4·7 (3·9-5·7)	-7·8% (-23·5, 11·8)	1,827	2,483 (2,115-2,926)	35·9% (15·8, 60·2)	52,677 (45,889-60,669)
Endocrine tumours	10·5	12·2 (9·9-15·3)	16·2% (-5·7, 45·7)	3,421	5,803 (4,775-7,098)	69·6% (39·6, 107·5)	117,802 (100,382-139,061)
Head and neck and Larynx	11·5	10·4 (8·8-12·3)	-9·6% (-23·5, 7·0)	4,690	6,485 (5,515-7,642)	38·2% (17·6, 62·9)	136,334 (119,690-155,678)
Lung*	26·0	22·4 (20·0-25·1)	-13·8% (-23·1, -3·5)	12,862	18,143 (16,309-20,186)	41·1% (26·8, 56·9)	378,219 (347,772-411,555)
Melanoma*	36·5	30·2 (27·9-32·8)	-17·3% (-23·6, -10·1)	15,130	21,038 (19,570-22,651)	39% (29·3, 49·7)	455,638 (428,996-484,545)
Neuroendocrine tumours	10·6	15·1 (12·4-18·5)	42·5% (17·0, 74·5)	4,394	9,175 (7,636-11,046)	108·8% (73·7, 151·3)	169,414 (145,675-197,490)
Sarcomas	5·6	5·9 (4·8-7·3)	5·4% (-14·3, 30·4)	2,118	3,491 (2,915-4,202)	64·7% (37·6, 98·3)	69,393 (59,486-81,287)
Other solid cancers [c]	4·3	2·8 (2·4-3·3)	-34·9% (-44·2, -23·3)	2,941	2,663 (2,328-3,051)	-9·5% (-20·8, 3·7)	63,615 (56,922-71,229)

UI: uncertainty interval.

* Estimates for these cancer types/groups as previously reported in Luo et all 2022 [23].

[a] Rates are age-standardised to the World (Segi 1960) standard population.

[b] Overall percentage change in the age-standardised incidence rate projected for 2042 compared to the age-standardised incidence rate observed in 2018.

[c] Overall percentage change in the numbers of cases projected for 2042 compared to the numbers of cases observed in 2018.

Table S5. Predicted age-standardised incidence rates and numbers of new cases for all solid cancers combined and selected cancer types/groups, for advanced disease at diagnosis (Australia, 2018-2042)

Cancer type/group	Age-standardised incidence rate per 100,000[a]			Number of cancer cases (advanced disease at diagnosis) [b]			
	Predicted in 2018	Predicted in 2042 (95% UI)	% change in rate [c] (95% UI)	Predicted in 2018	Predicted in 2042 (95% UI)	% change in cases [d] (95% UI)	Total number of cases in 2019-2042 (95% UI)
All solid cancers combined (sum of 25 individual cancer types/groups)							
All solid cancers	93·0	77·0 (65·5-90·9)	-17·2% (-29·6, -2·3)	41,567	52,277 (44,902-61,111)	25·8% (8·0, 47·0)	1,150,857 (1,014,680-1,309,427)
Biliary tract and gastrointestinal cancers							
Colorectum	13·8	9·4 (8·1-11·0)	-31·9% (-41·3, -20·3)	6,525	6,455 (5,633-7,405)	-1·1% (-13·7, 13·5)	155,506 (139,852-173,087)
Gallbladder and bile duct	1·2	1·2 (1·0-1·5)	0% (-16·7, 25·0)	615	1,074 (857-1,345)	74·6% (39·3, 118·7)	20,914 (17,034-25,659)
Liver	1·4	1·2 (0·9-1·6)	-14·3% (-35·7, 14·3)	624	855 (644-1,138)	37% (3·2, 82·4)	19,079 (15,120-24,147)
Oesophagus	1·5	1·1 (0·9-1·4)	-26·7% (-40·0, -6·7)	750	884 (720-1,082)	17·9% (-4·0, 44·3)	19,919 (16,680-23,721)
Pancreas	4·7	4·2 (3·7-4·9)	-10·6% (-21·3, 4·3)	2,393	3,609 (3,135-4,150)	50·8% (31·0, 73·4)	75,133 (66,611-84,721)
Small bowel	1·1	1·3 (1·0-1·7)	18·2% (-9·1, 54·5)	479	860 (648-1,138)	79·5% (35·3, 137·6)	16,833 (13,094-21,613)
Stomach	2·5	1·8 (1·6-2·2)	-28% (-36·0, -12·0)	1,192	1,448 (1,243-1,684)	21·5% (4·3, 41·3)	31,750 (27,889-36,100)
Genitourinary tract cancers							
Bladder	0·8	0·5 (0·4-0·6)	-37·5% (-50·0, -25·0)	458	484 (373-621)	5·7% (-18·6, 35·6)	11,621 (9,370-14,304)
Kidney	1·4	1·1 (0·8-1·5)	-21·4% (-42·9, 7·1)	580	664 (510-858)	14·5% (-12·1, 47·9)	15,344 (12,335-18,988)
Prostate	4·0	4 (3·6-4·3)	0% (-10·0, 7·5)	1,832	2,900 (2,662-3,152)	58·3% (45·3, 72·1)	56,542 (51,936-61,432)
Renal pelvis, ureter and urethra	0·3	0·3 (0·2-0·5)	0% (-33·3, 66·7)	173	258 (151-438)	49·1% (-12·7, 153·2)	5,356 (3,422-8,360)
Testis	0·5	0·3 (0·2-0·5)	-40% (-60·0, 0·0)	135	123 (86-170)	-8·9% (-36·3, 25·9)	3,166 (2,331-4,181)
Gynaecological and breast cancers							
Breast	18·2	16 (14·8-17·3)	-12·1% (-18·7, -4·9)	7,038	9,019 (8,403-9,679)	28·1% (19·4, 37·5)	197,084 (185,634-209,204)
Ovary/female genital organs	2·9	3·1 (2·5-4·0)	6·9% (-13·8, 37·9)	1,247	1,987 (1,579-2,499)	59·3% (26·6, 100·4)	38,949 (32,251-47,075)
Uterus	1·3	1·6 (1·3-1·9)	23·1% (0·0, 46·2)	540	958 (805-1,134)	77·4% (49·1, 110·0)	18,277 (15,606-21,303)
Other cancer types/groups							
Brain	0·2	0·2 (0·1-0·3)	0% (-50·0, 50·0)	62	97 (55-153)	56·5% (-11·3, 146·8)	1,931 (1,174-2,907)
Endocrine tumours	2·8	3·2 (2·4-4·2)	14·3% (-14·3, 50·0)	910	1,507 (1,171-1,946)	65·6% (28·7, 113·8)	30,682 (24,775-38,121)
Head and neck and Larynx	4·4	3·4 (2·8-4·2)	-22·7% (-36·4, -4·5)	1,805	2,150 (1,746-2,648)	19·1% (-3·3, 46·7)	48,391 (40,841-57,402)
Lung	16·6	11·5 (10·1-13·2)	-30·7% (-39·2, -20·5)	8,329	9,355 (8,247-10,610)	12·3% (-1·0, 27·4)	217,568 (197,079-240,281)
Melanoma	3·0	2·2 (2·0-2·6)	-26·7% (-33·3, -13·3)	1,270	1,565 (1,372-1,782)	23·2% (8·0, 40·3)	35,946 (32,061-40,243)
Neuroendocrine tumours	6·2	6·6 (5·3-8·4)	6·5% (-14·5, 35·5)	2,597	4,036 (3,248-5,021)	55·4% (25·1, 93·3)	83,523 (69,833-100,064)
Sarcomas	1·2	1·1 (0·8-1·5)	-8·3% (-33·3, 25·0)	446	667 (501-883)	49·6% (12·3, 98·0)	13,730 (10,674-17,584)
Other solid cancers [c]	3·0	1·4 (1·1-1·7)	-53·3% (-63·3, -43·3)	1,564	1,317 (1,116-1,555)	-15·8% (-28·6, -0·6)	33,388 (29,105-38,351)

UI: uncertainty interval.

[a] Rates are age-standardised to the World (Segi 1960) standard population.

[b] The number of new cancer cases diagnosed with advanced disease at the national level was estimated by multiplying the projected number of new cancer cases by the proportion of advanced disease at diagnosis based on the state data from the NSW Cancer Registry.

[c] Overall percentage change in the age-standardised incidence rate projected for 2042 compared to the age-standardised incidence rate predicted in 2018.

[d] Overall percentage change in the numbers of cases projected for 2042 compared to the numbers of cases predicted in 2018.

3. Multiple imputation for unknown stage at diagnosis

The use of multiple imputation (MI) for handling "unknown" stage recorded in population-based cancer registry data has been successfully validated in previous studies.[27-30] In this study, we applied a similar MI approach to impute "unknown" stage at diagnosis using the Stata 'ice' package[24] with multinomial logistic regression.[31 32]

We followed a previously described and validated approach.[32] Briefly, we first examined characteristics of cases with "unknown" stage in the NSW Cancer Registry (NSWCR) data, and selected variables for imputation: age at diagnosis, sex (if applicable), year of diagnosis, cause of death, vital status and survival time (censored at 31/12/2018), and area-level socio-economic status at diagnosis (categorised into quintiles based on the Index of Relative Socio-economic Disadvantage from highest to lowest score, with each quintile approximately account for 20% of the total population). We then generated multiple imputed datasets using the Stata 'ice' package.[24] Following recommendations, the number of imputations (***m***) for each cancer type/group was set to be at least equal to the percentage of incomplete information (here, unknown stage at diagnosis).[33] Subsequent analyses of stage distribution and stage-specific survival estimates using the imputed data were performed for each of the ***m*** imputed dataset. Estimates were then combined using Rubin's rules to obtain a summary estimate with pooled standard variances.[34-36] All multiple imputation processes were performed for each cancer type/group and sex separately.

To check the imputed data, the stage distribution after imputation was compared to SEER statistics for stage at diagnosis in 2010-2016.[37] We examined the stage distribution for localised, regional and distant stage for cases diagnosed in 2010-2016 (Figure S3), which were generally consistent with the SEER statistics for the same period.

For each cancer type/group, we also compared the estimated proportions of cases with advanced disease at diagnosis by 5-year period of diagnosis, before and after imputation (Figure S4). As the proportions of cases with advanced disease by age group were generally consistent, the overall proportions of cases with advanced disease by cancer type/group were used to estimate stage group-specific incidence in Australia.

Figure S3. Comparison of summary stage distribution for NSW after imputation with US SEER statistics, both for diagnoses 2010-2016

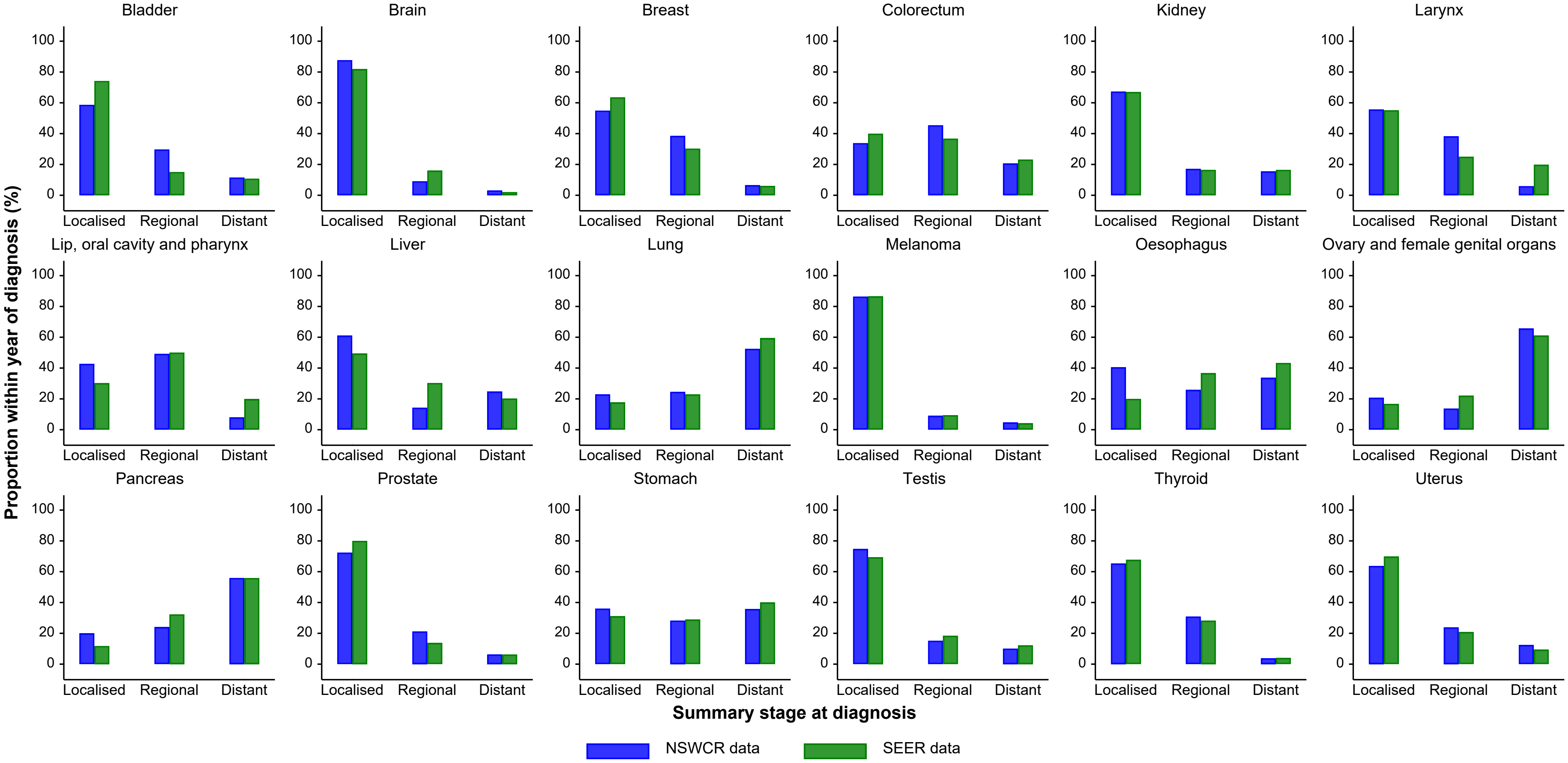

NSWCR - NSW Cancer Registry; SEER – Surveillance, Epidemiology, and End Result Program.

Stage distribution for each cancer type was extracted from the SEER Cancer Statistics Review 1975-2017.[37]

Figure S4. Proportion of cases with advanced disease at diagnosis: estimates based directly on registry data for all cases (with "unknown" stage as a separate category), complete-case analysis (excluding records with "unknown" stage), and after multiple imputation.

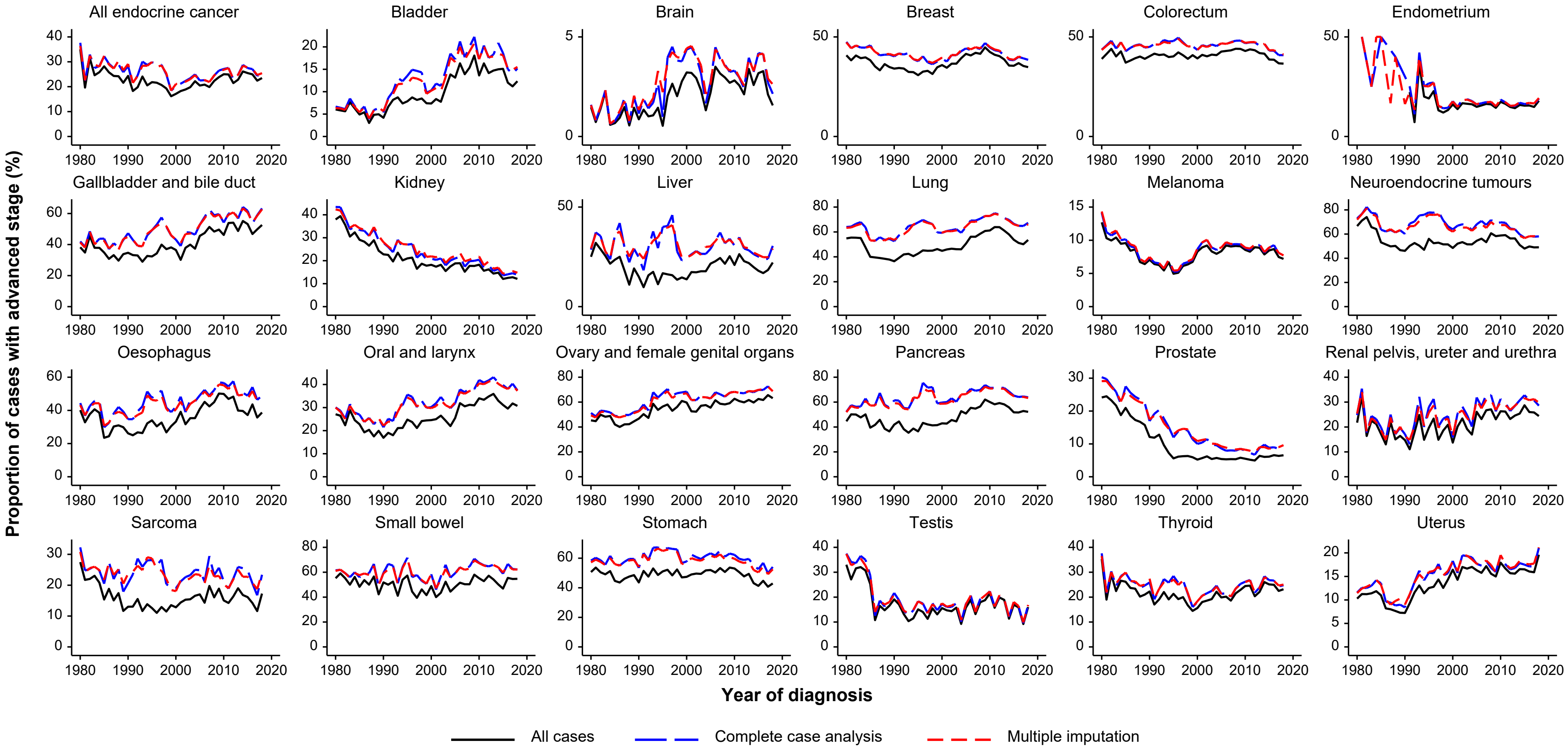

4. Survival analyses

Survival estimates were extrapolated to future periods, with linear extrapolation of log-transformed survival from the two most recent observed 5-year periods (Stata command *'ipolate'*).[24] Specifically, if s0 and s1 were the survival in the two most recent periods with mid-point years x0 and x1, respectively, survival s for a future period with midpoint year x was obtained as

$$\log(s) = \frac{\log(s_1) - \log(s_0)}{x_1 - x_0}(x - x_1) + \log(s_1).$$

Analogous to analyses of observed data, extrapolations were completed separately by cancer type, stage, and age group at diagnosis.

Figure S5. Comparison of 5-year overall survival estimates by age at diagnosis in males and females combined (Australia, 1990-2018): predicted survival (using the NSWCR data) and the observed national estimates (reported by the AIHW)

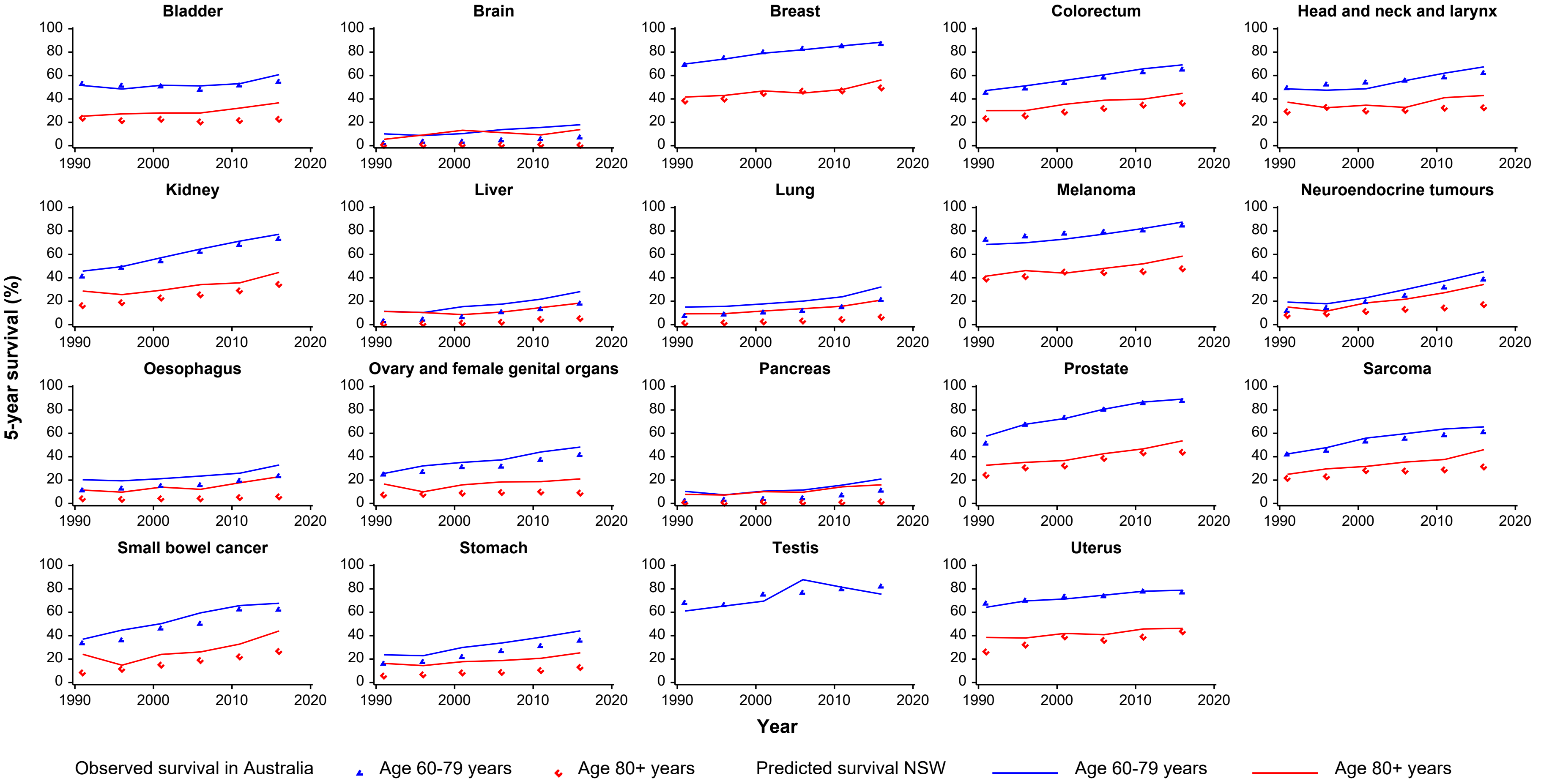

National survival estimates were not available in the Australian Institute of Health and Welfare (AIHW) statistics for some cancer types/groups (e.g. endocrine tumours, gallbladder and bile duct cancer, renal pelvis/ureter/urethra cancer, and other solid cancers). The AIHW reports survival estimates by 20-year age groups (i.e. 0-19, 20-39, […], 80+ years) and survival estimates for individuals 0-59 years of age at diagnosis as a group are not available.

Figure S6. Predicted all-cause survival estimates by age group in males and females combined (Australia, 1995-2042)

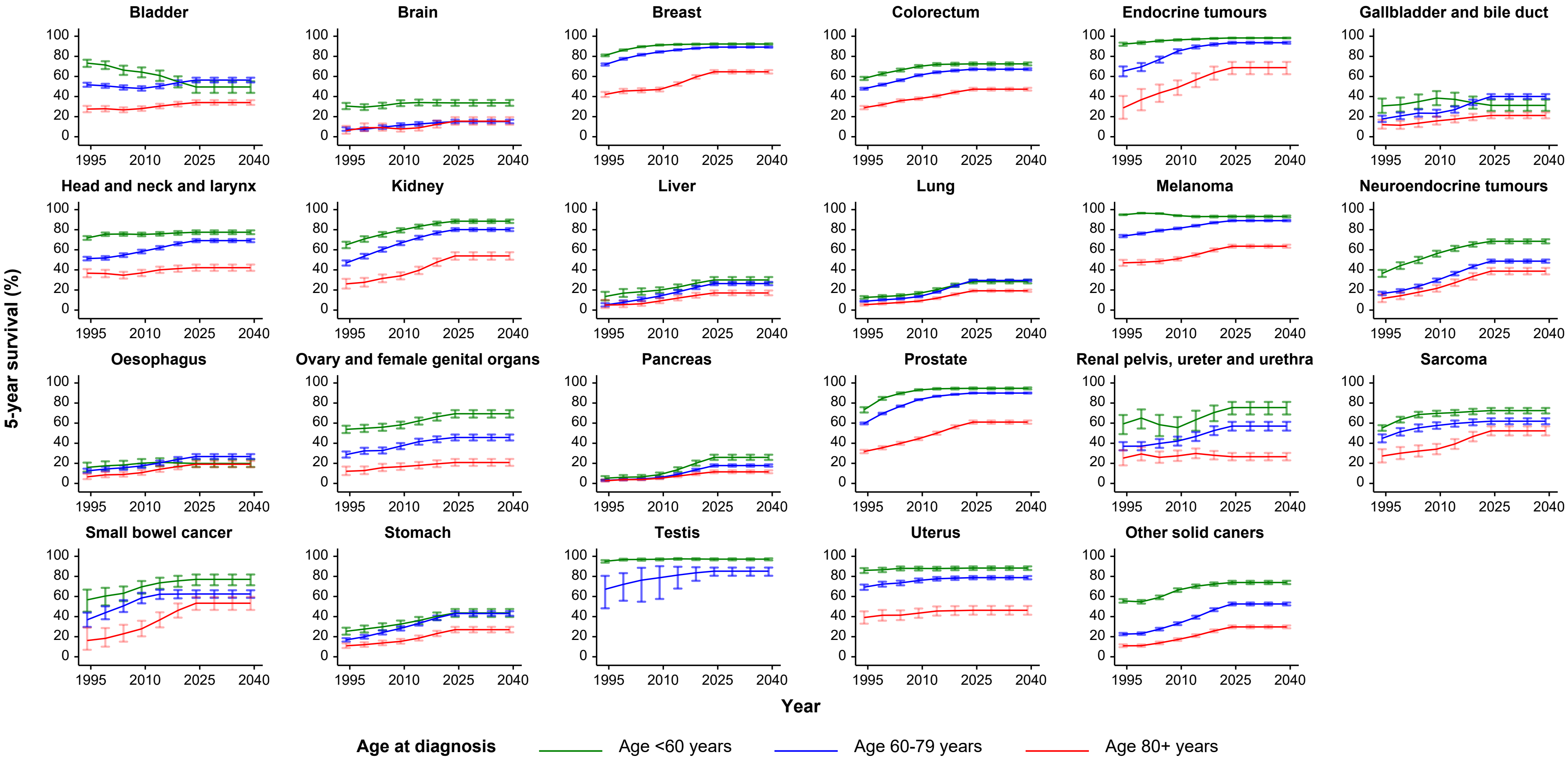

Predicted survival estimates were derived from the NSW Cancer Registry (NSWCR) data and scaled to align overall survival for NSW with the national estimates reported by the Australian Institute of Health and Welfare, see Methods.

Figure S7. Predicted all-cause survival estimates for individuals diagnosed with advanced disease by age group in males and females combined (Australia, 1995-2042)

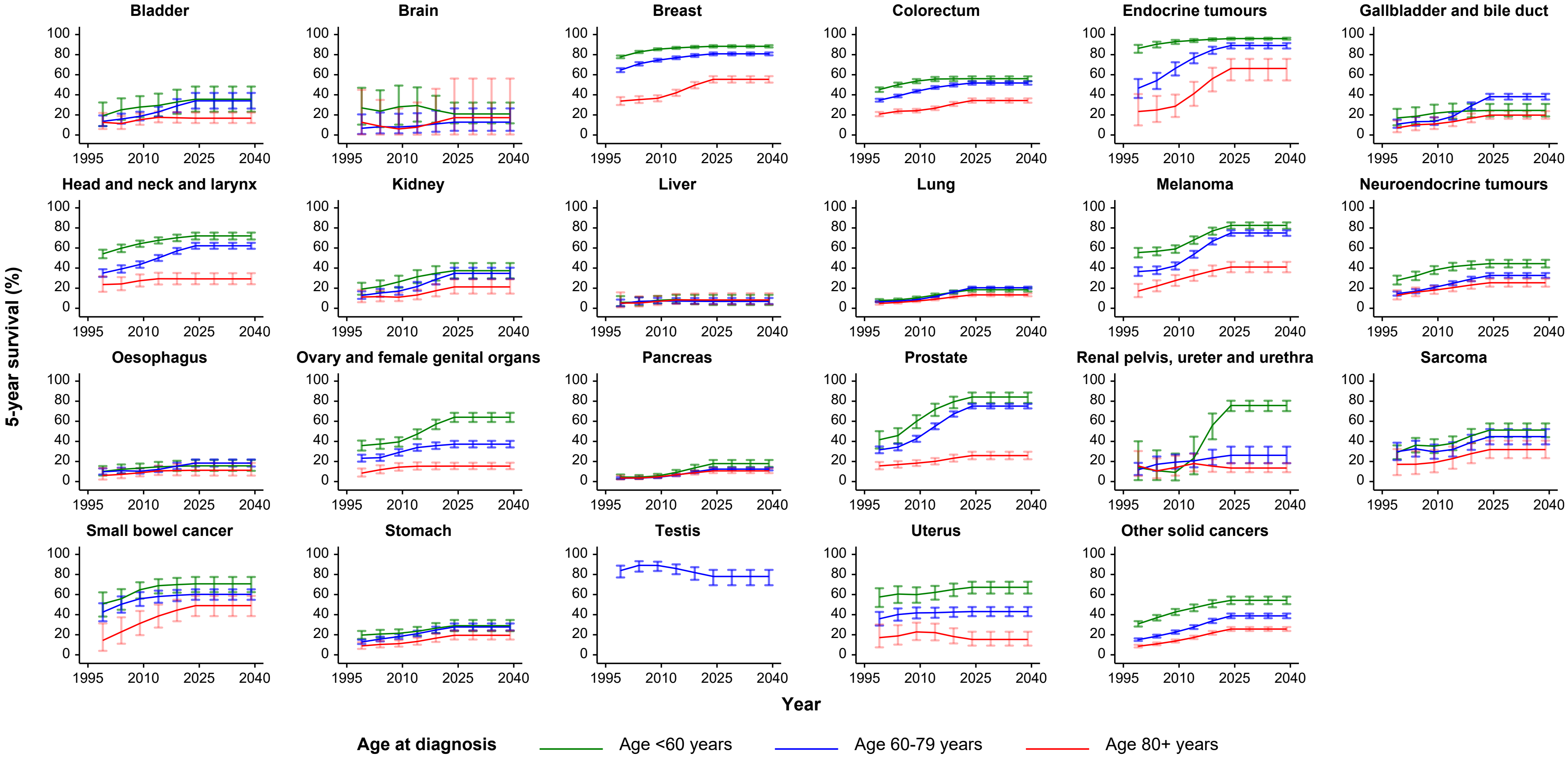

Predicted survival estimates were derived from the NSW Cancer Registry (NSWCR) data and scaled to align overall survival for NSW with the national estimates reported by the Australian Institute of Health and Welfare, see Methods.

5. Prevalence projections

In this study, we estimated cancer prevalence based on tabulated data, with prevalence estimates calculated as the running average number of individuals living with cancer over two intervals, and 1-year prevalence as a special case.

We used a modified counting method to estimate prevalence based on aggregated incidence and survival data, as described in detail elsewhere.[38] In brief, prevalence was calculated as a function of incidence and survival, using the running average number of individuals over two intervals, assuming a group of individuals within a 1-year interval contributes to cancer prevalence equally. For each cohort of new cancer cases in year j at age a, we estimated the numbers of these individuals expected to be alive at k years after the initial diagnosis in calendar year $p=j+k$ at attained age $b=a+k$ for a given cancer type/group as follows (using the notation adapted from Luo et al. 2023[38]):

$$Survivors_{pbk} = New\ cases_{ja} * \left(Observed\ survival_{(k-1)a} + Observed\ survival_{ka}\right)/2$$

This calculation was done separately for males and females, different age groups, and for k of 1 to 5 years after diagnosis.

K-year prevalence (for K of 1 to 5 years) in calendar year p was then calculated by aggregating the number of all persons previously diagnosed with cancer, alive and within k≤K years after diagnosis, with separate calculations by sex and different age groups as required.

For 1-year prevalence (e.g. k=1), we assume $Observed\ survival_{0a} = 1$ (e.g. all individuals were alive at the initial diagnosis). For example, in 2020 in Australia, 223 men age 65 were diagnosed with lung cancer and 46.9% of these men remained alive 1 year after diagnosis (with an attained age 66 years). Applying the adapted counting method described above, the number of these men counted for 1-year prevalence in 2020 was 223×(1+0.469)/2=164. (As an intuitive interpretation for this example calculation, one could consider that e.g. on the date of 31-Dec-2020, almost everyone diagnosed in December 2020 would be alive, but only about 46.9% of those diagnosed in January 2020, with survival of those diagnosed over January-December in between these extremes. Thus, the 1-year prevalence in 2020 could be roughly approximated based on the average of the 100% and 46.9% survival estimates for the end and beginning of the year.)

The 95% uncertainty intervals for the estimated number of survivors (and subsequently for the prevalence) was estimated based on the confidence intervals of the number of new cases and survival rates. The confidence intervals for the projected numbers of new cases were obtained from the APC projection models, based on the variances of the parameters in the models. The confidence intervals for survival rates were estimated using the lifetable method for survival data in the Stata *'ltable'* function, as described in the relevant documentation.[24]

This method was evaluated by comparing the average predicted 1- and 5-year prevalence estimates in 2014-2017 with the observed prevalence from the AIHW (see Figure S8).[39-42] For most cancer types/groups, the predicted prevalence estimates were found to be close to the observed prevalence and the uncertainty interval of the predicted prevalence estimate generally captured the observed prevalence (see Figure S8).

Figure S8. Comparison of the average 1- and 5-year prevalence in 2014-2017: predicted prevalence in this study and observed prevalence (reported by the AIHW)

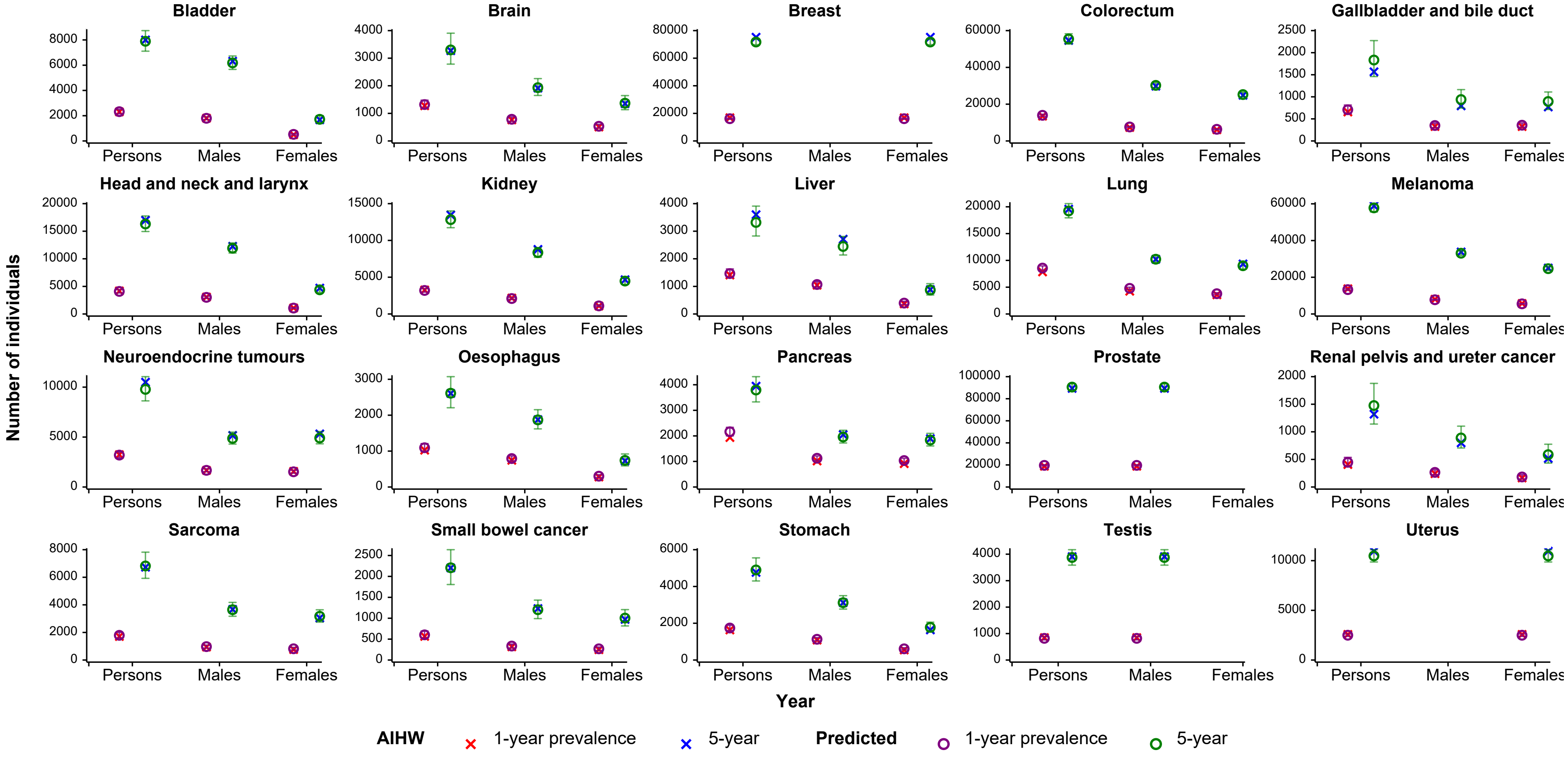

The error bars represent the 95% uncertainty intervals for prevalence estimates.

6. Estimating prevalence of advanced disease after progression post-diagnosis

For individuals with non-advanced disease at diagnosis who die from cancer, progression to advanced disease is considered to be on the clinical pathway from diagnosis to death. In this study, we only included individuals whose disease progressed to advanced disease within 5 years of initial diagnosis, as the absolute risks of disease progression and cancer death diminished sharply over time.[43] Thus, we developed a simplified method to estimate the proportion of individuals with non-advanced disease that later progressed to advanced disease post-diagnosis (see Figure S9), using the NSWCR data[44] (1989-1993 to 2014-2018).

Figure S9. Disease progression: from non-advanced stage at diagnosis to advanced disease post-diagnosis

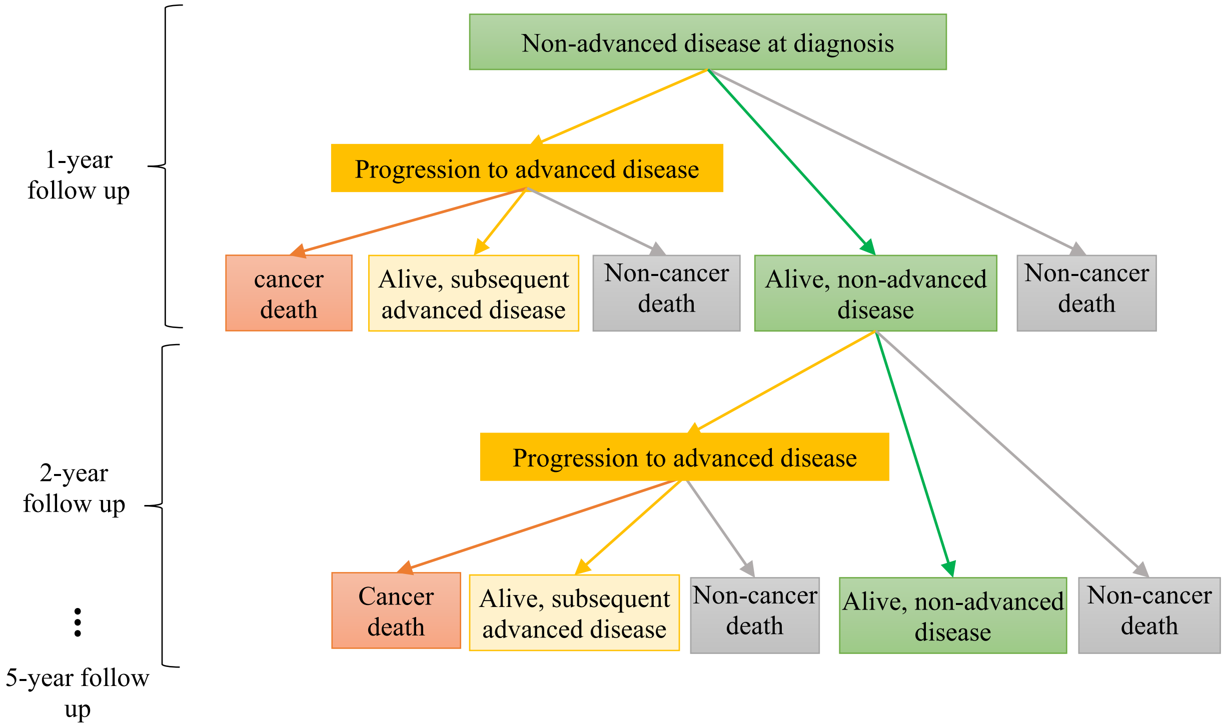

In general, for those with advanced disease at diagnosis who later died from cancer, the median survival time after the initial diagnosis was generally <6 months for most cancer types/groups (see Table S4). Hence among those diagnosed with non-advanced disease, we used time to cause-specific cancer deaths as a proxy for time to disease progression. In particular, we used the following process to estimate prevalence of individuals diagnosed with non-advanced disease who later progressed to advanced disease.

First, we considered all individuals diagnosed with non-advanced disease in a given diagnosis year (e.g. 2014). Among them, we identified those with cause-specific cancer deaths in the first year – these individuals would have head to progress to advanced disease on the pathway to cause-specific cancer deaths. However, not everyone who progressed from non-advanced to advanced disease would die from cancer in the same year – some would die from cancer in future years and others would die from other causes in the future. In the absence of other data, we used the death rate for those diagnosed with advanced disease as a proxy. Thus, we derived the following formula to estimate the proportion of disease progression (see Figure S9).

Let N denote the total number of individuals diagnosed with non-advanced disease.

Let $P_{non-advanced}$ denote the cause-specific cancer death rate for individuals diagnosed with non-advanced disease who died from the cancer.

Let D denote the number of cancer deaths for individuals diagnosed with non-advanced disease, which can be estimated as

$$D = N \times P_{non-advanced} \quad (1)$$

These individuals would have progressed to advanced disease on the pathway to cause-specific cancer deaths.

Let n denote the number of individuals diagnosed with non-advanced disease that progressed to advanced disease post-diagnosis.

Let $P_{advanced}$ denote the cause-specific cancer death rate for n individuals with advanced disease after progression post-diagnosis who died from the cancer within the first year. We used the observed cause-specific cancer death rate for those with advanced disease at diagnosis as a proxy of the cancer death rate for the individuals with advanced disease after progression post-diagnosis. Then

$$D \approx n \times P_{advanced} \quad (2)$$

Then, we back-calculated the total number of individuals with advanced disease after progression

$$n = D/P_{advanced} \quad (3)$$

Let $P_{progression}$ denote the proportion of individuals diagnosed with non-advanced disease that progressed to advanced disease post-diagnosis

$$P_{progression} = n/N \quad (4)$$

Then, substituting n from formula (3):

$$P_{progression} = \frac{D/P_{advanced}}{N} \quad (5)$$

and replacing D from formula (1):

$$P_{progression} = \frac{D/P_{advanced}}{N} = \frac{(N \times P_{non-advanced})/P_{advanced}}{N} = P_{non-adavnced}/P_{advanced} \quad (6)$$

This estimate was used for prevalence of individuals with advanced disease after progression in the first year after initial diagnosis.

For the second year after diagnosis, we followed a similar approach to identify individuals who progressed in that year (see Figure S9), adjusting for individuals who had already progressed in the first year or died from non-cancer causes. An analogous approach was used for 3-5 years post-diagnosis.

The number of individuals whose disease newly progressed to advanced disease in each year was then used analogous to incidence to calculate prevalence estimates for individuals whose disease progressed to advanced disease post-diagnosis (see main Methods text).

Table S6. Median survival (in years) for individuals diagnosed with advanced disease and who later die from cancer

	Median survival (in years) by diagnosis period						
**Cancer type/group**	**1980 - 1984**	**1985 - 1989**	**1990 - 1994**	**1995 -1999**	**2000 - 2004**	**2005 -2009**	**2010 -2014**
All endocrine tumours	0·7	0·9	1·4	1·1	1·0	0·9	0·7
Bladder	0·5	0·4	0·5	0·6	0·6	0·6	0·5
Brain	0·5	0·2	0·3	0·3	0·5	0·4	0·4
Breast	3·1	3·3	3·4	3·8	3·6	3·1	2·3
Colorectum	0·8	0·9	1·0	1·1	1·2	1·2	1·2
Gallbladder and bile duct	0·3	0·3	0·4	0·4	0·4	0·5	0·5
Head and neck and larynx	1·1	1·1	1·1	1·2	1·2	1·2	1·0
Kidney	0·4	0·5	0·4	0·5	0·4	0·5	0·5
Liver	0·1	0·2	0·2	0·3	0·3	0·3	0·3
Lung	0·4	0·4	0·4	0·4	0·4	0·4	0·5
Melanoma	1·2	1·1	0·9	0·9	1·2	1·1	0·9
Oesophagus	0·3	0·4	0·5	0·5	0·6	0·6	0·6
Ovary/female genital organs	0·7	0·8	1·1	1·3	1·6	1·3	1·3
Pancreas	0·2	0·3	0·3	0·3	0·3	0·3	0·3
Prostate	1·6	1·7	1·8	1·9	1·6	1·5	1·3
Renal pelvis, ureters and urethra	0·4	0·5	0·5	0·5	0·5	0·5	0·6
Small bowel	0·8	1·2	1·3	1·0	1·4	1·4	1·2
Stomach	0·4	0·5	0·5	0·6	0·7	0·6	0·6
Testis	1·0	0·9	1·7	0·9	1·3	2·8	1·3
Uterus	0·8	0·9	1·1	1·1	1·1	1·0	0·9

Figure S10. Estimated proportion of cancer cases diagnosed with non-advanced disease that progressed to advanced disease post-diagnosis, for all ages combined and by years since the initial diagnosis (Australia, 1992-2018)

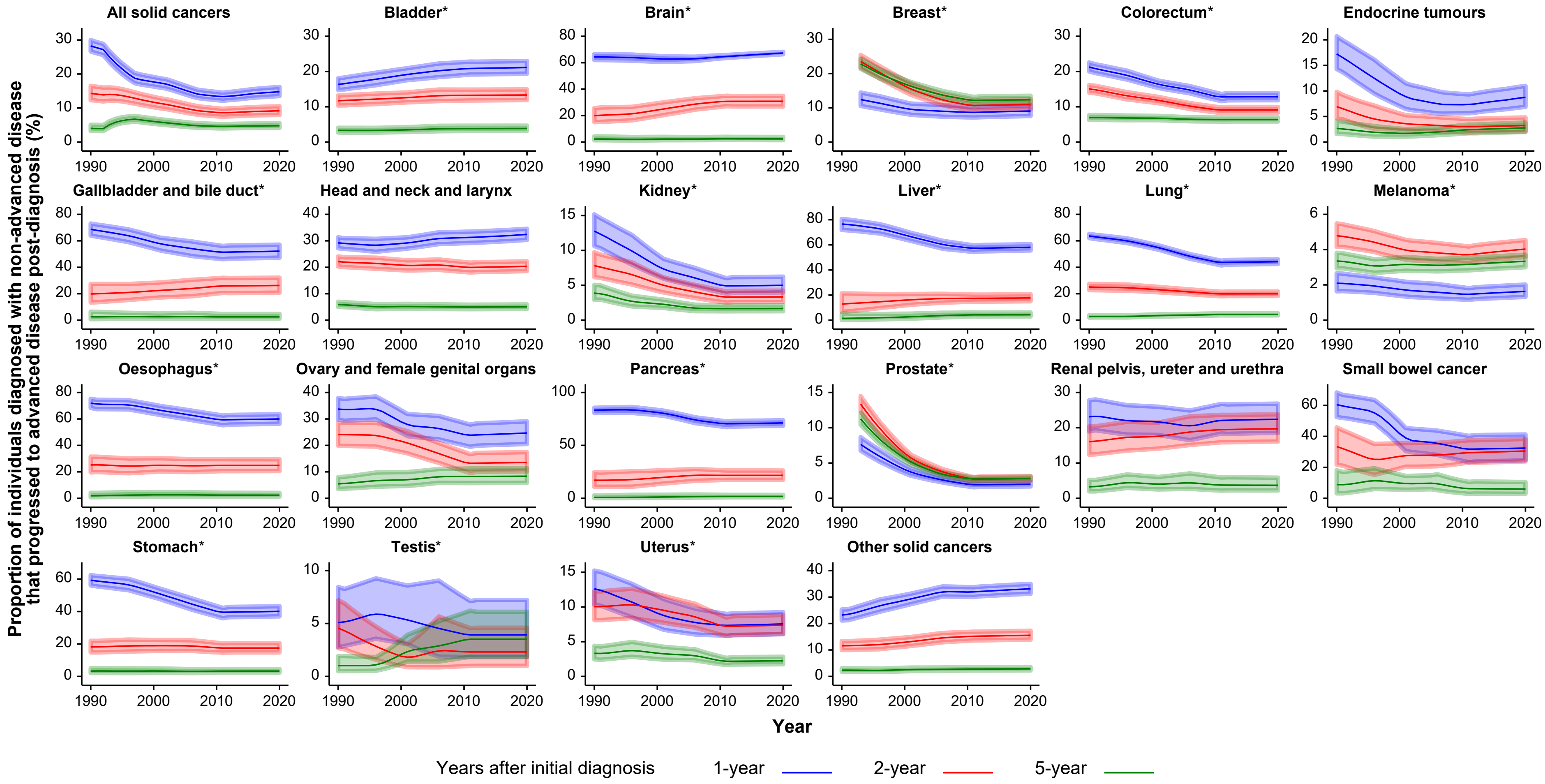

Shaded areas represent 95% uncertainty intervals.

Figure S11. Estimated proportion of individuals diagnosed with non-advanced disease that progressed to advanced disease post-diagnosis, by age at initial diagnosis and years since the initial diagnosis, based on individuals diagnosed 2009-2013

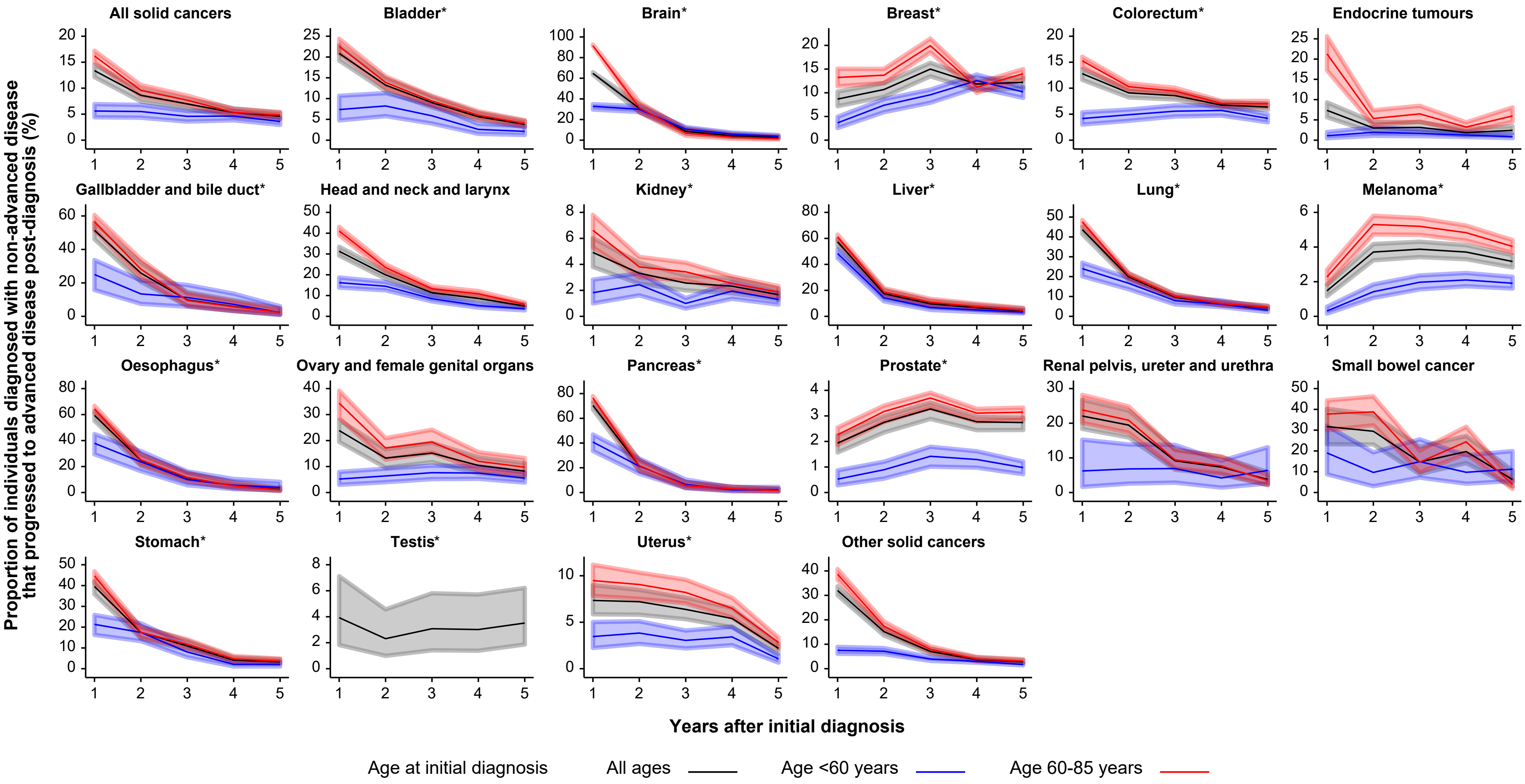

Shaded areas represent 95% uncertainty intervals. Estimated proportion of individuals diagnosed with non-advanced testicular cancer by age group is not shown due to small numbers.

7. Additional results for projected cancer prevalence

Table S7. Estimated 1-year prevalence of individuals with all solid cancers combined and selected cancer types/groups, for all stages combined (Australia, 2018-2042)

All stages combined	Total 1-year prevalence (95% uncertainty interval)		1-year prevalence (95% uncertainty interval) of individuals whose tumours exhibit a particular biomarker [a]					
	Regardless of biomarker status		Mismatch repair deficiency		Microsatellite instability		High tumour mutational burden (≥10 mut/Mb)	
	2018	2042	2018	2042	2018	2042	2018	2042
All solid cancers combined (sum of 23 individual cancer types/groups)								
All solid cancers	111,554 (105,780-117,748)	166,161 (149,130-185,939)	5,844 (2,662-9,019)	8,262 (3,680-12,846)	3,355 (2,038-4,669)	4,781 (2,793-6,767)	18,522 (13,663-23,383)	27,526 (19,697-35,357)
Biliary tract and gastrointestinal cancers								
Colorectum	14,131 (13,533-14,754)	17,773 (15,799-20,012)	1,655 (1,304-2,007)	2,082 (1,590-2,574)	1,444 (926-1,962)	1,816 (1,137-2,494)	1,204 (991-1,417)	1,514 (1,204-1,824)
Gallbladder and bile duct	783 (677-902)	1,447 (1,168-1,789)	30 (11-48)	55 (20-90)	13 (8-18)	24 (14-33)	75 (25-125)	138 (44-233)
Liver	1,689 (1,511-1,890)	3,463 (2,776-4,358)	0 (0-0)	0 (0-0)	25 (7-42)	50 (13-87)	24 (3-46)	50 (5-94)
Oesophagus	1,149 (1,035-1,277)	1,714 (1,466-2,001)	49 (21-77)	73 (30-116)	29 (13-44)	42 (19-66)	378 (300-457)	564 (433-696)
Pancreas	2,433 (2,240-2,644)	4,878 (4,292-5,535)	37 (15-59)	74 (30-118)	22 (11-34)	45 (21-68)	NA [c]	NA [c]
Small bowel	667 (571-775)	1,391 (1,092-1,768)	141 (101-181)	293 (197-389)	96 (35-157)	199 (68-330)	128 (76-181)	267 (149-385)
Stomach	1,824 (1,673-1,986)	2,912 (2,586-3,275)	160 (136-183)	254 (212-297)	156 (116-197)	249 (182-316)	254 (196-312)	405 (308-503)
Genitourinary tract cancers								
Bladder	2,457 (2,262-2,666)	3,684 (3,154-4,303)	108 (39-177)	161 (56-267)	72 (17-128)	108 (24-192)	936 (519-1,353)	1,403 (755-2,052)
Kidney	3,508 (3,244-3,804)	6,801 (5,801-7,980)	28 (6-49)	53 (11-95)	13 (6-20)	25 (11-39)	NA [d]	NA [d]
Prostate	19,679 (19,126-20,252)	25,832 (24,886-26,816)	1,217 (128-2,306)	1,598 (167-3,028)	461 (330-592)	605 (432-777)	794 (445-1,143)	1,042 (583-1,501)
Renal pelvis, ureter and urethra	464 (383-560)	588 (377-920)	21 (7-34)	26 (7-45)	14 (3-25)	18 (3-32)	177 (94-261)	224 (96-352)
Testis	859 (793-934)	1,221 (1,066-1,396)	NA [c]	NA [c]	9 (0-17)	12 (0-23)	33 (7-58)	46 (10-82)
Gynaecological and breast cancers								
Breast	17,300 (16,837-17,771)	26,777 (25,444-28,175)	221 (121-321)	342 (186-497)	100 (43-157)	154 (66-243)	1,254 (277-2,231)	1,941 (426-3,456)
Ovary and female genital organs	1,550 (1,427-1,681)	2,288 (1,843-2,835)	89 (38-140)	131 (52-211)	70 (23-117)	103 (31-175)	180 (96-265)	266 (133-400)
Uterus	2,712 (2,571-2,856)	4,451 (4,004-4,941)	728 (625-830)	1,194 (997-1,391)	594 (406-782)	975 (655-1,294)	1,167 (1,045-1,288)	1,914 (1,656-2,173)
Other cancer types/groups								
Brain	1,389 (1,227-1,567)	1,905 (1,575-2,310)	53 (0-106)	73 (0-146)	9 (4-14)	12 (5-20)	40 (1-78)	54 (0-108)
Endocrine tumours	3,294 (3,009-3,609)	5,676 (4,653-6,953)	23 (3-42)	39 (4-73)	22 (0-43)	37 (0-74)	192 (124-259)	330 (202-458)
Head and neck and Larynx	4,289 (3,969-4,633)	5,942 (5,004-7,058)	97 (6-187)	134 (7-261)	21 (11-30)	28 (15-42)	368 (78-658)	510 (101-918)
Lung	9,236 (8,816-9,678)	13,795 (12,276-15,505)	153 (43-263)	228 (62-394)	40 (20-60)	59 (29-90)	2,540 (1,470-3,611)	3,794 (2,148-5,440)
Melanoma	14,327 (13,796-14,885)	20,525 (19,042-22,146)	959 (0-1,918)	1,374 (0-2,751)	NA [c]	NA [c]	7,532 (7,029-8,034)	10,790 (9,807-11,773)
Neuroendocrine tumours	3,629 (3,293-3,997)	7,748 (6,356-9,445)	NA [c]	NA [c]	50 (13-86)	105 (26-185)	900 (792-1,008)	1,921 (1,545-2,296)
Other solid cancers [b]	2,236 (2,064-2,420)	2,126 (1,833-2,471)	65 (58-72)	62 (52-71)	60 (46-75)	58 (42-73)	313 (87-539)	298 (80-516)
Sarcomas	1,927 (1,710-2,171)	3,165 (2,590-3,872)	10 (0-19)	16 (0-32)	35 (0-69)	57 (0-114)	33 (8-59)	55 (12-97)

[a] Presence of specific biomarkers in tumours is not mutually exclusive; thus, some individuals may be included in prevalence estimates for multiple biomarkers. The underlying estimates for proportion of tumours that exhibit each pan-tumour biomarker by cancer type/group were obtained from our recent scoping review and meta-analysis, which provided estimates for all stages combined and for advanced disease where available[25] Due to limited data availability, we assumed that the proportions of tumours exhibiting each biomarker are the same for 'other solid cancers' as for all solid cancers combined. The same proportions of solid tumours exhibiting dMMR/MSI/high TMB were applied to all diagnosis periods for this study.

[b] Projections for cancers with low incidence (<2/100,000 people) were included in an aggregated group of "other solid cancers". This group includes cancers in the anus, cervix, eye and other central nervous system, other and ill-defined digestive organs, other and ill-defined sites, other male genital organs, other thoracic and respiratory organs, penis, peritoneum, placenta, vagina, vulva, unknown primary site, mesothelioma and non-melanoma skin cancer.

[c] In the published review, the reported proportion of tumours with the biomarker was 0%.[25]

[d] The published review did not estimate the proportion with high TMB for kidney cancer.[25]

Table S8. Estimated 1-year prevalence of individuals with all solid cancers combined and selected cancer types/groups, for advanced disease at diagnosis (Australia, 2018-2042)

Advanced disease at diagnosis	Total 1-year prevalence (95% uncertainty interval)		1-year prevalence (95% uncertainty interval) of individuals whose tumours exhibit a particular biomarker [a]					
	Regardless of biomarker status		Mismatch repair deficiency		Microsatellite instability		High tumour mutational burden (≥10 mut/Mb)	
	2018	2042	2018	2042	2018	2042	2018	2042
All solid cancers combined (sum of 23 individual cancer types/groups)								
All solid cancers	33,586 (30,566-36,961)	43,144 (36,480-51,194)	1,177 (678-1,672)	1,520 (827-2,217)	749 (353-1,140)	1,013 (438-1,582)	4,529 (2,864-6,197)	5,520 (3,334-7,704)
Biliary tract and gastrointestinal cancers								
Colorectum	5,741 (5,378-6,131)	5,706 (4,919-6,621)	395 (308-482)	393 (293-492)	234 (143-325)	233 (138-327)	489 (400-579)	486 (379-594)
Gallbladder and bile duct	434 (353-534)	817 (627-1,057)	17 (6-27)	31 (11-51)	7 (0-13)	12 (0-24)	NA [c]	NA [c]
Liver	373 (303-466)	528 (380-751)	NA [c]	NA [c]	2 (0-4)	3 (0-6)	14 (0-27)	19 (0-38)
Oesophagus	508 (426-608)	593 (458-765)	22 (9-35)	25 (10-41)	13 (6-20)	15 (7-23)	168 (127-208)	196 (138-253)
Pancreas	1,507 (1,346-1,687)	2,409 (2,035-2,847)	NA [c]	NA [c]	14 (7-21)	22 (11-34)	NA [c]	NA [c]
Small bowel	410 (330-509)	768 (555-1,041)	87 (59-114)	162 (102-222)	59 (21-97)	110 (35-185)	79 (45-113)	148 (77-218)
Stomach	880 (766-1,008)	1,092 (899-1,322)	50 (34-66)	62 (40-84)	73 (15-130)	90 (17-163)	389 (147-632)	483 (176-790)
Genitourinary tract cancers								
Bladder	333 (270-409)	351 (252-473)	15 (5-25)	16 (5-27)	3 (1-4)	3 (1-4)	146 (68-224)	154 (65-242)
Kidney	438 (360-529)	520 (375-698)	4 (1-7)	5 (1-8)	8 (0-16)	10 (0-19)	NA [d]	NA [d]
Prostate	1,654 (1,520-1,792)	2,599 (2,358-2,857)	58 (22-95)	91 (34-149)	110 (58-162)	172 (90-254)	118 (64-172)	185 (100-271)
Renal pelvis, ureter and urethra	119 (81-167)	169 (95-303)	6 (2-9)	8 (2-14)	1 (1-2)	2 (1-2)	52 (21-83)	74 (25-124)
Testis	129 (99-161)	118 (78-162)	NA [c]	NA [c]	2 (0-3)	2 (0-3)	5 (1-9)	5 (1-9)
Gynaecological and breast cancers								
Breast	6,730 (6,429-7,041)	8,703 (8,075-9,372)	86 (47-125)	111 (61-162)	13 (5-21)	16 (6-26)	630 (511-749)	815 (654-975)
Ovary and female genital organs	1,069 (955-1,198)	1,728 (1,351-2,201)	62 (26-97)	99 (38-160)	48 (16-81)	78 (23-133)	17 (0-34)	27 (0-55)
Uterus	456 (390-525)	824 (672-995)	123 (99-147)	221 (171-272)	81 (43-119)	145 (74-216)	84 (47-121)	151 (82-221)
Other cancer types/groups								
Brain	43 (23-74)	73 (30-129)	3 (1-4)	4 (2-7)	1 (1-1)	1 (1-1)	2 (0-3)	3 (0-5)
Endocrine tumours	851 (729-982)	1,461 (1,121-1,894)	6 (1-11)	10 (1-19)	6 (0-11)	10 (0-19)	22 (1-43)	37 (0-74)
Head and neck and Larynx	1,599 (1,415-1,803)	1,903 (1,511-2,387)	36 (2-70)	43 (2-84)	8 (5-11)	9 (5-14)	59 (7-111)	70 (7-133)
Lung	5,721 (5,372-6,094)	6,683 (5,801-7,692)	95 (26-163)	111 (30-192)	NA [c]	NA [c]	1,657 (1,138-2,176)	1,936 (1,288-2,584)
Melanoma	1,133 (1,020-1,257)	1,431 (1,231-1,663)	76 (0-153)	96 (0-193)	NA [c]	NA [c]	318 (209-428)	402 (258-546)
Neuroendocrine tumours	1,930 (1,690-2,205)	3,132 (2,459-3,991)	NA [c]	NA [c]	27 (7-46)	43 (10-75)	111 (31-192)	181 (46-315)
Other solid cancers [b]	1,165 (1,041-1,301)	985 (820-1,193)	34 (30-38)	29 (24-34)	32 (24-40)	27 (19-34)	163 (45-282)	138 (36-240)
Sarcomas	350 (271-448)	533 (373-745)	2 (0-4)	3 (0-6)	7 (0-13)	10 (0-20)	6 (2-11)	10 (2-17)

[a] Presence of specific biomarkers in tumours is not mutually exclusive; thus, some individuals may be included in prevalence estimates for multiple biomarkers. The underlying estimates for proportion of tumours that exhibit each pan-tumour biomarker by cancer type/group were obtained from our recent scoping review and meta-analysis, which provided estimates for all stages combined and for advanced disease where available [25]. Due to limited data availability, we assumed that the proportions of tumours exhibiting each biomarker are the same for 'other solid cancers' as for all solid cancers combined. The same proportions of solid tumours exhibiting dMMR/MSI/high TMB were applied to all diagnosis periods for this study.

[b] Projections for cancers with low incidence <2/100,000 people) were included in an aggregated group of "other solid cancers". This group includes cancers in the anus, cervix, eye and other central nervous system, other and ill-defined digestive organs, other and ill-defined sites, other male genital organs, penis, peritoneum, placenta, vagina, vulva, unknown primary site, mesothelioma and non-melanoma skin cancer.

[c] In the published review, the reported proportion of tumours with the biomarker was 0%.[25]

[d] The published review did not estimate the proportion with high TMB for kidney cancer.[25]

Table S9. Estimated 1-year prevalence of individuals with all solid cancers combined and selected cancer types/groups, for advanced disease after progression post-diagnosis (Australia, 2018-2042)

Advanced disease after progression	Total 1-year prevalence (95% uncertainty interval)		1-year prevalence (95% uncertainty interval) of individuals whose tumours exhibit a particular biomarker [a]					
	Regardless of biomarker status		Mismatch repair deficiency		Microsatellite instability		High tumour mutational burden (≥10 mut/Mb)	
	2018	2042	2018	2042	2018	2042	2018	2042
All solid cancers combined (sum of 23 individual cancer types/groups)								
All solid cancers	17,719 (14,762-21,171)	31,194 (24,744-39,237)	718 (359-1,078)	1,189 (559-1,824)	422 (200-642)	675 (289-1,058)	2,400 (1,406-3,395)	4,282 (2,449-6,119)
Biliary tract and gastrointestinal cancers								
Colorectum	2,284 (1,968-2,642)	3,264 (2,602-4,088)	157 (118-197)	225 (159-291)	93 (56-131)	133 (76-191)	195 (152-238)	278 (204-352)
Gallbladder and bile duct	198 (156-251)	408 (293-543)	8 (3-13)	16 (5-26)	3 (0-6)	6 (0-12)	NA [d]	NA [d]
Liver	630 (520-766)	1,517 (1,115-2,067)	NA [d]	NA [d]	4 (0-7)	8 (0-16)	23 (0-45)	54 (0-108)
Oesophagus	370 (306-435)	656 (521-811)	16 (7-26)	28 (11-45)	10 (4-15)	17 (7-26)	122 (92-153)	216 (157-276)
Pancreas	566 (507-626)	1,641 (1,396-1,913)	NA [d]	NA [d]	6 (3-8)	15 (7-23)	NA [d]	NA [d]
Small bowel	146 (109-188)	414 (275-566)	31 (20-42)	88 (51-124)	21 (7-35)	60 (17-102)	28 (15-41)	80 (39-121)
Stomach	411 (337-492)	833 (662-1,031)	24 (16-32)	47 (30-65)	34 (7-62)	69 (13-125)	182 (67-298)	369 (131-606)
Genitourinary tract cancers								
Bladder	627 (495-790)	1,014 (743-1,360)	28 (9-46)	45 (14-76)	5 (2-7)	7 (3-11)	274 (125-423)	443 (191-695)
Kidney	270 (174-403)	599 (364-977)	3 (1-4)	5 (1-9)	5 (0-10)	11 (0-22)	NA [e]	NA [e]
Prostate	1,789 (1,541-2,073)	2,296 (1,973-2,664)	63 (23-103)	81 (30-132)	119 (61-176)	152 (78-226)	128 (67-188)	164 (86-241)
Renal pelvis, ureter and urethra	117 (72-174)	139 (71-264)	6 (2-9)	7 (2-12)	1 (1-2)	1 (1-2)	52 (19-84)	61 (18-104)
Testis	86 (39-162)	133 (55-268)	NA [d]	NA [d]	1 (0-2)	2 (0-3)	4 (1-7)	5 (0-10)
Gynaecological and breast cancers								
Breast	3,769 (3,339-4,223)	6,893 (5,987-7,888)	49 (26-71)	88 (47-130)	7 (3-12)	13 (5-21)	353 (277-429)	645 (499-791)
Ovary and female genital organs	208 (166-251)	250 (180-334)	12 (5-20)	15 (6-24)	10 (3-16)	12 (3-20)	4 (0-7)	4 (0-9)
Uterus	428 (336-537)	742 (557-977)	115 (86-144)	199 (143-256)	76 (38-113)	131 (63-198)	79 (42-116)	136 (69-203)
Other cancer types/groups								
Brain	873 (611-1,184)	1,279 (834-1,819)	45 (22-68)	66 (31-102)	6 (2-10)	9 (2-15)	25 (0-51)	37 (0-75)
Endocrine tumours	392 (237-616)	900 (507-1,503)	3 (1-6)	7 (1-13)	3 (0-6)	6 (0-13)	10 (0-21)	23 (0-48)
Head and neck and Larynx	1,270 (1,079-1,479)	2,016 (1,580-2,559)	29 (2-56)	46 (2-89)	6 (4-9)	10 (5-15)	47 (6-88)	74 (7-141)
Lung	1,497 (1,363-1,640)	3,366 (2,860-3,945)	25 (7-43)	56 (15-97)	NA [d]	NA [d]	434 (295-573)	975 (641-1,309)
Melanoma	1,348 (1,068-1,695)	2,317 (1,795-2,975)	91 (0-183)	156 (0-315)	NA [d]	NA [d]	379 (231-527)	650 (390-911)
Neuroendocrine tumours [b]	-	-	-	-	-	-	-	-
Other solid cancers [c]	435 (370-504)	482 (380-597)	13 (11-15)	14 (11-18)	12 (9-15)	13 (9-17)	61 (17-106)	68 (17-119)
Sarcomas [b]	-	-	-	-	-	-	-	-

[a] Presence of specific biomarkers in tumours is not mutually exclusive; thus, some individuals may be included in prevalence estimates for multiple biomarkers. The underlying estimates for proportion of tumours that exhibit each pan-tumour biomarker by cancer type/group were obtained from our recent scoping review and meta-analysis, which provided estimates for all stages combined and for advanced disease where available.[25] Due to limited data availability, we assumed that the proportions of tumours exhibiting each biomarker are the same for 'other solid cancers' as for all solid cancers combined and the same proportion of tumours exhibiting biomarkers was used for all advanced disease regardless of treatment and disease progression before or after diagnosis. The same proportions of solid tumours exhibiting dMMR/MSI/high TMB were applied to all diagnosis periods for this study. [b] Prevalence of individuals diagnosed with non-advanced neuroendocrine tumours or sarcomas that later progressed to advanced disease post-diagnosis was not estimated, since data on causes of death for these cancer types were not available. [c] Projections for cancers with low incidence (<2/100,000 people) were included in an aggregated group of "other solid cancers". This group includes cancers in the anus, cervix, eye and other central nervous system, other and ill-defined digestive organs, other and ill-defined sites, other male genital organs, other thoracic and respiratory organs, penis, peritoneum, placenta, vagina, vulva, unknown primary site, mesothelioma and non-melanoma skin cancer.
[d] In the published review, the reported proportion of tumours with the biomarker was 0%.[25] [e] The published review did not estimate the proportion with high TMB for kidney cancer.[25]

Table S10. Estimated 2-year prevalence of individuals with all solid cancers combined and selected cancer types/groups, for all stages combined (Australia, 2018-2042)

All stages combined	Total 1-year prevalence (95% uncertainty interval)		1-year prevalence (95% uncertainty interval) of individuals whose tumours exhibit a particular biomarker [a]					
	Regardless of biomarker status		Mismatch repair deficiency		Microsatellite instability		High tumour mutational burden (≥10 mut/Mb)	
	2018	2042	2018	2042	2018	2042	2018	2042
All solid cancers combined (sum of 23 individual cancer types/groups)								
All solid cancers	205,083 (193,859-217,094)	308,146 (275,626-345,916)	10,938 (4,910-16,966)	15,540 (6,821-24,275)	6,205 (3,779-8,626)	8,886 (5,195-12,583)	33,864 (25,144-42,583)	51,073 (36,700-65,442)
Biliary tract and gastrointestinal cancers								
Colorectum	26,368 (25,166-27,610)	33,256 (29,444-37,587)	3,088 (2,430-3,747)	3,895 (2,968-4,822)	2,693 (1,726-3,661)	3,397 (2,124-4,670)	2,245 (1,846-2,645)	2,832 (2,246-3,417)
Gallbladder and bile duct	1,244 (1,045-1,476)	2,389 (1,887-3,013)	47 (18-77)	90 (32-149)	21 (13-28)	39 (23-55)	119 (39-199)	228 (71-385)
Liver	2,590 (2,280-2,947)	5,580 (4,396-7,137)	NA [c]	NA [c]	38 (11-64)	80 (21-140)	37 (5-70)	79 (8-151)
Oesophagus	1,769 (1,559-2,003)	2,691 (2,242-3,219)	75 (31-119)	114 (46-182)	44 (20-67)	66 (30-103)	582 (456-709)	886 (666-1,105)
Pancreas	3,357 (3,047-3,701)	7,178 (6,196-8,296)	51 (21-81)	108 (43-174)	31 (15-47)	65 (31-100)	NA [c]	NA [c]
Small bowel	1,213 (1,022-1,425)	2,580 (1,985-3,322)	256 (181-330)	543 (359-727)	174 (63-285)	369 (124-615)	233 (136-330)	495 (271-718)
Stomach	3,003 (2,713-3,312)	4,953 (4,317-5,658)	262 (221-304)	432 (355-510)	257 (189-324)	423 (306-541)	418 (320-515)	689 (518-860)
Genitourinary tract cancers								
Bladder	4,335 (3,956-4,742)	6,562 (5,552-7,745)	190 (68-311)	287 (99-475)	127 (29-225)	192 (41-343)	1,651 (913-2,389)	2,499 (1,335-3,663)
Kidney	6,600 (6,066-7,192)	13,026 (11,047-15,348)	51 (11-92)	101 (20-182)	24 (11-37)	47 (21-74)	NA [d]	NA [d]
Prostate	38,614 (37,478-39,784)	50,229 (48,283-52,241)	2,388 (250-4,525)	3,106 (324-5,888)	904 (647-1,161)	1,176 (840-1,511)	1,558 (873-2,243)	2,026 (1,133-2,919)
Renal pelvis, ureter and urethra	807 (646-998)	1,020 (636-1,626)	36 (12-59)	45 (12-78)	24 (5-43)	30 (4-56)	308 (159-456)	389 (162-616)
Testis	1,695 (1,562-1,840)	2,413 (2,100-2,762)	NA [c]	NA [c]	16 (0-32)	23 (0-46)	64 (14-114)	91 (19-162)
Gynaecological and breast cancers								
Breast	33,656 (32,729-34,601)	52,451 (49,792-55,240)	429 (235-624)	669 (365-973)	194 (83-305)	302 (128-476)	2,440 (538-4,341)	3,802 (833-6,770)
Ovary and female genital organs	2,784 (2,542-3,047)	4,140 (3,305-5,172)	160 (67-252)	237 (93-382)	125 (40-210)	186 (55-316)	324 (172-475)	481 (238-724)
Uterus	5,159 (4,869-5,454)	8,512 (7,622-9,490)	1,384 (1,187-1,581)	2,283 (1,900-2,666)	1,130 (772-1,488)	1,864 (1,250-2,477)	2,219 (1,983-2,454)	3,660 (3,155-4,166)
Other cancer types/groups								
Brain	2,165 (1,878-2,484)	3,024 (2,434-3,757)	83 (0-166)	115 (0-233)	14 (5-22)	19 (7-31)	61 (1-122)	86 (0-172)
Endocrine tumours	6,384 (5,820-7,003)	11,186 (9,149-13,722)	44 (6-82)	76 (8-144)	41 (0-82)	72 (0-145)	371 (239-503)	650 (396-903)
Head and neck and Larynx	7,999 (7,363-8,682)	11,121 (9,301-13,293)	180 (11-349)	250 (11-488)	38 (21-55)	53 (28-78)	686 (145-1,227)	953 (187-1,720)
Lung	14,083 (13,347-14,859)	22,216 (19,589-25,190)	233 (65-401)	367 (98-636)	61 (30-91)	95 (46-144)	3,873 (2,238-5,509)	6,110 (3,446-8,774)
Melanoma	27,703 (26,632-28,813)	40,177 (37,192-43,426)	1,854 (0-3,709)	2,688 (0-5,385)	NA [c]	NA [c]	14,563 (13,578-15,548)	21,120 (19,161-23,079)
Neuroendocrine tumours	6,144 (5,517-6,837)	13,673 (11,096-16,834)	NA [c]	NA [c]	84 (22-145)	185 (45-326)	1,523 (1,329-1,717)	3,389 (2,699-4,078)
Other solid cancers [b]	3,771 (3,445-4,123)	3,625 (3,097-4,252)	109 (96-122)	105 (88-122)	102 (77-127)	98 (71-124)	528 (145-910)	507 (134-880)
Sarcomas	3,550 (3,120-4,029)	5,897 (4,763-7,291)	18 (0-35)	29 (0-59)	63 (0-127)	105 (0-212)	61 (15-107)	101 (22-180)

[a] Presence of specific biomarkers in tumours is not mutually exclusive; thus, some individuals may be included in prevalence estimates for multiple biomarkers. The underlying estimates for proportion of tumours that exhibit each pan-tumour biomarker by cancer type/group were obtained from our recent scoping review and meta-analysis, which provided estimates for all stages combined and for advanced disease where available.[25] Due to limited data availability, we assumed that the proportions of tumours exhibiting each biomarker are the same for 'other solid cancers' as for all solid cancers combined. The same proportions of solid tumours exhibiting dMMR/MSI/high TMB were applied to all diagnosis periods for this study.

[b] Projections for cancers with low incidence (<2/100,000 people) were included in an aggregated group of "other solid cancers". This group includes cancers in the anus, cervix, eye and other central nervous system, other and ill-defined digestive organs, other and ill-defined sites, other male genital organs, other thoracic and respiratory organs, penis, peritoneum, placenta, vagina, vulva, unknown primary site, mesothelioma and non-melanoma skin cancer.

[c] In the published review, the reported proportion of tumours with the biomarker was 0%.[25]

[d] The published review did not estimate the proportion with high TMB for kidney cancer.[25]

Table S11. Estimated 2-year prevalence of individuals with all solid cancers combined and selected cancer types/groups, for advanced disease at diagnosis (Australia, 2018-2042)

Advanced disease at diagnosis	Total 1-year prevalence (95% uncertainty interval)		1-year prevalence (95% uncertainty interval) of individuals whose tumours exhibit a particular biomarker [a]					
	Regardless of biomarker status		Mismatch repair deficiency		Microsatellite instability		High tumour mutational burden (≥10 mut/Mb)	
	2018	2042	2018	2042	2018	2042	2018	2042
All solid cancers combined (sum of 23 individual cancer types/groups)								
All solid cancers	57,121 (51,355-63,540)	74,834 (62,530-89,859)	2,043 (1,175-2,915)	2,686 (1,442-3,935)	1,290 (607-1,967)	1,773 (763-2,784)	7,372 (4,654-10,086)	9,251 (5,565-12,940)
Biliary tract and gastrointestinal cancers								
Colorectum	10,294 (9,566-11,063)	10,302 (8,799-12,050)	708 (550-866)	708 (526-891)	419 (256-582)	419 (248-591)	877 (714-1,040)	878 (680-1,076)
Gallbladder and bile duct	657 (511-846)	1,314 (977-1,749)	25 (9-41)	50 (17-83)	10 (0-19)	19 (0-38)	NA [c]	NA [c]
Liver	490 (376-646)	696 (466-1,059)	NA [c]	NA [c]	3 (0-5)	4 (0-8)	18 (0-35)	25 (0-51)
Oesophagus	727 (581-906)	850 (620-1,163)	31 (12-50)	36 (13-59)	18 (8-28)	21 (9-34)	240 (175-305)	280 (189-372)
Pancreas	2,006 (1,752-2,294)	3,410 (2,795-4,152)	NA [c]	NA [c]	19 (9-28)	31 (14-48)	NA [c]	NA [c]
Small bowel	741 (581-935)	1,412 (992-1,958)	156 (105-208)	297 (182-413)	106 (37-176)	202 (62-342)	142 (79-205)	271 (138-404)
Stomach	1,358 (1,145-1,599)	1,746 (1,387-2,179)	77 (51-103)	99 (63-135)	112 (22-202)	144 (26-261)	601 (223-979)	772 (274-1,270)
Genitourinary tract cancers								
Bladder	518 (395-664)	549 (370-780)	23 (8-39)	24 (7-42)	4 (2-6)	4 (2-6)	227 (101-352)	240 (96-384)
Kidney	703 (545-882)	863 (594-1,207)	6 (1-10)	7 (1-13)	13 (0-25)	16 (0-31)	NA [d]	NA [d]
Prostate	3,055 (2,781-3,341)	4,803 (4,317-5,320)	107 (40-175)	169 (63-275)	202 (106-299)	317 (165-470)	218 (117-318)	342 (183-501)
Renal pelvis, ureter and urethra	179 (111-272)	250 (131-475)	8 (2-14)	11 (3-20)	2 (1-2)	2 (1-3)	79 (29-128)	110 (33-186)
Testis	251 (189-316)	221 (143-311)	NA [c]	NA [c]	3 (0-5)	3 (0-5)	10 (2-18)	9 (2-16)
Gynaecological and breast cancers								
Breast	13,000 (12,386-13,632)	16,931 (15,668-18,277)	166 (91-242)	216 (117-315)	24 (9-39)	31 (12-51)	1,217 (986-1,447)	1,584 (1,270-1,898)
Ovary and female genital organs	1,880 (1,658-2,127)	3,070 (2,371-3,956)	108 (45-171)	176 (67-285)	85 (27-142)	138 (39-236)	30 (0-60)	48 (0-98)
Uterus	798 (671-938)	1,449 (1,161-1,785)	214 (170-259)	389 (296-482)	141 (73-208)	255 (129-381)	146 (81-212)	266 (142-390)
Other cancer types/groups								
Brain	63 (25-119)	112 (32-224)	4 (1-6)	6 (1-11)	1 (1-1)	1 (0-2)	2 (0-4)	4 (0-8)
Endocrine tumours	1,629 (1,388-1,894)	2,853 (2,177-3,721)	11 (2-21)	20 (2-37)	11 (0-21)	19 (0-38)	41 (1-82)	72 (0-145)
Head and neck and Larynx	2,908 (2,537-3,313)	3,477 (2,717-4,421)	66 (4-128)	78 (3-154)	14 (8-21)	17 (9-25)	107 (13-201)	128 (12-243)
Lung	8,246 (7,654-8,881)	10,118 (8,650-11,816)	137 (38-235)	167 (44-291)	NA [c]	NA [c]	2,388 (1,635-3,142)	2,931 (1,933-3,928)
Melanoma	2,075 (1,832-2,330)	2,691 (2,278-3,159)	139 (0-279)	181 (0-363)	NA [c]	NA [c]	583 (378-787)	755 (480-1,031)
Neuroendocrine tumours	3,074 (2,631-3,575)	5,144 (3,944-6,691)	NA [c]	NA [c]	42 (11-73)	70 (16-124)	177 (48-306)	296 (73-519)
Other solid cancers [b]	1,847 (1,616-2,105)	1,600 (1,302-1,977)	54 (46-62)	47 (37-56)	50 (37-63)	43 (31-56)	259 (70-447)	224 (57-391)
Sarcomas	583 (425-779)	910 (602-1,328)	3 (0-6)	5 (0-10)	11 (0-22)	17 (0-34)	10 (2-18)	16 (3-29)

[a] Presence of specific biomarkers in tumours is not mutually exclusive; thus, some individuals may be included in prevalence estimates for multiple biomarkers. The underlying estimates for proportion of tumours that exhibit each pan-tumour biomarker by cancer type/group were obtained from our recent scoping review and meta-analysis, which provided estimates for all stages combined and for advanced disease where available [25]. Due to limited data availability, we assumed that the proportions of tumours exhibiting each biomarker are the same for 'other solid cancers' as for all solid cancers combined. The same proportions of solid tumours exhibiting dMMR/MSI/high TMB were applied to all diagnosis periods for this study.

[b] Projections for cancers with low incidence <2/100,000 people) were included in an aggregated group of "other solid cancers". This group includes cancers in the anus, cervix, eye and other central nervous system, other and ill-defined digestive organs, other and ill-defined sites, other male genital organs, other thoracic and respiratory organs, penis, peritoneum, placenta, vagina, vulva, unknown primary site, mesothelioma and non-melanoma skin cancer.

[c] In the published review, the reported proportion of tumours with the biomarker was 0%.[25]

[d] The published review did not estimate the proportion with high TMB for kidney cancer.[25]

Table S12. Estimated 2-year prevalence of individuals with all solid cancers combined and selected cancer types/groups, for advanced disease after progression post-diagnosis (Australia, 2018-2042)

Advanced disease after progression	Total 1-year prevalence (95% uncertainty interval) Regardless of biomarker status		1-year prevalence (95% uncertainty interval) of individuals whose tumours exhibit a particular biomarker [a]					
			Mismatch repair deficiency		Microsatellite instability		High tumour mutational burden (≥10 mut/Mb)	
	2018	2042	2018	2042	2018	2042	2018	2042
All solid cancers combined (sum of 23 individual cancer types/groups)								
All solid cancers	29,946 (24,381-36,524)	53,327 (41,385-68,538)	1,220 (591-1,853)	2,055 (932-3,190)	721 (335-1,101)	1,161 (493-1,828)	3,931 (2,289-5,578)	7,158 (4,068-10,258)
Biliary tract and gastrointestinal cancers								
Colorectum	4,005 (3,416-4,674)	5,803 (4,581-7,339)	276 (205-347)	399 (280-519)	163 (96-230)	237 (133-340)	341 (264-419)	495 (360-629)
Gallbladder and bile duct	297 (221-393)	653 (453-896)	12 (4-19)	25 (8-42)	5 (0-9)	10 (0-19)	NA [d]	NA [d]
Liver	818 (636-1,052)	1,985 (1,362-2,896)	NA [d]	NA [d]	5 (0-9)	10 (0-21)	29 (0-58)	70 (0-143)
Oesophagus	520 (412-640)	931 (695-1,221)	22 (9-36)	40 (15-65)	13 (6-21)	23 (10-37)	172 (124-219)	307 (211-403)
Pancreas	738 (644-834)	2,283 (1,881-2,746)	NA [d]	NA [d]	7 (4-11)	21 (10-32)	NA [d]	NA [d]
Small bowel	260 (187-340)	755 (485-1,053)	55 (35-76)	159 (90-229)	38 (12-63)	108 (30-187)	50 (26-74)	145 (68-222)
Stomach	624 (492-766)	1,322 (1,011-1,687)	36 (23-49)	75 (46-104)	52 (10-94)	109 (19-199)	276 (98-455)	585 (201-968)
Genitourinary tract cancers								
Bladder	956 (714-1,263)	1,558 (1,076-2,206)	42 (13-71)	69 (20-117)	7 (3-10)	11 (5-17)	418 (183-652)	681 (278-1,083)
Kidney	418 (255-652)	969 (559-1,647)	4 (1-7)	8 (1-15)	8 (0-16)	17 (0-36)	NA [e]	NA [e]
Prostate	3,263 (2,773-3,812)	4,188 (3,564-4,905)	115 (41-188)	147 (53-241)	216 (109-322)	277 (140-413)	232 (121-343)	298 (156-441)
Renal pelvis, ureter and urethra	176 (98-284)	204 (96-410)	8 (2-14)	9 (2-17)	2 (1-2)	2 (1-3)	77 (25-129)	90 (24-155)
Testis	164 (72-316)	247 (97-507)	NA [d]	NA [d]	2 (0-4)	3 (0-6)	7 (1-13)	10 (0-19)
Gynaecological and breast cancers								
Breast	7,166 (6,319-8,049)	13,274 (11,488-15,238)	92 (49-135)	170 (90-250)	14 (5-22)	25 (9-40)	671 (524-817)	1,242 (959-1,525)
Ovary and female genital organs	358 (281-440)	432 (306-588)	21 (8-34)	25 (9-41)	17 (5-28)	20 (5-34)	6 (0-12)	7 (0-14)
Uterus	735 (560-945)	1,275 (932-1,719)	198 (144-251)	342 (240-445)	130 (63-196)	224 (106-343)	135 (70-200)	234 (116-351)
Other cancer types/groups								
Brain	1,264 (734-1,930)	1,946 (1,004-3,176)	66 (27-104)	101 (36-165)	8 (2-15)	13 (2-23)	36 (0-75)	55 (0-117)
Endocrine tumours	732 (427-1,167)	1,733 (962-2,927)	5 (1-10)	12 (0-24)	5 (0-10)	12 (0-24)	19 (0-39)	44 (0-92)
Head and neck and Larynx	2,265 (1,890-2,676)	3,627 (2,787-4,677)	51 (3-100)	82 (3-161)	11 (6-16)	18 (9-26)	83 (9-157)	133 (12-254)
Lung	2,106 (1,896-2,335)	5,023 (4,201-5,980)	35 (10-61)	83 (22-145)	NA [d]	NA [d]	610 (413-808)	1,455 (948-1,963)
Melanoma	2,409 (1,872-3,083)	4,273 (3,253-5,568)	162 (0-328)	286 (0-582)	NA [d]	NA [d]	676 (406-946)	1,199 (709-1,689)
Neuroendocrine tumours [b]	-	-	-	-	-	-	-	-
Other solid cancers [c]	664 (553-788)	769 (588-968)	20 (16-23)	23 (17-28)	18 (13-23)	21 (14-28)	93 (25-162)	108 (26-190)
Sarcomas [b]	-	-	-	-	-	-	-	-

[a] Presence of specific biomarkers in tumours is not mutually exclusive; thus, some individuals may be included in prevalence estimates for multiple biomarkers. The underlying estimates for proportion of tumours that exhibit each pan-tumour biomarker by cancer type/group were obtained from our recent scoping review and meta-analysis, which provided estimates for all stages combined and for advanced disease where available [25]. Due to limited data availability, we assumed that the proportions of tumours exhibiting each biomarker are the same for 'other solid cancers' as for all solid cancers combined and the same proportion of tumours exhibiting biomarkers was used for all advanced disease regardless of treatment and disease progression before or after diagnosis. The same proportions of solid tumours exhibiting dMMR/MSI/high TMB were applied to all diagnosis periods for this study. [b] Prevalence of individuals diagnosed with non-advanced neuroendocrine tumours or sarcomas that later progressed to advanced disease post-diagnosis was not estimated, since data on causes of death for these cancer types were not available. [c] Projections for cancers with low incidence (<2/100,000 people) were included in an aggregated group of "other solid cancers". This group includes cancers in the anus, cervix, eye and other central nervous system, other and ill-defined digestive organs, other and ill-defined sites, other male genital organs, other thoracic and respiratory organs, penis, peritoneum, placenta, vagina, vulva, unknown primary site, mesothelioma and non-melanoma skin cancer.

[d] In the published review, the reported proportion of tumours with the biomarker was 0%.[25] [e] The published review did not estimate the proportion with high TMB for kidney cancer.[25]

Figure S12. Projected 5-year prevalence of individuals with tumours exhibiting key biomarkers, for relevant cancer types/groups and all stages combined (A), advanced disease at diagnosis (B) and advanced disease after progression post-diagnosis (C) (Australia, 2018-2042)

(A) All stages combined

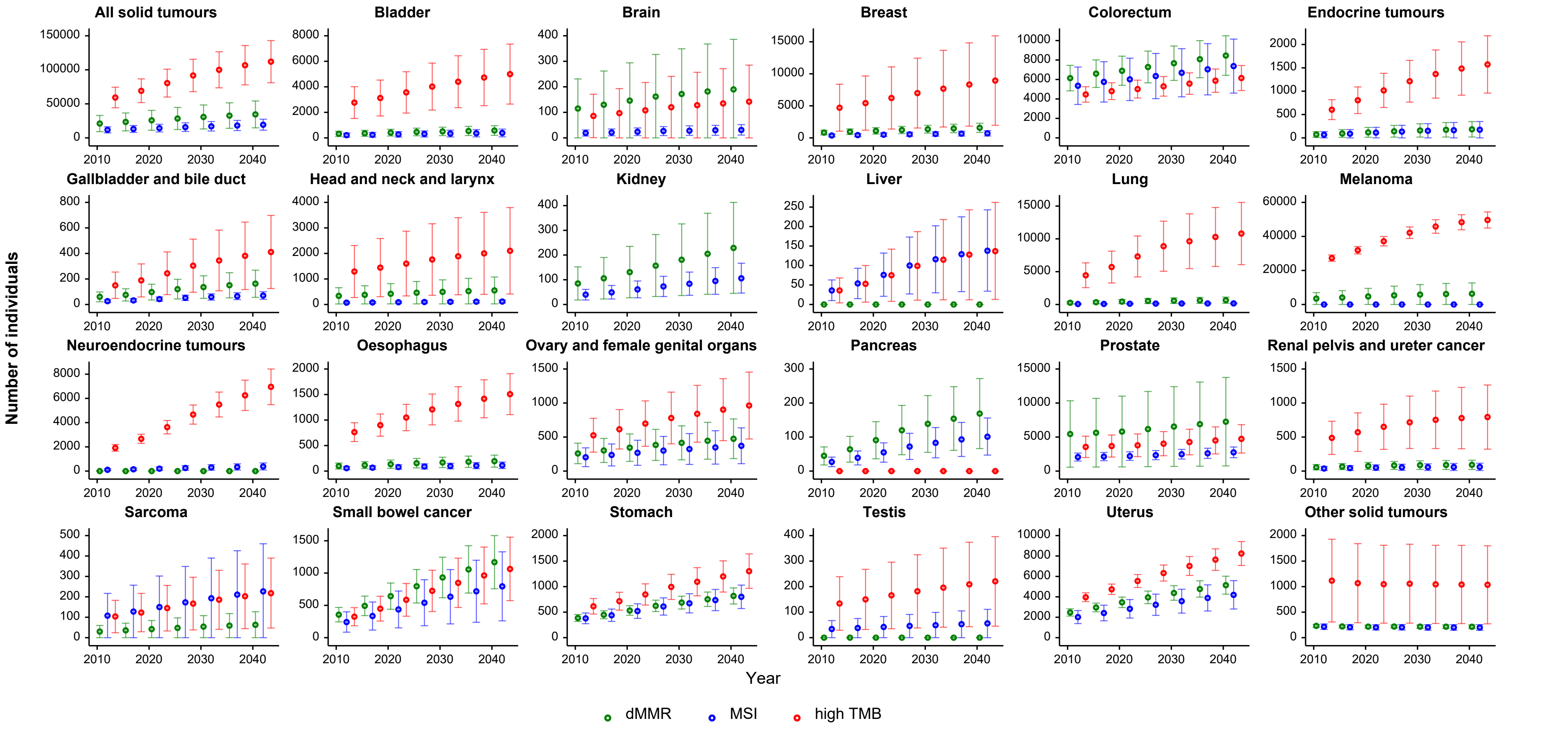

dMMR – mismatch repair deficiency; MSI – microsatellite instability; high TMB – high tumour mutational burden (≥10 mutations/Mb). Error bars represent 95% uncertainty intervals. Presence of specific biomarkers in tumours is not mutually exclusive; thus, some individuals may be included in prevalence estimates for multiple biomarkers.

(B) Advanced disease at diagnosis

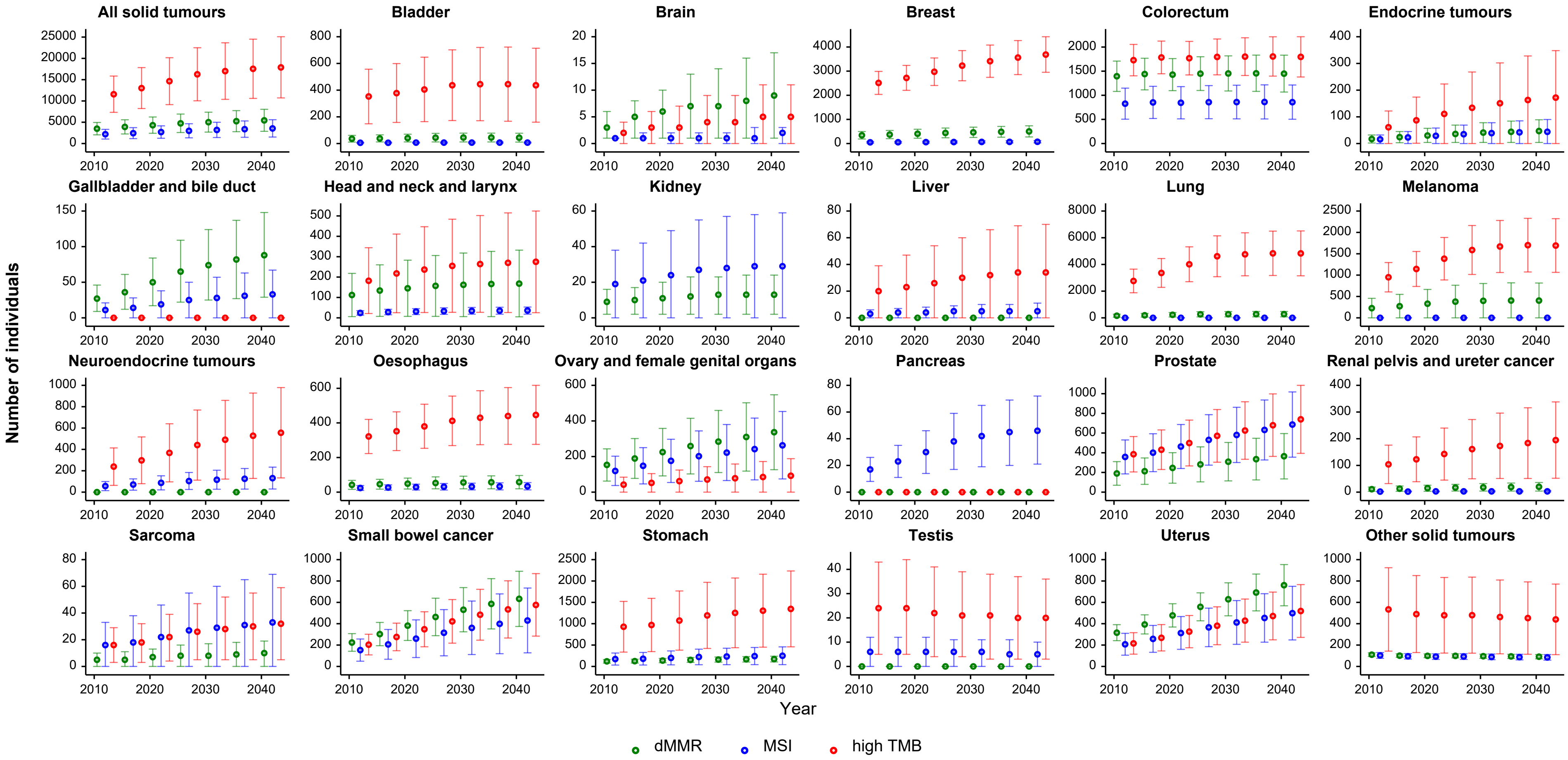

dMMR – mismatch repair deficiency; MSI – microsatellite instability; high TMB – high tumour mutational burden (≥10 mutations/Mb). Error bars represent 95% uncertainty intervals. Presence of specific biomarkers in tumours is not mutually exclusive; thus, some individuals may be included in prevalence estimates for multiple biomarkers.

(C) Advanced disease after progression post-diagnosis

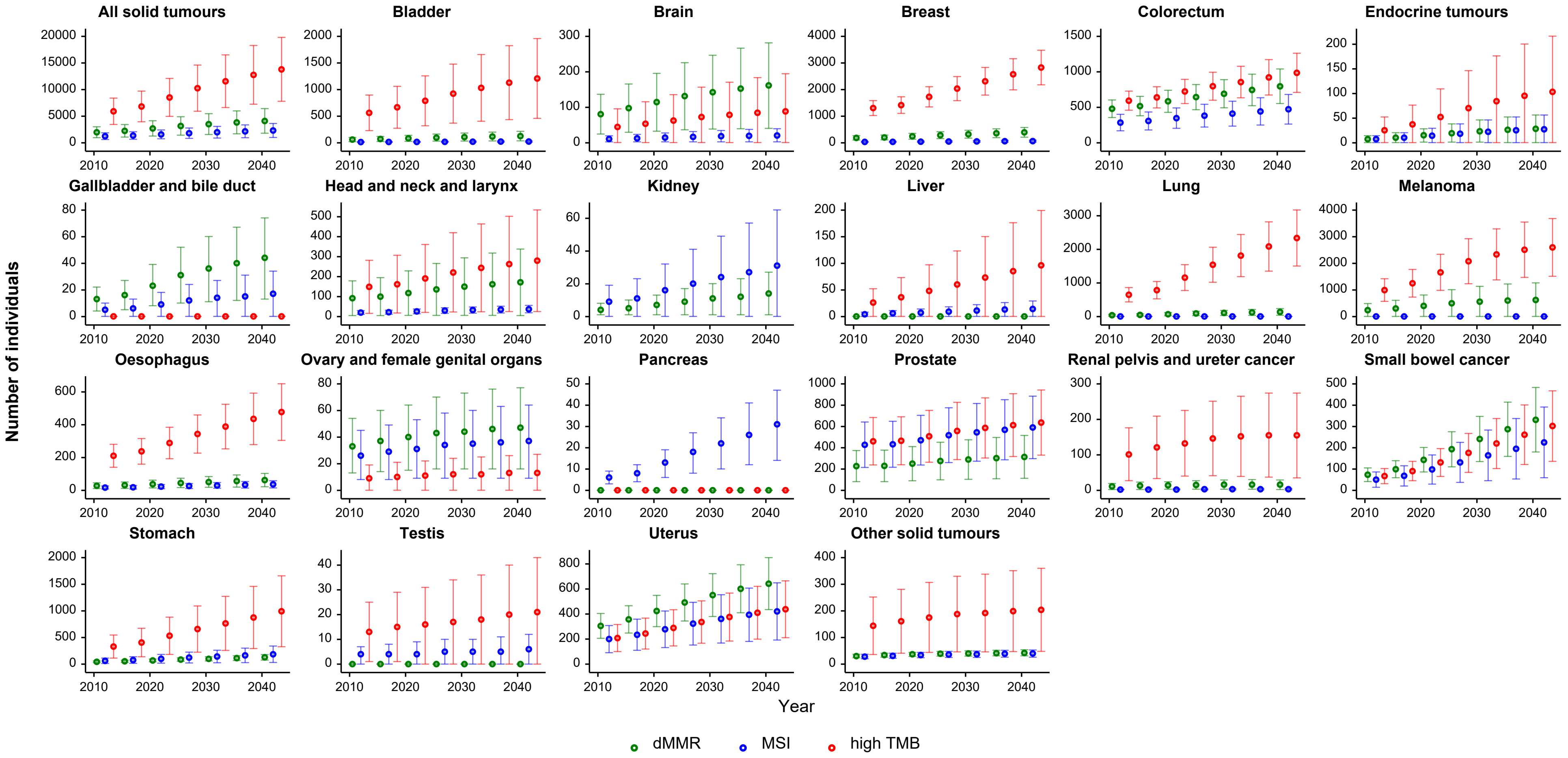

dMMR – mismatch repair deficiency; MSI – microsatellite instability; high TMB – high tumour mutational burden (≥10 mutations/Mb). Error bars represent 95% uncertainty intervals. Presence of specific biomarkers in tumours is not mutually exclusive; thus, some individuals may be included in prevalence estimates for multiple biomarkers.

Note: Prevalence of individuals diagnosed with non-advanced neuroendocrine tumours or sarcomas that later progressed to advanced disease post-diagnosis were not estimated, since cause-specific death data for these cancer types were not available.

8. Sensitivity analyses

Table S13. Sensitivity analyses using alternative assumptions for future survival, showing resulting projected 5-year prevalence of individuals with all solid cancers combined, regardless of biomarker status (Australia, 2042)

Stage of disease	Analysis		Projected 5-year prevalence of individuals with all solid cancers in 2042 (95% uncertainty interval)		
	Main analysis	Sensitivity analysis	Main analysis	Sensitivity analysis	% change [a]
All stages combined	Survival extrapolated to 2023, then constant to 2042	Survival constant from 2018	675,722 (602,939, 760,446)	655,455 (584,241, 738,644)	-3·0% (-3·1%, -2·9%)
	Survival extrapolated to 2023, then constant to 2042	Survival constant from 2028	675,722 (602,939, 760,446)	694,078 (619,918, 780,116)	2·7% (2·8%, 2·6%)
	Scaled NSW survival estimates	Unscaled NSW survival estimates	675,722 (602,939, 760,446)	708,471 (631,865, 797,018)	4·8% (4·8%, 4·8%)
Advanced disease at diagnosis	Survival extrapolated to 2023, then constant to 2042	Survival constant from 2018	151,199 (124,877, 183,900)	143,830 (118,316, 175,692)	-4·9% (-5·3%, -4·5%)
	Survival extrapolated to 2023, then constant to 2042	Survival constant from 2028	151,199 (124,877, 183,900)	158,158 (131,108, 19,1459)	4·6% (5%, 4·1%)
	Scaled NSW survival estimates	Unscaled NSW survival estimates	147,336 (121,713, 179,146)	155,731 (128,503, 187,995)	5·7% (5·6%, 4·9%)
Advanced disease after progression post-diagnosis	Survival extrapolated to 2023, then constant to 2042	Survival constant from 2018	105,582 (80,346, 13,9042)	100,461 (76,034, 133,504)	-4·9% (-5·4%, -4%)
	Survival extrapolated to 2023, then constant to 2042	Survival constant from 2028	105,582 (80,346, 139,042)	110,261 (84,423, 143,953)	4·4% (5·1%, 3·5%)

[a] % change in the projected prevalence obtained from the sensitivity analysis compared with the main analysis.

9. References

1. Australian government Department of Health and Aged Care. The Pharmaceutical Benefits Scheme. Ratified Outcome Statement – September 2024 PBAC meeting [Available from: https://www.pbs.gov.au/info/industry/listing/elements/pbac-meetings/pbac-outcomes/recommendations-made-by-the-pbac-september-2024-intracycle-meeting accessed 10/12/2024.

2. FDA approves new dosing regimen for pembrolizumab [updated 04/29/2020. Available from: https://www.fda.gov/drugs/resources-information-approved-drugs/fda-approves-new-dosing-regimen-pembrolizumab accessed 12/02/2024.

3. Australian Government. Department of Health and Aged Care. Therapeutic Goods Administration. Prescription medicines registrations: KEYTRUDA (Merck Sharp & Dohme (Australia) Pty Ltd) - Tumour Mutational Burden-High (TMB-H) cancer [updated 28/09/2021. Available from: https://www.tga.gov.au/resources/prescription-medicines-registrations/keytruda-merck-sharp-dohme-australia-pty-ltd-13 accessed 10/01/2024.

4. Australian Government. Department of Health and Aged Care. Expenditure and Prescriptions Report 1 July 2022 to 30 June 2023 [Available from: https://www.pbs.gov.au/statistics/expenditure-prescriptions/2022-2023/PBS-Expenditure-prescriptions-report-2022-23.pdf accessed 24/08/2023.

5. Australian Government. Department of Health and Aged Care. Therapeutic Goods Administration. Australian Public Assessment Report for Keytruda. August 2023 [Available from: https://www.tga.gov.au/resources/auspar/auspar-keytruda-3 accessed 02/02/2024.

6. Australian Government. Department of Health and Aged Care. Therapeutic Goods Administration. Australian Public Assessment Report for Opdivo. June 2023 [Available from: https://www.tga.gov.au/resources/auspar/auspar-opdivo accessed 02/02/2024.

7. Australian Government. Department of Health and Aged Care. Therapeutic Goods Administration. Australian Public Assessment Report for Nivolumab and ipilimumab. April 2021 [Available from: https://www.tga.gov.au/resources/auspar/auspar-nivolumab-and-ipilimumab accessed 02/02/2024.

8. Australian Government. Department of Health and Aged Care. Therapeutic Goods Administration. Australian Public Assessment Report for Atezolizumab. June 2020 [Available from: https://www.tga.gov.au/resources/auspar/auspar-atezolizumab-0 accessed 02/02/2024.

9. Australian Government. Department of Health and Aged Care. Therapeutic Goods Administration. Australian Public Assessment Report for Cemiplimab. November 2020 [Available from: https://www.tga.gov.au/resources/auspar/auspar-cemiplimab accessed 02/02/2024.

10. Australian Government. Department of Health and Aged Care. Therapeutic Goods Administration. Australian Public Assessment Report for Avelumab. May 2019 [Available from: https://www.tga.gov.au/resources/auspar/auspar-avelumab accessed 02/02/2024.

11. Australian Government. Department of Health and Aged Care. Therapeutic Goods Administration. Australian Public Assessment Report for Imfinzi. December 2023 [Available from: https://www.tga.gov.au/resources/auspar/auspar-imfinzi accessed 02/02/2024.

12. Australian Government. Department of Health and Aged Care. Therapeutic Goods Administration. Australian Public Assessment Report for Imjudo. December 2023 [Available from: https://www.tga.gov.au/resources/auspar/auspar-imjudo accessed 02/02/2024.

13. NCCN Clinical Practice Guidelines in Oncology (NCCN Guidelines®): Colon Cancer. Version 1.2024 — January 29, 2024 [Available from: https://www.nccn.org/professionals/physician_gls/pdf/colon.pdf accessed 08/02/2024.

14. NCCN Clinical Practice Guidelines in Oncology (NCCN Guidelines®): Rectal Cancer. Version 1.2024 — January 29, 2024 [Available from: https://www.nccn.org/professionals/physician_gls/pdf/rectal.pdf accessed 08/02/2024.

15. NCCN Clinical Practice Guidelines in Oncology (NCCN Guidelines®): Uterine Neoplasms. Version 1.2024 — September 20, 2023 [Available from: https://www.nccn.org/professionals/physician_gls/pdf/uterine.pdf accessed 08/02/2024.

16. NCCN Clinical Practice Guidelines in Oncology (NCCN Guidelines®): Gastric Cancer. Version 3.2023 — January 26, 2024 [Available from: https://www.nccn.org/professionals/physician_gls/pdf/gastric.pdf accessed 08/02/2024.

17. NCCN Clinical Practice Guidelines in Oncology (NCCN Guidelines®): Mesothelioma: Pleural. Version 1.2024 — November 21, 2023 [Available from: https://www.nccn.org/professionals/physician_gls/pdf/meso_pleural.pdf accessed 08/02/2024.
18. NCCN Clinical Practice Guidelines in Oncology (NCCN Guidelines®): Merkel Cell Carcinoma. Version 1.2024 — November 22, 2023 [Available from: https://www.nccn.org/professionals/physician_gls/pdf/mcc.pdf accessed 08/02/2024.
19. NCCN Clinical Practice Guidelines in Oncology (NCCN Guidelines®): Pancreatic Adenocarcinoma. Version 1.2024 — December 13, 2023 [Available from: https://www.nccn.org/professionals/physician_gls/pdf/pancreatic.pdf accessed 08/02/2024.
20. NCCN Clinical Practice Guidelines in Oncology (NCCN Guidelines®): Kidney Cancer. Version 2.2024 — January 3, 2024 [Available from: https://www.nccn.org/professionals/physician_gls/pdf/kidney.pdf accessed 08/02/2024.
21. NCCN Clinical Practice Guidelines in Oncology (NCCN Guidelines®): Small Bowel Adenocarcinoma. Version 1.2024 — December 20, 2023 [Available from: https://www.nccn.org/professionals/physician_gls/pdf/small_bowel.pdf accessed 08/02/2024.
22. NCCN Clinical Practice Guidelines in Oncology (NCCN Guidelines®): Neuroendocrine and Adrenal Tumors. Version 1.2023 — August 2, 2023 [Available from: https://www.nccn.org/professionals/physician_gls/pdf/neuroendocrine.pdf accessed 08/02/2024.
23. Luo Q, O'Connell DL, Yu XQ, et al. Cancer incidence and mortality in Australia from 2020 to 2044 and an exploratory analysis of the potential effect of treatment delays during the COVID-19 pandemic: a statistical modelling study. *The Lancet Public health* 2022;7(6):e537-e48. doi: 10.1016/s2468-2667(22)00090-1 [published Online First: 2022/06/07]
24. StataCorp. 2023. Stata 18 Base Reference Manual. College Station, TX. Stata Press.
25. Kang YJ, O'Haire S, Franchini F, et al. A scoping review and meta-analysis on the prevalence of pan-tumour biomarkers (dMMR, MSI, high TMB) in different solid tumours. *Sci Rep* 2022;12(1):20495. doi: 10.1038/s41598-022-23319-1 [published Online First: 2022/11/29]
26. Australian Institute of Health and Welfare. Cancer data in Australia: Updating sarcoma reporting [Available from: https://www.aihw.gov.au/reports/cancer/cancer-data-in-australia/contents/cancer-data-commentaries/updating-sarcoma-reporting accessed 31/08/2023.
27. Eisemann N, Waldmann A, Katalinic A. Imputation of missing values of tumour stage in population-based cancer registration. *BMC medical research methodology* 2011;11:129. doi: 10.1186/1471-2288-11-129 [published Online First: 2011/09/21]
28. Falcaro M, Nur U, Rachet B, Carpenter JR. Estimating excess hazard ratios and net survival when covariate data are missing: strategies for multiple imputation. *Epidemiology (Cambridge, Mass)* 2015;26(3):421-8. doi: 10.1097/ede.0000000000000283 [published Online First: 2015/03/17]
29. Luo Q, Lew JB, Steinberg J, et al. Trends in colon and rectal cancer mortality in Australia from 1972 to 2015 and associated projections to 2040. *Scientific reports* 2022;12(1):3994. doi: 10.1038/s41598-022-07797-x [published Online First: 2022/03/09]
30. Luo Q, O'Connell DL, Kahn C, Yu XQ. Colorectal cancer metastatic disease progression in Australia: A population-based analysis. *Cancer Epidemiol* 2017;49:92-100. doi: 10.1016/j.canep.2017.05.012 [published Online First: 2017/06/10]
31. Luo Q, Egger S, Yu XQ, et al. Validity of using multiple imputation for "unknown" stage at diagnosis in population-based cancer registry data. *PLOS ONE* 2017;12(6):e0180033. doi: 10.1371/journal.pone.0180033
32. White IR, Royston P, Wood AM. Multiple imputation using chained equations: Issues and guidance for practice. *Stat Med* 2011;30(4):377-99. doi: 10.1002/sim.4067 [published Online First: 2011/01/13]
33. Graham JW, Olchowski AE, Gilreath TD. How many imputations are really needed? Some practical clarifications of multiple imputation theory. *Prevention science : the official journal of the Society for Prevention Research* 2007;8(3):206-13. doi: 10.1007/s11121-007-0070-9 [published Online First: 2007/06/06]
34. Marshall A, Altman DG, Holder RL, Royston P. Combining estimates of interest in prognostic modelling studies after multiple imputation: current practice and guidelines. *BMC Med Res Methodol* 2009;9:57. doi: 1471-2288-9-57 [pii]

10.1186/1471-2288-9-57 [published Online First: 2009/07/30]

35. Carpenter JR, Kenward M. Multiple Imputation and Its Application. Chichester: John Wiley & Sons 2013.
36. Morisot A, Bessaoud F, Landais P, et al. Prostate cancer: net survival and cause-specific survival rates after multiple imputation. *BMC Med Res Methodol* 2015;15:54. doi: 10.1186/s12874-015-0048-4
37. Howlader. N, Noone. AM, Krapcho. M, et al. SEER Cancer Statistics Review, 1975-2017. In: National Cancer Institute, ed. Bethesda, MD, 2020.
38. Luo Q, Jenkin D, Weber MF, et al. Multiple myeloma incidence, mortality, and prevalence estimates and projections, Australia, 1982-2043: a statistical modelling study. *Med J Aust* 2024;221(2):103-10. doi: 10.5694/mja2.52366 [published Online First: 2024/07/14]
39. Australian Institute of Health and Welfare (AIHW) 2019 Cancer Data in Australia; Canberra: AIHW. Archived.
40. Australian Institute of Health and Welfare (AIHW) 2020 Cancer Data in Australia; Canberra: AIHW. Archived.
41. Australian Institute of Health and Welfare (AIHW) 2021 Cancer Data in Australia; Canberra: AIHW. Archived.
42. Australian Institute of Health and Welfare (AIHW) 2022 Cancer Data in Australia; Canberra: AIHW. Archived.
43. Baade PD, Youlden DR, Chambers SK. When do I know I am cured? Using conditional estimates to provide better information about cancer survival prospects. *The Medical journal of Australia* 2011;194(2):73-7. doi: 10.5694/j.1326-5377.2011.tb04171.x [published Online First: 2011/01/19]
44. Cancer Institute NSW. CanDLe program [Available from: https://www.cancer.nsw.gov.au/research-and-data/cancer-data-and-statistics/candle-program accessed 19/03/2023.
